# How to Hide One’s Relationships from Link Prediction Algorithms

**DOI:** 10.1038/s41598-019-48583-6

**Published:** 2019-08-21

**Authors:** Marcin Waniek, Kai Zhou, Yevgeniy Vorobeychik, Esteban Moro, Tomasz P. Michalak, Talal Rahwan

**Affiliations:** 1 Computer Science, New York University, Abu Dhabi, UAE; 2 Institute of Informatics, University of Warsaw, Warsaw, Poland; 3 Computer Science and Engineering, Washington University in Saint Louis, Saint Louis, USA; 4 Departmento de Matematicas & GISC, Universidad Carlos III de Madrid, Madrid, Spain; 5 Media Lab, Massachusetts Institute of Technology, Cambridge, USA

**Keywords:** Computer science, Computational science

## Abstract

Our private connections can be exposed by link prediction algorithms. To date, this threat has only been addressed from the perspective of a central authority, completely neglecting the possibility that members of the social network can themselves mitigate such threats. We fill this gap by studying how an individual can rewire her own network neighborhood to hide her sensitive relationships. We prove that the optimization problem faced by such an individual is NP-complete, meaning that any attempt to identify an optimal way to hide one’s relationships is futile. Based on this, we shift our attention towards developing effective, albeit not optimal, heuristics that are readily-applicable by users of existing social media platforms to conceal any connections they deem sensitive. Our empirical evaluation reveals that it is more beneficial to focus on “unfriending” carefully-chosen individuals rather than befriending new ones. In fact, by avoiding communication with just 5 individuals, it is possible for one to hide some of her relationships in a massive, real-life telecommunication network, consisting of 829,725 phone calls between 248,763 individuals. Our analysis also shows that link prediction algorithms are more susceptible to manipulation in smaller and denser networks. Evaluating the error vs. attack tolerance of link prediction algorithms reveals that rewiring connections randomly may end up exposing one’s sensitive relationships, highlighting the importance of the strategic aspect. In an age where personal relationships continue to leave digital traces, our results empower the general public to proactively protect their private relationships.

## Introduction

As social network analysis tools become more sophisticated and widespread^1^, it becomes increasingly possible to infer confidential information using data about our social connections, email exchanges, and even financial transactions^2^. This raises privacy and security related concerns as our data may be valuable not only to enterprises and public entities, but also to cyber criminals who are increasingly relying on network analysis tools for malicious purposes^3,4^.

Among the most fundamental network analysis tools are those designed for *link prediction*^5^. Intuitively, such tools analyse the topology of a given network in order to predict the connections that are most likely to form in the future^5^. These tools can also be used to analyse the observed network topology to identify connections that are *hidden* from the observer, either due to data scarcity, or due to the deliberate concealment of information^6^. Link prediction has numerous applications, ranging from providing recommendations in e-commerce^7^, through discovering the interactions between proteins in biological networks^8^, to finding hidden connections between terrorists^9^ or criminals^10^. A plethora of link prediction algorithms have been proposed in the literature^5,11,12^. In this article, we focus on the mainstream class of such algorithms, which is based on *similarity indices*^13^, whereby the topology of a given network is analysed to quantify the similarity between any two disconnected nodes. Here, the underlying assumption is that the greater the similarity between two nodes, the greater the likelihood of having a link between them. These algorithms are extensively studied in the literature due to their scalability, effectiveness, and robustness^13^.

If used with malicious intent, link prediction algorithms may constitute a threat to both the privacy and the security of the general public. In particular, inspired by the proverb “*tell me who your friends are and I’ll tell you who you are*”, a network analyzer may use link prediction algorithms to perform a *link re-identification attack*^14^, which not only reveals your undisclosed friends, but may also enhance the severity of the more general *attribute inference attack*^15^ whereby the goal is to infer various private information about “*who you are*”^16,17^. Worse still, even when link prediction is applied without malicious intentions, it can still have undesirable consequences. Consider the hundreds of millions of Facebook users across the globe who see link prediction in action every time they receive a “*friend suggestion*”. These suggestions become problematic when the “*people you may know*” include individuals you do not want to be associated with, e.g., due to their radical views or extreme ideologies, thereby making you guilty by association.

The issue of protecting link privacy has already been studied in the literature from various angles. More specifically, the network anonymization and de-anonymization literature^18–22^ considered the problem faced by a *data trustee* who publishes or discloses anonymized network data to a third party for business, scientific, or statistical purposes. A straightforward anonymization strategy would be to remove any identifying attributes, and introduce some synthetic identifiers instead. However, such an approach is not immune to attacks, especially in scenarios where an attacker possesses some additional information or background knowledge which, if coupled with the published data, can be used to de-anonymize the network^23,24^. A more effective alternative is to introduce network perturbations by removing existing links and adding false ones. Broadly speaking, two main approaches have been proposed for this purpose^25^. In the first one, the perturbations are introduced *randomly*^26–30^. In the second approach, the perturbations are *constrained* so that the anonymization satisfies certain criteria such as, e.g., *k*-degree anonymity, which is satisfied when the degree of every node is the same as the degree of at least *k* − 1 other nodes^31,32^. Nevertheless, in both approaches, too many modifications may cause the network to lose its fundamental properties and even become worthless to legitimate users. Hence, this literature focuses primarily on how the data trustee should modify the network such that its desired properties are preserved and a satisfactory level of privacy protection is ascertained^26,27,33–36^. An implicit assumption in this literature is that the responsibility of protecting link privacy lies solely on the shoulders of the data trustee, implying that the members of the network are incapable of protecting their undisclosed connections by themselves.

In contrast to the aforementioned literature, in this article we do not consider the issue of protecting link privacy from the perspective of the data trustee but rather from the perspective of a network member. As such, our main goal is to understand how a self-interested individual should act in order to conceal her sensitive relationships. Unlike the data trustee, such a self-interested individual is not the least concerned with anonymizing the entire network nor preserving its properties. Instead, her sole objective is to protect her own privacy, regardless of the consequences on the network as a whole.

In more detail, we consider a setting in which a “*seeker*” runs link prediction algorithms, and a self-interested member of the network, called the “*evader*”, strategically rewires the connections within her neighbourhood in order to hide some of her relationships from the seeker. We study the optimization problem faced by the evader, and prove that it is extremely challenging for her to optimally hide those relationships from some of the most widely used link prediction algorithms. Based on this finding, we shift our attention towards identifying effective, albeit not optimal, solutions. In particular, we propose two heuristics that can readily be applied by users of existing social media platforms, without requiring any knowledge about the topology beyond their network vicinity. The first heuristic *removes* strategically-chosen links from the network, while the other *adds* new ones. We show that both heuristics are effective in practice, although the former is superior to the latter, suggesting that in order to hide a relationship, “unfriending” carefully-chosen individuals can provide a more effective disguise than befriending new ones. Furthermore, we evaluate the attack tolerance of different link prediction algorithms, and find that their resilience tends to increase with the number of nodes, and decrease with the average degree in the network. Finally, we compare the error vs. attack tolerance of link prediction algorithms, to evaluate the performance gain achieved when the evader acts strategically as opposed to randomly. We find that strategic modifications often bring significant gains, while random modifications can backfire, leaving the evader more exposed.

In an age where social media platforms are ingrained in our day-to-day lives, our privacy is becoming ever more vulnerable to invasion, and our ability to take a stand against such intrusion is now more pressing than ever. Keeping certain relationships hidden from everyone else is not as straightforward as it may seem. Indeed, with the widespread use of link prediction algorithms, it is no longer sufficient to simply avoid declaring those relationships. To make matters worse, such algorithms may arrive at erroneous conclusions, associating us with people we may not even know, and potentially tainting our reputation. We address these issues by proposing effective and practical heuristics that can be applied by users of existing social media platforms, thereby demonstrating how to hide one’s relationships from link prediction algorithms.

## Results

### Theoretical analysis

Given an undirected network, *G* = (*V*, *E*), where *V* is the set of nodes and *E* is the set of edges, we will use the term “*non-edge*” to refer to any pair of nodes that is *not* in *E*, and will denote the set of all non-edges by $\bar{E}$. Our problem of *evading link prediction* involves a *seeker* who ranks all non-edges based on a *similarity index* (Section S1), and identifies the highly-ranked ones as edges that are likely to be part of the network, or likely to form in the future. An *evader*, on the other hand, has a set of undeclared relationships that she wishes to keep private; the fact that these relationships are undeclared means that they are *non-edges* as far as the seeker is concerned, and we will model them as such. The evader’s goal is then to rewire the network in order to minimize the likelihood of those non-edges being highlighted by the seeker. Note that a non-edge becomes less exposed to the seeker if it drops in the similarity-based ranking of all non-edges. To quantify the degree to which a non-edge is exposed in any such a ranking, we use two standard performance measures, namely the *area under the ROC curve* (*AUC*)^37^ and the *average precision* (*AP*)^38^ (see Section S2). Intuitively, these performance measures quantify the ability of a similarity index to identify the missing edges in the network. In our context, the missing edges are the undisclosed relationships of the evader, and thus her goal is to *minimize* the performance measures. Formally, the problem faced by the evader is defined as follows:

#### Definition 1

(Evading Link Prediction). *This problem is defined by a tuple*, $(G,{s}_{G},f,H,b,\hat{A},\hat{R})$, *where* $G=(V,E)$ *is a network*, ${s}_{G}:\bar{E}\to {\mathbb{R}}$ *is a similarity index*, $f\in \{AUC,AP\}$ *is a performance evaluation metric*, $H\subset \bar{E}$ *is the set of non-edges to be hidden*, $b\in {\mathbb{N}}$ *is a budget specifying the maximum number of edges that can be modified (i.e., added or removed)*, $\hat{A}\subseteq \bar{E}\backslash H$ *is the set of edges that can be added, and* $\hat{R}\subseteq E$ *is the set of edges that can be removed. The goal is then to identify two sets*, ${A}^{\ast }\subseteq \hat{A}$ *and* ${R}^{\ast }\subseteq \hat{R}$*, such that the resulting set*, ${E}^{\ast }=(E\cup {A}^{\ast })\backslash {R}^{\ast }$*, is in*:

$$\mathop{{\rm{\arg }}\,{\rm{\min }}}\limits_{E^{\prime} \in \{(E\cup A)\backslash R:A\subseteq \hat{A},\,R\subseteq \hat{R},|A|+|R|\le b\}}f(E^{\prime} ,H,{s}_{G}).$$

In this definition, we introduced the budget *b* as well as the sets $\hat{A}$ and $\hat{R}$ to model scenarios in which the evader’s ability to modify the network is limited. The following theorem implies that, given a budget specifying the number of permitted network modifications, it is extremely challenging to identify an *optimal* way to spend this budget in order to hide a given set of non-edges:

#### Theorem 1

. *The problem of Evading Link Prediction is NP-complete for each of the following similarity indices: Common Neighbours*^39^*, Salton*^40^*, Jaccard*^41^*, Sørensen*^42^*, Hub Promoted*^43^*, Hub Depressed*^43^*, Leicht-Holme-Newman*^44^*, Adamic-Adar*^45^ *and Resource Allocation*^46^.

A discussion of the choice of similarity indices can be found in Section S1, while the proof of the theorem can be found in Section S3. Theorem 1 implies that, for any of the indices outlined therein, the problem of evading link prediction is at least as hard as any of the problems in the class NP (Non-deterministic Polynomial-time), meaning that no known algorithm can solve it in polynomial time. Despite this hardness, the situation is not necessarily bleak, especially in situations where a reasonable, albeit not optimal, solution would suffice. With this in mind, we will present two heuristic algorithms that run in polynomial time; the first, called *CTR*, focuses on removing edges whereas the second, called *OTC*, focuses on adding edges.

### The CTR heuristic

Our first heuristic, called CTR (which stands for *Closed-Triad-Removal*) can serve an evader *w* wishing to hide connections in *H* by removing an edge, (*v*, *w*) ∈ *E*, such that:

$$\exists x\in V:((v,x)\in E)\wedge ((x,w)\in H).$$

**Figure 1:**
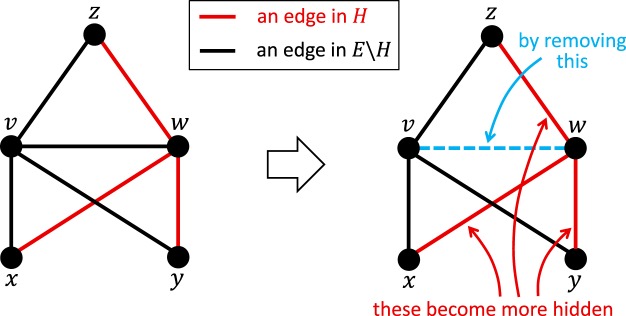
An illustration of the main idea behind the CTR heuristic. Here, by removing (*v*, *w*), we remove from the network three closed triads: one containing the nodes *v*, *w*, *x*, another containing *v*, *w*, *y*, and a third containing *v*, *w*, *z*. Consequently, the similarity scores of (*x*, *w*), (*w*, *y*) and (*w*, *z*) can only decrease based on the analysis in *Materials and Methods*.

By removing (*v*, *w*) from the network, the algorithm *removes the closed triad* whose nodes are *v*, *w* and *x*; hence the name Closed-Triad-Removal (CTR); see the pseudo-code in Section S5. Note that, although *v*, *w* and *x* form a closed triad, this is initially unknown to the seeker since (*x*, *w*) is undeclared, i.e., it is a non-edge as far as the seeker is concerned. Importantly, the removal of (*v*, *w*) can only decrease the similarity score of (*x*, *w*) according to any of the similarity indices outlined in Theorem 1; see the analysis in *Materials and Methods*. The algorithm can be even more effective if the removal of (*v*, *w*) results in the removal of *multiple* closed triads, each containing a non-edge in *H*. In Fig. 1 for example, the removal of (*v*, *w*) decreases the similarity scores of not one, but three non-edges in *H*, namely (*x*, *w*), (*w*, *y*) and (*w*, *z*). Based on this observation, the CTR heuristic is designed to maximize the number of such non-edges, by examining all possible choices of (*v*, *w*) and selecting one that affects the greatest number of edges in *H*.

CTR can readily be applied by users of existing social media platforms. In Fig. 1 for example, if *w* wishes to hide her relationships with *x*, *y*, and *z*, then CTR simply requires *w* to “unfriend” as many people as possible who are friends of *x*, *y* and *z*. This can easily be applied on Facebook for example, where the mutual friends of a person and any of her friends are visible.

### The OTC heuristic

**Figure 2:**
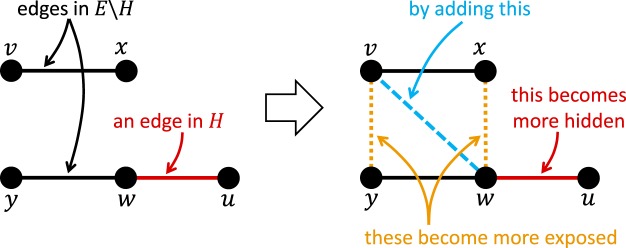
An illustration of the main idea behind the OTC heuristic. Here, the addition of (*v*, *w*) creates two open triads: one contains the nodes *x*, *v*, *w*; the other contains *v*, *w*, *y*. In such situations, the similarity scores of (*x*, *w*) and (*y*, *v*) increase while that of (*w*, *u*) could also decrease; see the analysis in *Materials and Methods*.

Our second heuristic, called OTC (which stands for *Open-Triad-Creation*) works by *adding* edges to the network, unlike CTR which worked by *removing* edges. Generally speaking, the goal of OTC is to “hide” a non-edge, *e* ∈ *H*, by *decreasing* the similarity score of *e* while at the same time *increasing* the similarity scores of (some of) the non-edges that fall within the neighbourhood of *e*. This, in turn, decreases the position of *e* in the similarity-based ranking of all non-edges, thereby reducing the likelihood of *e* being highlighted by a seeker armed with a link prediction algorithm. To achieve this goal, OTC rewires the network as illustrated in Fig. 2. More formally, it selects a non-edge (*v*, *w*) to be added to the network such that:

- $$\exists u\in V:((w,u)\in H)\wedge ((v,u)\notin E)$$;
- $$\exists x\in V:((x,v)\in E)\wedge ((x,w)\in \bar{E}\backslash H)$$.

As shown in Fig. 2, the addition of (*v*, *w*) *creates open triads*–one containing *x*, *v*, *w* and another containing *v*, *w*, *y*—hence the name *Open-Triad-Creation (OTC)*. Importantly, given the similarity indices outlined in Theorem 1, the addition of (*v*, *w*) in Fig. 2 can only decrease the similarity score of (*w*, *u*) and can only increase that of (*x*, *w*) and (*y*, *v*); see *Materials and Methods* for a more formal analysis. More generally, since the creation of an open triad can only increase the similarity score of the non-edge therein, the more open triads we create by adding (*v*, *w*) the better, since this may *increase* the similarity scores of a greater number of non-edges, all of which contribute towards *reducing* the position of (*w*, *u*) in the similarity-based ranking of all non-edges. Based on this observation, OTC examines all possible choices of (*v*, *w*), and selects one that results in the greatest reduction in the ranking of the non-edges in *H*, while ensuring that no other non-edge in *H* becomes more exposed during this process; see the pseudo-code in Section S6.

OTC can be applied on popular social media platforms in a straightforward manner. For instance, if *u* and *w* wish to hide their relationship, then any one of them, say *w*, can send friendship requests to individuals whose list of friends contains as many people as possible who are not connected to *w*. Even if such individuals are hard to find, one can still send random friendship requests to highly-connected strangers, hoping that some of them would accept the request. This is indeed plausible, as an estimated 55% of people accept friendship requests from complete strangers on Facebook^47^. Nevertheless, when evaluating OTC empirically in the next section, it will only be permitted to add edges between the evader and the neighbours of her neighbours, since they are even more likely to accept friendship requests than complete strangers.

### Simulation results

A standard way to evaluate a similarity index is as follows. First, the links of the network are divided into a training set, *T*, and a probe set, *Q*. The index trains on *T* and assigns a similarity score to every pair of nodes accordingly. These scores are then evaluated based on the *area under the ROC curve* (denoted by *AUC*)^37^, which can be interpreted as the probability that the index assigns a greater score to a random link in *Q* than to a random non-edge. With this in mind, we evaluate the effectiveness of each heuristic against a similarity index in a given network as follows: we run the heuristic iteratively, where each iteration involves either removing an edge (when running CTR) or adding an edge (when we run OCT). After each such iteration, we compute *AUC* given a training set consisting of every link in the network and a probe set consisting of every link in *H*; this way we can assess *the probability that the index assigns a greater score to a random link in H than to a random non-edge*. We also consider another standard performance metric, namely the *average precision* (denoted by *AP*)^38^. While this measure is not as intuitive as *AUC*, it also returns a value between 0 and 1, where 1 means that the links in *H* are fully exposed (i.e., the similarity index ranks them higher than any other non-edge), while 0 means that the links in *H* are fully hidden; see Section S2 for more details.

We evaluate the effectiveness of each heuristic against a similarity index in a given network as follows: we run the heuristic iteratively, and after each iteration, we compute *AUC* given a training set consisting of every link in the network and a probe set consisting of every link in *H*; this way we can assess *the probability that the index assigns a greater score to a random link in H than to a random non-edge*. We also consider an alternative performance metric, namely the *average precision* (denoted by *AP*)^38^.

**Figure 3:**
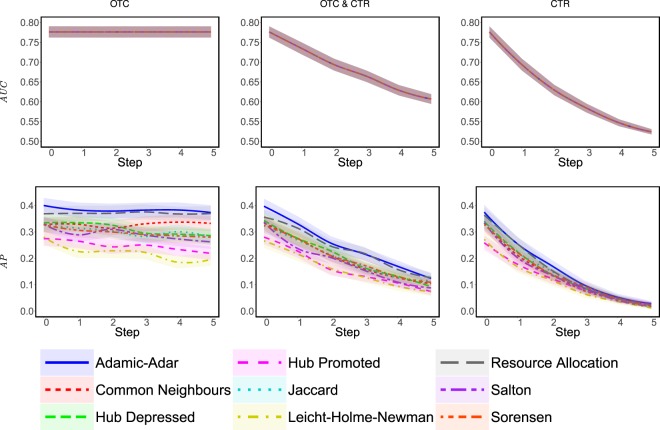
Given different similarity indices and a telecommunication network consisting of 248,763 nodes and 829,725 edges, the figure depicts the average *AUC* and *AP* during the execution of OTC and CTR with a budget *b* = 5. For each similarity index, we consider 10 different, randomly-chosen evaders that have at least 9 connections each (to ensure that no evader is entirely disconnected when running CTR); for each such evader, we create 5 sets of *H*, each consisting of 3 edges chosen randomly from the evader’s connections. This yields a total of 50 experiments, the average of which is reported with the coloured areas representing the 95% confidence intervals.

We start by evaluating the effectiveness of our heuristics in hiding 3 edges of a randomly-chosen evader, *using only 5 modifications to a massive telecommunication network*, consisting of all 829,725 phone calls between the 248,763 users of a particular European telecom operator, who live in four geographically continuous districts^48^. Given different similarity indices, Fig. 3 depicts the results of OTC (which adds edges) and CTR (which removes edges), and also shows what happens when the budget is split between the two heuristics (by alternating between adding and removing edges). As can be seen, the impact of OTC appears to be negligible, CTR seems far more effective, and mixing the two heuristics does not seem to produce any synergistic effects. This suggests that, in order to hide a relationship, one should predominantly focus on “unfriending” strategically-chosen individuals, rather than befriending new ones (similar trends were observed when considering just one instead of four districts; see Section S7.4). We evaluated the two heuristics against other similarity indices, using a variety of networks that are much smaller than the aforementioned one, and found that OTC becomes effective given smaller networks, but CTR remains superior; see Section S7.

**Figure 4:**
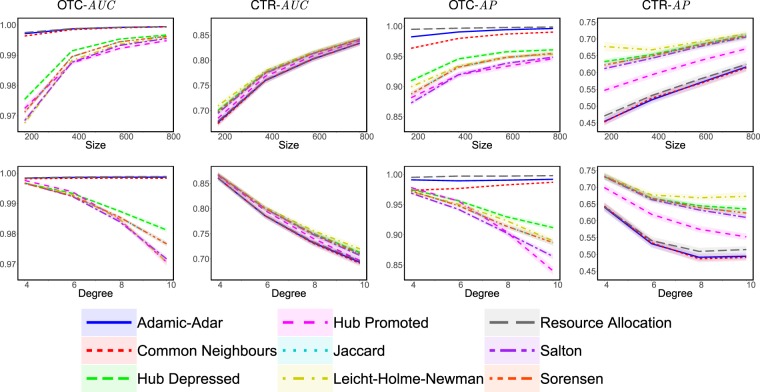
Evaluating the attack tolerance of different similarity indices against OTC and CTR in scale-free networks, while varying the number of nodes, *n* ∈ {200, 400, 600, 800}, and the average degree, *d* ∈ {4, 6, 8, 10}, where the attack tolerance is measured in terms of the relative change in *AUC* and *AP*. Specifically, for each combination of *n* and *d* we generate 50 scale-free networks; for each such network, *G*, we randomly select 10 evaders (each of which has at least 9 connections); for each such evader, *v*, we create 5 different sets *H*, each consisting of 3 random edges of *v*. Each instance of (*G*, *v*, *H*) constitutes a separate experiment. The results are reported as an average over different values of *d* for each *n* in the first row of subfigures, and as an average over different values of *n* for each *d* in the second row. Each point in each plot corresponds to an average over 10,000 experiments, with coloured areas representing the 95% confidence intervals.

Next, we evaluate the attack tolerance of the similarity indices outlined in Theorem 1 based on the two performance metrics—*AUC* and *AP*—while varying the number of nodes, *n*, and the average degree, *d*, in Scale-Free networks; see Fig. 4. Overall, the attack tolerance of those similarity indices tends to increase with *n* and decrease with *d*. Similar trends were observed when experimenting with Small-World networks and Erdos-Renyi random graphs; see Section S8. This suggests that it is harder to hide one’s connections in larger and sparser networks, which is particularly alarming given our increasing reliance on social media platforms, with which we become embedded in networks of unprecedented scale and sparsity.

**Figure 5:**
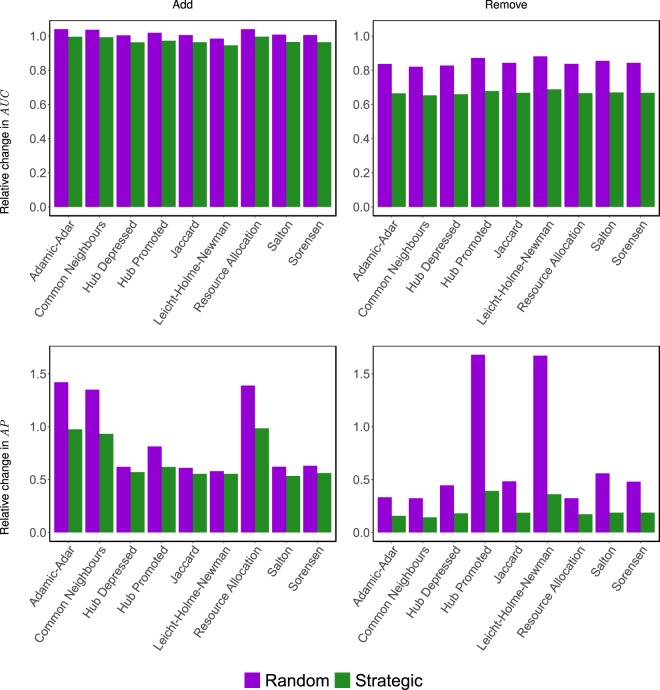
Comparing the *error* vs. *attack* tolerance of different similarity indices, by comparing the impact of modifying links that are chosen *randomly* against those that are chosen *strategically* by one of our heuristics. Specifically, for each network outlined in Section S7.1, we randomly select 10 evaders that have at least 9 connections each (this ensures that any such evader will not become entirely disconnected when running CTR). For each evader, we randomly select five instances of *H*, each containing 3 edges selected randomly from the evader’s connections. For each such *H*, we run experiments with a budget *b* = 5. This budget is then spent either randomly, or by one of our heuristics. The results depicted in the figure are averaged over all considered networks (for randomly generated networks, the experiment is repeated over 50 instances). We disregard any *H* for which the performance metric is below 0.001, and assume the edges therein to be hidden already.

So far, we have shown that the evader can hide some of her connections by rewiring edges in her network neighbourhood following our heuristics. However, it is still unclear whether these results are due to rewiring *carefully-chosen* edges, or whether the same results can be obtained by rewiring *any* edges in this neighbourhood. With this in mind, we compare the *error* vs. *attack* tolerance of the similarity indices outlined in Theorem 1. In particular, we compare the impact of modifying links that are chosen *randomly*—as is the case in the work of Zhang *et al*.^49^—against those that are chosen *strategically* by one of our heuristics. Here, the random links to be modified are chosen from the same set of links that our heuristics are allowed to modify. The result of *adding* random links is compared against OTC (which *adds* links), whereas the results of *removing* random links is compared against CTR (which *removes* links). By doing so, the outcome of random modifications can serve as a baseline for evaluating the performance gain achieved when the evader acts strategically. As can be seen in Fig. 5, regardless of the link prediction algorithm being used by the seeker, there is a marked increase in the effectiveness of hiding when the rewiring is carried out strategically rather than randomly. This difference is clearly more pronounced when the edges are being removed from, rather than added to, the evader’s neighbourhood. Also noteworthy is the fact that random changes can actually make the links of interest more exposed (see how, in many cases, the result of random changes is greater than 1). To put it differently, acting randomly can backfire and end up compromising the evader’s privacy.

Finally, we study how our heuristics affect certain properties of each of the networks outlined in Section S7.1. These properties are: (i) average degree; (ii) degree correlation; (iii) global clustering coefficient; (iv) local clustering coefficient; (v) size of the giant connected component. The results can be found in Section S9. Broadly speaking, the changes in the network properties are mostly negligible, and diminish as the number of nodes increases.

## Discussion

Arguably, hundreds of millions of Facebook users are familiar with the experience of receiving “friend suggestions”, and many recipients of such suggestions are left wondering how Facebook is able to predict relationships that were never disclosed online. These suggestions are guided by link prediction algorithm which, if used with malicious intent, can violate our basic right to choose which of our connections to disclose. Although this privacy concern has already been addressed in the literature, these studies implicitly assume the role of a central authority, while completely neglecting the possibility that members of the social network can themselves mitigate such threats. We fill this gap by developing heuristics that empower the general public, by offering them a readily-applicable way to conceal any connections they deem sensitive, without requiring them to know the topology beyond their network neighbourhood. While we prove that identifying an *optimal* way to hide such connections is intractable, the empirical evaluation demonstrates that our heuristics are effective in practice, and reveals that it is more beneficial to focus on “unfriending” carefully-chosen individuals rather than befriending new ones. Our analysis also shows that link prediction algorithms are more susceptible to manipulation in smaller networks and in networks with higher density. Evaluating the error vs. attack tolerance of link prediction algorithms reveals that the choice of connections to modify is critical, since making random choices may actually backfire and end up exposing, rather than hiding, the connections in question.

It should be underlined that the applicability of our heuristics depends on the information available to the evader and the seeker. In particular, while our heuristics can be applied when the evader knows the ego network of the other end of the sensitive relationship, such knowledge cannot be taken for granted, especially if the evader is engaged in a one-sided effort to conceal the relationship. As for the information available to the seeker, it may include not only a snapshot of the social network, but also a history of the modifications made therein. Thus, even if the evader manages to rewire the network and conceal a sensitive relationship, the seeker may still retrieve the original topology, thereby rendering the evader’s actions futile. In such cases, our heuristic may still be implemented by interpreting the removal of a link to mean the avoidance of creating that link, e.g., in our telecommunication example, removing a link may be interpreted as avoiding the corresponding phone call in the first place.

All in all, our findings demonstrate that, in an age where people continue to lose ownership of their personal data, individuals can take a stand and at least to some extent shield their relationships from privacy invasion.

## Methods

Let *N*_*G*_(*v*) denote the set of neighbours of node *v*, i.e., *N*_*G*_(*v*) = {*w* ∈ *V*:(*v*, *w*) ∈ *E*}, and let *N*_*G*_(*v*, *w*) denote the set of common neighbours of *v* and *w*, i.e., *N*_*G*_(*v*, *w*) = *N*_*G*_(*v*) ∩ *N*_*G*_(*w*). The *degree* of *v* will be denoted by *d*_*G*_(*v*), i.e., *d*_*G*_(*v*) = |*N*_*G*_(*v*)|. Whenever it is clear from the context, we will omit the graph subscript, e.g., by writing *N*(*v*) instead of *N*_*G*_(*v*). Now, let ${\mathscr{S}}$ denote the set of all the similarity indices outlined in Theorem 1; the formula for each of these indices is specified in Section S1. Looking at these formulae, one can see that the similarity score of every non-edge, $(x,w)\in \bar{E}$, depends solely on (some of) the following factors:

- *Factor 1: the number of common neighbours of the non-edge*. More specifically, for every $s\in {\mathscr{S}}$, the score *s*(*x*, *w*) *increases* with |*N*(*x*, *w*)|.
- *Factor 2: the degree of each end of the non-edge, but only if both ends have some common neighbours*. Specifically, for every similarity index, $s\in {\mathscr{S}}\backslash \{{s}^{{\rm{CN}}},{s}^{{\rm{AA}}},{s}^{{\rm{RA}}}\}$, the score *s*(*x*, *w*) *decreases* with *d*(*x*) and with *d*(*w*) if *N*(*x*, *w*) ≠ ∅.^a^^1^ Otherwise, if *N*(*x*, *w*) = ∅, then *s*(*x*, *w*) is not affected by *d*(*x*) nor by *d*(*w*). As for the remaining similarity indices, i.e., those in {*s*^CN^, *s*^AA^, *s*^RA^}, their scores are not affected by *d*(*x*) nor by *d*(*w*), regardless of whether *N*(*x*, *w*) = ∅.
- *Factor 3: the degree of every common neighbour of the non-edge*. More specifically, for every similarity index *s* ∈ {*s*^AA^, *s*^RA^} and every common neighbour *v* ∈ *N*(*x*, *w*), the score *s*(*x*, *w*) *decreases* with *d*(*v*). As for the remaining similarity indices, i.e., those in ${\mathscr{S}}\backslash \{{s}^{{\rm{AA}}},{s}^{{\rm{RA}}}\}$, their scores are not affected by any *d*(*v*):*v* ∈ *N*(*x*, *w*).

Therefore, the *addition* of an edge, (*v*, *w*), can only affect the scores of the following types of non-edges:

- *Type 1:* (*x*, *w*):*x* ∈ *N*(*v*)\*N*(*w*). Such a non-edge is affected by the addition of (*v*, *w*), which adds *v* to *N*(*x*, *w*), thereby increasing |*N*(*x*, *w*)|. This, in turn, *increases s*(*x*, *w*) for every similarity index $s\in {\mathscr{S}}$; see *Factor 1*.
- *Type 2:* (*x*, *w*):*N*(*x*, *w*) ≠ ∅. Such a non-edge is affected by the addition of (*v*, *w*), which increases *d*(*w*). This, in turn, *decreases s*(*x*, *w*) for every $s\in {\mathscr{S}}\backslash \{{s}^{{\rm{CN}}},{s}^{{\rm{AA}}},{s}^{{\rm{RA}}}\}$; see *Factor 2*.
- *Type 3:* (*x*, *y*): *x*, *y* ∈ *N*(*w*). Such a non-edge is affected by the addition of (*v*, *w*), which increases the degree of a common neighbour of *x* and *y*, namely *w*. This, in turn, *decreases s*(*x*, *y*) for every *s* ∈ {*s*^AA^, *s*^RA^}; see *Factor 3*.

Note that a non-edge (*x*, *v*) can be of both *Type 1* and *Type 2* simultaneously; this happens when *x* ∈ *N*(*w*)\*N*(*v*) and *N*(*x*, *v*) ≠ ∅. In this case, (*x*, *v*) is affected by *Factor 1*—which increases *s*(*x*, *v*)—as well as *Factor 2*—which decreases *s*(*x*, *v*). Since these two factor have opposite effects, whether *s*(*x*, *v*) increases depends on whether the effect of *Factor 1* outweighs that of *Factor 2*.

Finally, note that the impact of removing (*v*, *w*) is exactly the opposite to that of adding (*v*, *w*). For instance, suppose that (*v*, *x*) is a non-edge of Type 1 and not of Type 2. Then, by adding (*v*, *w*) to a network $(V,E):(v,w)\notin E$, we *increase s*(*v*, *x*) for every $s\in {\mathscr{S}}$. In contrast, by removing (*v*, *w*) from a network $(V,E):(v,w)\in E$, we *decrease s*(*v*, *x*).

With these observations in mind, let us analyse our heuristics. Recall that CTR removes an edge, (*v*, *w*) ∈ *E*, where $\exists x\in V:((v,x)\in E)\wedge ((x,w)\in H)$. Importantly, by removing (*v*, *w*):

- the node *v* is removed from the common neighbours of *w* and *x*, thereby reducing |*N*(*x*, *w*)|. As a result, the similarity score of (*x*, *w*) *decreases* according to *Factor 1*.
- the degree of node *w* decreases. As a result, the similarity score of (*x*, *w*) can only *increase* according to *Factor 2*.

In other words, by removing (*v*, *w*), the similarity score of (*x*, *w*) is subjected to two opposing forces; one that decreases it, and another that increases it, Nevertheless, the following theorem implies that the latter force never outweighs the former one. In other words, by removing (*v*, *w*), the similarity score of (*x*, *w*) can only *decrease* given the similarity indices in ${\mathscr{S}}$; see the proof in Section S4.

### Theorem 2

. *Let* $G^{\prime} =(V,E^{\prime} )$ *be a network, and let (x, w) be a non-edge in G*′*. Furthermore, let v be a node in G*′ *such that* $v\in {N}_{G^{\prime} }(x)$ *and* $v\notin {N}_{G^{\prime} }(w)$*. Finally, let G be the network that results from adding (v, w) to G*′*, i.e*., $G=(V,E)$ *where* $E=E^{\prime} \cup \{(v,w)\}$*. Then, for every similarity index*, $s\in {\mathscr{S}}$*, we have*:

$${s}_{G\text{'}}(x,w)\le {s}_{G}(x,w)$$

Moving on to OTC, recall that this heuristic adds to the network a non-edge (*v*, *w*) such that, *after the addition of* (*v*, *w*):

- $$\exists u\in V:((w,u)\in H)\wedge ((v,u)\notin E)$$;
- $$\exists x\in V:(\{(x,v),(v,w)\}\subseteq E)\wedge ((x,w)\in \bar{E}\backslash H)$$.

Based on this, by adding (*v*, *w*):

- the degree of *w* increases, which can only decrease the similarity score of (*w*, *u*) according to *Factor 2*.
- the similarity scores of (*x*, *w*) and (*y*, *v*) can only increase according to Theorem 2.

Thus, given the similarity indices in ${\mathscr{S}}$, the addition of (*v*, *w*) can only decrease the position of (*w*, *u*) in the similarity-based ranking of all non-edges.

## Supplementary information

Supplementary Materials

## Footnotes

1 1The *Jaccard* index^41^, *s*^Jac^, is affected by *d*(*x*) and *d*(*w*), since: $|N(x){\cup }^{}N(w)|=d(x)+d(w)-|N(x,w)|$.
