# Supplementary material for: How to Hide One’s Relationships from Link Prediction Algorithms
Source: Sci Rep. 2019 Aug 21;9:12208. doi: 10.1038/s41598-019-48583-6 (PMC6704149; doi:10.1038/s41598-019-48583-6)
Supplement: Supplementary file 1 — Supplementary Materials [file 41598_2019_48583_MOESM1_ESM.pdf]

Supplementary Materials for

# How to Hide One's Relationships from Link Prediction Algorithms

Marcin Waniek, Kai Zhou, Yevgeniy Vorobeychik, Esteban Moro,
Tomasz P. Michalak*, Talal Rahwan*

* Corresponding authors. E-mail: tpm@mimuw.edu.pl, talal.rahwan@nyu.edu

This document is structured as follows:

- **Section S1** (*page 2*) presents link prediction algorithms;
- **Section S2** (*page 4*) presents performance evaluation metrics;
- **Section S3** (*page 6*) presents the proofs of NP-completeness of the Evading Link Prediction problem:
  - **Section S3.1** presents a proof using edge *addition*;
  - **Section S3.2** presents a proofs using edge *removal*;
- **Section S4** (*page 27*) presents the proof of Theorem 4;
- **Section S5** (*page 29*) presents the pseudocode of CTR;
- **Section S6** (*page 31*) presents the pseudocode of OTC;
- **Section S7** (*page 33*) evaluates the effectiveness of CTR and OTC. More specifically:
  - **Section S7.1** (*page 33*) describes the networks considered in our experiments;
  - **Section S7.2** (*page 34*) evaluates our heuristics against *local* similarity indices;
  - **Section S7.3** (*page 40*) evaluates our heuristics against *global* similarity indices;
  - **Section S7.4** (*page 51*) presents results for a practical telecommunications scenario and other large-scale real-life networks;
- **Section S8** (*page 54*) evaluates the attack tolerance of different similarity indices;
- **Section S9** (*page 58*) evaluates the impact of running OTC and CTR on the properties of the network;
- **Section S10** (*page 61*) evaluates the impact of running OTC and CTR on the centrality of the evader.

# S1 Link Prediction Algorithms

For any network, and any pair of nodes that are not connected in that network, a link prediction algorithm estimates the likelihood that there exists a not-yet-discovered edge between those two nodes, or that an edge will form between the two nodes in the future [10]. Many link prediction algorithms are based on *similarity indices*, also known as *kernels* [27]. Formally, given a network, $G = (V, E)$, a *similarity index* is a function, $s_G : \bar{E} \to \mathbb{R}$, that assigns to each non-edge $(v, w) \in \bar{E}$ a score indicating the probability of $(v, w)$ forming in the future, or the probability of $(v, w)$ being a not-yet-discovered edge in the network [10]. For any similarity index, $s_G$, and any non-edge, $(v, w) \in \bar{E}$, we will often write $s_G(v, w)$ instead of $s_G((v, w))$ to improve readability, and we will omit the graph subscript when it is clear from the context. Furthermore, following common practice in the literature, we will not consider self-loops, i.e., edges or non-edges of the form $(v, v) : v \in V$.

## S1.1 Local Similarity Indices

An important class of link prediction algorithms are those based on *local* similarity indices, i.e., indices that account for only *local information* pertaining to the non-edge in question. As such, the algorithms based on local similarity indices are typically computationally tractable and can be used even with massive networks. In our study, we consider the following local similarity indices, taken from the survey by Lü and Zhou [21]:[1]

- *Common Neighbours* [24]: $s^{\mathrm{CN}}(v, w) = |N(v, w)|$
- *Salton* [26]: $s^{\mathrm{Sal}}(v, w) = \frac{|N(v,w)|}{\sqrt{d(v)d(w)}}$
- *Jaccard* [13]: $s^{\mathrm{Jac}}(v, w) = \frac{|N(v,w)|}{|N(v) \cup N(w)|}$
- *Sørensen* [28]: $s^{\mathrm{Sør}}(v, w) = \frac{2|N(v,w)|}{d(v)+d(w)}$
- *Hub Promoted* [25]: $s^{\mathrm{HPI}}(v, w) = \frac{|N(v,w)|}{\min(d(v),d(w))}$
- *Hub Depressed* [25]: $s^{\mathrm{HDI}}(v, w) = \frac{|N(v,w)|}{\max(d(v),d(w))}$
- *Leicht-Holme-Newman* [19]: $s^{\mathrm{LHN}}(v, w) = \frac{|N(v,w)|}{d(v)d(w)}$
- *Adamic-Adar* [1]: $s^{\mathrm{AA}}(v, w) = \sum_{u \in N(v,w)} \frac{1}{\log(d(u))}$
- *Resource Allocation* [33]: $s^{\mathrm{RA}}(v, w) = \sum_{u \in N(v,w)} \frac{1}{d(u)}$

The set consisting of all those similarity indices will be denoted by $\mathcal{S}$. More formally:

$$\mathcal{S} = \{s^{\mathrm{CN}}, s^{\mathrm{Sal}}, s^{\mathrm{Jac}}, s^{\mathrm{Sør}}, s^{\mathrm{HPI}}, s^{\mathrm{HDI}}, s^{\mathrm{LHN}}, s^{\mathrm{AA}}, s^{\mathrm{RA}}\}.$$

## S1.2 Global Similarity Indices

Another important class of link prediction algorithms are those categorized by Lü and Zhou [21] as *global* similarity indices. Before presenting those indices, we need to introduce some additional notation. Let $A$ denote the adjacency matrix of a network, let $\lambda^*$ denote the largest eigenvalue of the adjacency matrix, let $L^+$ denote the pseudoinverse of the Laplacian matrix, and let $I$ denote a unit matrix. Now, for any global similarity index, $s$, let $S$ denote the corresponding *similarity matrix*, whereby the similarity of any pair of

[1] The only local similarity index in [21] that is excluded from our analysis is the Preferential Attachment Index. Unlike the other indices in [21], the Preferential Attachment index is based on the assumption that the degree distribution follows a power law—an assumption that does not hold for many of the networks on which we conduct our experiments.

nodes, $v_i, v_j \in V$, is specified at the $i$-th row and $j$-th column of $S$. More formally, $\forall v_i, v_j \in V,\ s(v_i, v_j) = S_{i,j}$. With this notation in place, we can now present the global similarity indices outlined in [21]:

- *Katz* [15] is based on the number of paths between the two nodes, where longer paths are taken with lesser weight according to the dampening factor. Formally, the similarity matrix of this index is:

$$S^{\text{Katz}} = (I - \beta A)^{-1} - I,$$

  where $\beta$ is the dampening factor. In our experiments we set $\beta = \frac{1}{2\lambda^*}$, as the value has to be smaller than the reciprocal of the largest eigenvalue of the adjacency matrix.

- *Leicht-Holme-Newman Global* [19] is based on the idea that two nodes are similar if their neighbourhoods are similar. More formally, the similarity matrix of this index is:

$$S^{\text{LHNG}} = 2|E|\lambda^* D^{-1}(I - \frac{\phi A}{\lambda^*})^{-1} D^{-1},$$

  where $D$ is the degree matrix, i.e., a diagonal matrix where $D_{i,i} = d(v_i)$, and $\phi$ is a free parameter. In our experiments we set $\phi = \frac{97}{100}$, as in the original article.

- *Average Commute Time* [11] is based on the assumption that two nodes are more similar if a random walker can travel between them in a shorter average time. Formally, it is defined as follows:

$$s^{\text{ACT}}(v_i, v_j) = \frac{1}{L^+_{i,i} + L^+_{j,j} - 2L^+_{i,j}}.$$

- *Cosine* [9] is based on the cosine of the angle between the vectors representing the two nodes. More formally, it is defined as follows:

$$s^{\text{Cos}}(v_i, v_j) = \frac{L^+_{i,j}}{\sqrt{L^+_{i,i} L^+_{j,j}}}.$$

- *Random Walk with Restart* [4] is based on the idea that node $v_i$ is more similar to node $v_j$ if node $v_i$ is visited with higher frequency by a random walker who starts at node $v_j$ and iteratively moves to a random neighbor with probability $c$ and returns to node $v_j$ with probability $1-c$. Formally, this index is defined as follows:

$$s^{\text{RWR}}(v_i, v_j) = Q_{i,j} + Q_{j,i},$$

  with matrix $Q$ being:

$$Q = (1 - c)(I - cP^T)^{-1},$$

  where $P$ is the transition matrix: $P_{i,j} = \frac{1}{d(v_i)}$ if $v_j \in N(v_i)$ and $P_{i,j} = 0$ otherwise. In our experiments we set $c = \frac{3}{4}$.

- *SimRank* [14] is based on the idea that two nodes are more similar if two random walkers starting at those nodes are expected to meet faster. This index can be computed iteratively as follows:

$$s^{\text{SR}}(v_i, v_j) = \frac{c \sum_{v \in N(v_i)} \sum_{w \in N(v_j)} s^{\text{SR}}(v, w)}{d(v_i) d(v_j)},$$

  where $\forall v \in V s^{\text{SR}}(v, v) = 1$ and $c$ is the decay factor. In our experiments we set $c = \frac{8}{10}$.

- *Matrix Forest Index* [5] assumes that two nodes are more similar if there is a higher probability that they belong to the same tree in a spanning rooted forest. Formally, the similarity matrix of this index is defined as follows:

$$S^{\text{MFI}} = (I + L)^{-1},$$

  where $L$ is the Laplacian matrix.

## S2 Performance Evaluation Metrics

Arguably, the most common metrics for evaluating the performance of a similarity index are: *Area under the ROC curve (AUC)* [8] and *Area under the Precision-Recall curve (PR)* [22]. To compute any of these metrics for a given similarity index, $s$, we are given a training set, $E$, and a probe set, $Q$, such that $E \cap Q = \emptyset$, i.e., $Q \subset \bar{E}$. The probe set $Q$ is considered the correct solution of link prediction, i.e., similarity indices are expected to assign high scores to non-edges from $Q$. The network $(V, E)$ serves as input to the similarity index, $s$, which produces a ranking of the elements of $\bar{E}$. One can express the quality of this ranking using either $AUC$ or *PR*. To explain how these metrics are computed, we need some additional notation. Let $\sigma_k$ denote the top $k$ elements of $\bar{E}$ when ranked according to $s$, and let $X = \bar{E} \setminus Q$. Next, we explain how $AUC$ or *PR* are computed, and then explain an alternative metric called *average precision* ($AP$).

**Area under the ROC curve ($AUC$):** For any given $E$ and $Q$, $AUC(E, Q, s)$ is the area under the plot consisting of the following points:

$$\left\{ \left( \frac{|\sigma_k \cap X|}{|X|}, \frac{|\sigma_k \cap Q|}{|Q|} \right) \right\}_{k=1}^{|\bar{E}|}$$

$AUC(E, Q)$ can be interpreted as the probability that the similarity index, $s$, assigns a greater score to a randomly chosen non-edge from $Q$ than to a randomly chosen non-edge from $X$ (ties broken at random), i.e.:

$$AUC(E, Q, s) = \frac{|\{(e_1, e_2) \in Q \times X : s(e_1) > s(e_2)\}| + \frac{1}{2}|\{(e_1, e_2) \in Q \times X : s(e_1) = s(e_2)\}|}{|Q||X|}.$$

**Area under the Precision-Recall curve (*PR*):** For any given $E$ and $Q$, $PR(E, Q, s)$ is the area under the plot consisting of the following points:

$$\left\{ \left( \frac{|\sigma_k \cap Q|}{|Q|}, \frac{|\sigma_k \cap Q|}{k} \right) \right\}_{k=1}^{|\bar{E}|}$$

**Average precision ($AP$):** Since the *PR* value is not well-defined for plots that are not continuous, we use instead the *average precision*, $AP$, described by Boyd et al. [3] as one of the most robust estimators of the area under the Precision-Recall curve. Taking into account the possibility of equal scores, the average precision value is computed as follows:

$$AP(E, Q, s) = \frac{1}{|Q|} \sum_{\widehat{e} \in Q} \frac{|\{e \in Q : s(e) > s(\widehat{e})\}| + 1 + \frac{1}{2}|\{e \in Q \setminus \{\widehat{e}\} : s(e) = s(\widehat{e})\}|}{|\{e \in \bar{E} : s(e) > s(\widehat{e})\}| + 1 + \frac{1}{2}|\{e \in \bar{E} \setminus \{\widehat{e}\} : s(e) = s(\widehat{e})\}|}.$$

To understand the intuition behind $AP$, we need to introduce the notion of a "classifier", which takes as input a particular position, $\alpha$, in the similarity-based ranking of all non-edges, and classifies every non-edge below $\alpha$ as an element of $X$, and every other non-edge as an element of $Q$. Based on this notion, $AP$ can be defined as the average "precision" of such a classifier, taken over every value of $\alpha$ that corresponds to a position of a non-edge in $Q$, where "precision" is defined as the proportion of non-edges that are *correctly identified* as elements of $Q$ out of all those identified as elements of $Q$. This way, an ideal similarity index (which ranks every non-edge in $Q$ higher than every non-edge in $X$) would get an $AP$ score of 1 (the maximum possible score). This is because, regardless of the choice of $\alpha$, all non-edges classified as elements of $Q$ would indeed be elements of $Q$.

Let us now give an intuitive characterization of the situations where $AUC$ and $AP$ perform qualitatively differently, i.e., where a certain change in the ranking of elements of $\bar{E}$ causes one of these metrics to increase, while at the same time causing the other metric to decrease. Consider a network with only two hidden edges ($|Q| = 2$) such that, before the hiding process, these two edges have positions $a$ and $b$ (such that $a < b$) in

the ranking of the elements of $\bar{E}$. For simplicity, assume that no two elements in the ranking have the same score. In such a case, the value of $AUC$ before the hiding process is:

$$AUC_1 = \frac{(|X| - a + 1) + (|X| - b + 2)}{2|X|} = \frac{2|X| - a - b + 3}{2|X|}$$

while the value of $AP$ before the hiding process is:

$$AP_1 = \frac{1}{2}\left(\frac{1}{a} + \frac{2}{b}\right).$$

Now assume that, as a result of some rewiring of the network, the position of the first hidden edge increased to $a - 1$ (i.e., it became more exposed), while the position of the second hidden edge decreased to $b + 2$ (i.e., it became more hidden). Arguably, it is not obvious whether the set consisting of these two edges as a whole become more hidden or more exposed as a result of the rewiring, since one of these edges became more hidden, while the other became more exposed. Now, let us see how $AUC$ and $AP$ have changed as a result of the rewiring. Starting with $AUC$, it is now equal to:

$$AUC_2 = \frac{(|X| - a + 2) + (|X| - b)}{2|X|} = \frac{2|X| - a - b + 2}{2|X|}$$

In contrast, $AP$ is now equal to:

$$AP_2 = \frac{1}{2}\left(\frac{1}{a-1} + \frac{2}{b+2}\right).$$

Thus, the change in $AUC$ is equal to:

$$\Delta AUC = AUC_2 - AUC_1 = \frac{2|X| - a - b + 2}{2|X|} - \frac{2|X| - a - b + 3}{2|X|} = -\frac{1}{2|X|}$$

while the change is $AP$ is equal to:

$$\Delta AP = AP_2 - AP_1 = \frac{1}{2}\left(\frac{1}{a-1} + \frac{2}{b+2}\right) - \frac{1}{2}\left(\frac{1}{a} + \frac{2}{b}\right) = \frac{b^2 + 2b + 4a - 4a^2}{2ab(a-1)(b+2)}.$$

This implies that, for any $a$ and $b$, we have $\Delta AUC < 0$, i.e., $AUC$ always decreases after this kind of rewiring. In contrast,, for some values of $a$ and $b$ (in particular for small $b$ and large $b$) we have $\Delta AP > 0$, i.e., $AP$ can increase after this kind of rewiring. This shows that $AUC$ and $AP$ express different notions of the quality of the ranking. While for $AUC$ exposing one of the edges is easily compensated by hiding another edge, the same is not always true for $AP$, as there are cases when exposing one edges guarantees an increase in $AP$.

# S3 NP-completeness of the Evading Link Prediction Problem

In this section, we present two different proofs of NP-completeness of the problem of Evading Link Prediction. The first proof uses edge addition (Section S3.1), while the other uses edge removal (Section S3.2).

## S3.1 Proof of NP-completeness Using Edge Addition

In this subsection, using edge addition, we will prove that the problem of Evading Link Prediction is NP-complete for all the similarity indices described in Section S1.1, and that is for both the $AUC$ and $AP$ metrics which were described in Section S2. To help guide the reader through the process of proving NP-completeness of the problem, we now present an outline of the proof and the general idea behind it.

- We will prove the NP-completeness of the problem of Evading Link Prediction by showing a reduction from the 3-Set Cover problem, a well known NP-complete problem. In order to do this, we have to show that for any instance $(U, P, k)$ of the 3-Set Cover problem we can construct a corresponding instance of the Evading Link Prediction problem, that has a solution if and only if the original instance $(U, P, k)$ has a solution. This way, if there would exist an efficient algorithm of solving the problem of Evading Link Prediction, there would also exist an efficient algorithm of solving the 3-Set Cover problem. However, we show that this is not the case (unless P=NP). Hence, by contradiction, the problem of Evading Link Prediction must be NP-complete.

- A key element of the constructed instance of the Evading Link Prediction problem—which corresponds to a given instance $(U, P, k)$ of the 3-Set Cover problem—is the network structure. In Definition 1 we present a construction of a network denoted $\Gamma(c, P)$, which encapsulates the structure of the given instance of the 3-Set Cover problem. In this network, there exists a node $P_i$ for every set $P_i$, and a node $u_i$ for every element of the universe $u_i$ in the 3-Set Cover problem instance. Importantly, the structure of connections between the nodes reflects the composition of the sets in the 3-Set Cover problem instance, i.e., there exists an edge between the nodes $P_i$ and $u_j$ if an only if the element $u_j$ is a part of the set $P_i$.

- In Lemma 1 we show how the addition of an edge $(P_i, v_0)$ to the network $\Gamma(c, P)$ affects the ranking of non-edges according to any similarity index $s$. In particular, we focus on the ranking of the edge that the evader is trying to hide, namely $(u_0, v_0)$. We show that adding a set of edges $A \subseteq \{(P_i, v_0) : P_i \in P\}$ (corresponding to selecting sets $P_i$ in the 3-Set Cover problem instance) causes the ranking of the hidden edge $(u_0, v_0)$ to drop by the number of elements $u_j$ covered by the sets in $A$.

- Finally, in the proof of Theorem 1 we use the $\Gamma(c, P)$ network, as well as Lemma 1, in order to prove the NP-completeness of the problem of Evading Link Prediction. In particular, we show that the evaluation metric, be it $AUC$ or $AP$, is minimized when all elements of the universe are covered by the sets corresponding to the nodes $P_i$ the appear in $A$. Hence, the optimal solution to the constructed instance of the problem of Evading Link Prediction corresponds to the optimal solution of the given instance of the 3-Set Cover problem.

Having presented the outline of the proof of NP-completeness of the problem, we now move to defining the network $\Gamma(c, P)$ which will be used later on in our proofs.

**Definition 1** (The $\Gamma(c, P)$ Network)**.** *Let $U = \{u_1, \dots, u_m\}$ be a set of $m$ elements, and let $P = \{P_1, \dots, P_q\}$ be a cover of $U$ containing $q$ subsets that are each smaller than $U$. That is, $\forall_i P_i \subset U$ and $\bigcup_{P_i \in P} P_i = U$. Then, given a constant, $c \in \mathbb{N}$, the network $\Gamma(c, P)$ is created as follows:*

- ***The set of nodes:**: For every $P_i \in P$, we create a single node, denoted by $P_i$. Moreover, for every $u_i \in \{u_0, \dots, u_m\}$, we create a node denoted by $u_i$, as well as $c$ nodes denoted by $a_{i,1}, \dots, a_{i,c}$, and $q - |P(u_i)|$ nodes denoted by $d_{i,1}, \dots, d_{i,q-|P(u_i)|}$, where $P(u_i) = \{P_j \in P : u_i \in P_j\}$. Additionally, we create two nodes, $v_0$, and $v_1$.*

- ***The set of edges:*** *For every $P_j \in P$ we create the edge $(P_j, v_1)$, as well as the edges $(P_j, u_i)$ for every $u_i \in P_j$. Moreover, for every $u_i \in U \cup \{u_0\}$ we create the edge $(u_i, v_1)$, as well as the edges $(u_i, u_j)$ for every $u_j \in \{u_{i+1}, \dots, u_m\}$ (this way, the nodes in $\{u_0, \dots, u_m\}$ form an $(m+1)$-clique). Furthermore, for every $d_{i,j}$ we create the edges $(d_{i,j}, u_i)$ and $(d_{i,j}, v_1)$. Finally, for every $a_{i,j}$ we create the edges $(a_{i,j}, u_i)$, $(a_{i,j}, v_0)$ and $(a_{i,j}, v_1)$.*

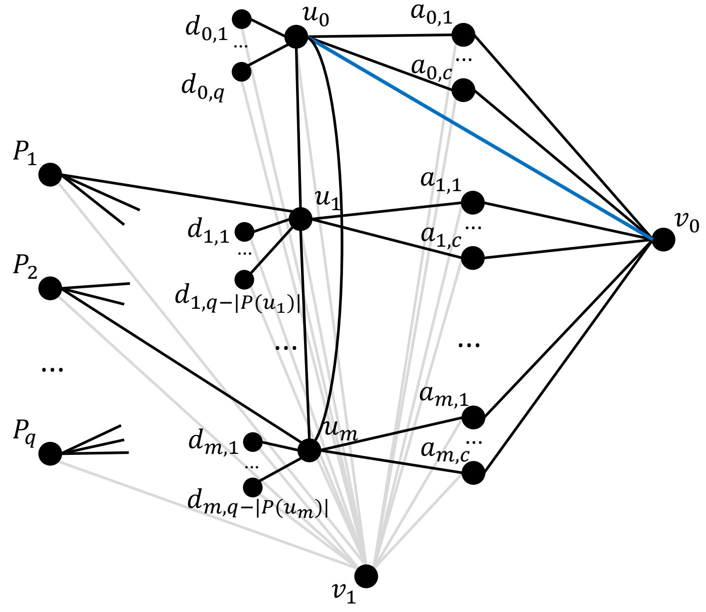


Figure S1: An illustration of the $\Gamma(c, P)$ network. Edges connecting $v_1$ with other nodes are grayed out to improve readability. The blue non-edge, $(u_0, v_0)$, is the one to be hidden.

An illustration of the $\Gamma(c, P)$ network is provided in Figure S1. Now, suppose that we want to hide a particular non-edge in this network, which is $(u_0, v_0)$. Suppose further that, in order to hide $(u_0, v_0)$, we were only allowed to add edges of the form $(P_i, v_0)$. Then, for any given similarity index, $s \in \mathcal{S}$, we need to understand how the addition of those $(P_i, v_0)$ edges affects the position of $s(u_0, v_0)$ in the similarity-based ranking of all non-edges; if the position of $s(u_0, v_0)$ decreases in this ranking, then $(u_0, v_0)$ becomes more hidden. The following lemma implies that for every similarity index in $\mathcal{S}$ there exists some constant, $c \in \mathbb{N}$, such that the position of $s(u_0, v_0)$ decreases if we add edges of the form $(P_i, v_0)$ to the network $\Gamma(c, P)$.

**Lemma 1.** *Consider a network $(V, E) = \Gamma(c, P)$ for which $m \geq 5$ and $|P_i| = 3$ for all $P_i \in P$. Furthermore, let $\widehat{A} = \{(P_i, v_0) : P_i \in P\}$, and for every $A \subseteq \widehat{A}$ let $P_A = \{P_i \in P : (P_i, v_0) \in A\}$, and let $P_A(u_j) = \{P_i \in P_A : u_j \in P_i\}$. Then, for every $A \subseteq \widehat{A}$, and every similarity index, $s \in \mathcal{S}$, there exists some constant, $c \in \mathbb{N}$, such that:*

*(a) for every non-edge of the form $(u_i, v_0) : i \in \{0, \dots, m\}$, we have:*

- $s(u_i, v_0) = s(u_0, v_0)$ *in the network $(V, E)$.*
- $s(u_i, v_0) = s(u_0, v_0)$ *in the network $(V, E \cup A)$ if $P_A(u_i) = \emptyset$.*
- $s(u_i, v_0) > s(u_0, v_0)$ *in the network $(V, E \cup A)$ if $P_A(u_i) \neq \emptyset$.*

*(b) for every non-edge of the form $(P_i, v_0) : i \in \{0, \dots, q\}$, we have:*

- $s(P_i, v_0) < s(u_0, v_0)$ *in the network $(V, E)$.*

- *$s(P_i, v_0) < s(u_0, v_0)$ in the network $(V, E \cup A)$ if $(P_i, v_0) \notin A$.*[2]

*(c) for every other non-edge, $e \in \bar{E} \setminus \{(u_0, v_0), \ldots, (u_m, v_0), (P_1, v_0), \ldots, (P_q, v_0)\}$:*

- *if $s(e) > s(u_0, v_0)$ in network $(V, E)$, then we also have $s(e) > s(u_0, v_0)$ in network $(V, E \cup A)$.*
- *if $s(e) = s(u_0, v_0)$ in network $(V, E)$, then we also have $s(e) = s(u_0, v_0)$ in network $(V, E \cup A)$.*
- *if $s(e) < s(u_0, v_0)$ in network $(V, E)$, then we also have $s(e) < s(u_0, v_0)$ in network $(V, E \cup A)$.*

Before we prove the correctness of Lemma 1, let us first provide an example. Suppose that $U = \{u_1, \ldots, u_7\}$, and $P = \{P_1, P_2, P_3\}$ where $P_1 = \{u_1, u_2, u_3\}$, $P_2 = \{u_3, u_4, u_5\}$ and $P_3 = \{u_5, u_6, u_7\}$. Then:

- The set $\widehat{A}$ consist of every edge of the form $(P_i, v_0)$. That is, $\widehat{A} = \{(P_1, v_0), (P_2, v_0), (P_3, v_0)\}$. Note that none of the edges in $\widehat{A}$ appear in the network $(V, E) = \Gamma(c, P)$.

- The set $A$ is a subset of $\widehat{A}$. Suppose that $A = \{(P_2, v_0), (P_3, v_0)\}$. Then:
  - $P_A$ consists of every $P_i$ that appears in $A$, i.e., $P_A = \{P_2, P_3\}$;
  - $P_A(u_i)$ consists of every $P_i$ that contains $u_i$ and appears in $P_A$. For instance, we have: $P_A(u_1) = \emptyset$ and $P_A(u_5) = \{P_2, P_3\}$;

- Using Lemma 1, we can analyse how the similarity of the non-edges in $\Gamma(c, P) = (V, E)$ would change if we add the edges $(P_2, v_0)$ and $(P_3, v_0)$. To this end, we simply set $A = \{(P_2, v_0), (P_3, v_0)\}$ and analyse the network $(V, E \cup A)$. Now, based on Lemma 1, we know that for every similarity index $s \in \mathcal{S}$ there exists some constant, $c \in \mathbb{N}$, such that:
  - Based on point (a) of Lemma 1:
    - for $i \in \{1, 2\}$ we have $s(u_i, v_0) = s(u_0, v_0)$ in $(V, E)$ and $s(u_i, v_0) = s(u_0, v_0)$ in $(V, E \cup A)$, because $P_A(u_i) = \emptyset$.
    - for $i \in \{3, \ldots, 7\}$ we have $s(u_i, v_0) = s(u_0, v_0)$ in $(V, E)$ and $s(u_i, v_0) > s(u_0, v_0)$ in $(V, E \cup A)$, because $P_A(u_i) \neq \emptyset$.
  - Based on point (b) of Lemma 1, we have: $s(P_1, v_0) < s(u_0, v_0)$ both in $(V, E)$ and in $(V, E \cup A)$, since $(P_1, v_0) \notin A$.
  - Based on point (c) of Lemma 1, for every non-edge $e$ whose form is neither $(u_i, v_0)$ nor $(P_i, v_0)$:
    - if we had $s(e) > s(u_0, v_0)$ in $(V, E)$, then we will also have $s(e) > s(u_0, v_0)$ in $(V, E \cup A)$.
    - if we had $s(e) = s(u_0, v_0)$ in $(V, E)$, then we will also have $s(e) = s(u_0, v_0)$ in $(V, E \cup A)$.
    - if we had $s(e) < s(u_0, v_0)$ in $(V, E)$, then we will also have $s(e) < s(u_0, v_0)$ in $(V, E \cup A)$.

Thus, before the addition of $\{(P_2, v_0), (P_3, v_0)\}$, the position of $(u_0, v_0)$ in the similarity-based ranking was the same as that of any non-edge of the form $(u_i, v_0)$. However, after the addition of $\{(P_2, v_0), (P_3, v_0)\}$, the edge $(u_0, v_0)$ has a ranking lower than that of any $(u_i, v_0) : i \in \{3, \ldots, 7\}$; as for the remaining non-edges, their relative rankings compared to that of $(u_0, v_0)$ remain unchanged after the addition of $\{(P_2, v_0), (P_3, v_0)\}$. Based on this, by adding $\{(P_2, v_0), (P_3, v_0)\}$ to the network $\Gamma(c, P) = (V, E)$, we decrease the position of $s(u_0, v_0)$ in the similarity-based ranking of all non-edges, i.e., we make $(u_0, v_0)$ more hidden.

Having explained Lemma 1 through an example, we will now prove the correctness of this lemma, before presenting our main theorem.

*Proof.* First, note that the following holds:

- for every $P_i$ and every network $(V, E \cup A) : A \subseteq \widehat{A}$ where $(P_i, v_0) \notin A$, we have $N(P_i, v_0) = \emptyset$, i.e., $P_i$ and $v_0$ have no common neighbours;

[2]Otherwise, if $(P_i, v_0) \in A$, then $(P_i, v_0)$ will not be a non-edge in $(V, E \cup A)$, and therefore we cannot compute $s(P_i, v_0)$.

- for every $d_{i,j}$ and every network $(V, E \cup A) : A \subseteq \widehat{A}$, we have $N(v_0, d_{i,j}) = \emptyset$.

This implies that for every similarity index, $s \in \mathcal{S}$, we have:

- for every $P_i$ and every network $(V, E \cup A) : A \subseteq \widehat{A}$ where $(P_i, v_0) \notin A$, we have $s(P_i, v_0) = 0$;
- for every $d_{i,j}$ and every network $(V, E \cup A) : A \subseteq \widehat{A}$, we have $s(v_0, d_{i,j}) = 0$.

One can also verify that for every $s \in \mathcal{S}$ and every network $(V, E \cup A) : A \subseteq \widehat{A}$ it holds that $s(u_0, v_0) > 0$. This implies that point (b) of Lemma 1 holds, and that point (c) holds for every non-edge of the form $(v_0, d_{i,j})$. We still need to prove the correctness of point (a), as well as the correctness of point (c) for every non-edge of the form:

(i) $(v_0, v_1)$

(ii) $(u_i, P_j)$ for $u_i \notin P_j$

(iii) $(u_i, a_{j,l})$ for $i \neq j$

(iv) $(u_i, d_{j,l})$ for $i \neq j$

(v) $(P_i, P_j)$ for $i \neq j$

(vi) $(P_i, a_{j,l})$

(vii) $(P_i, d_{j,l})$

(viii) $(a_{i_1,j_1}, a_{i_2,j_2})$

(ix) $(a_{i_1,j_1}, d_{i_2,j_2})$

(x) $(d_{i_1,j_1}, d_{i_2,j_2})$

Next, for every similarity index in $\mathcal{S}$, we will prove the correctness of point (a), as well as the correctness of point (c) for each of the above types of non-edges. To this end, first note that the following holds for every network $(V, E \cup A) : A \subseteq \widehat{A}$ and every $a_{i,j}$, $d_{i,j}$, $P_i$ in that network:

- $d(a_{i,j}) = 3$ (because $a_{i,j}$ is connected to $v_0$, $v_1$ and $u_i$);
- $d(d_{i,j}) = 2$ (because $d_{i,j}$ is connected to $v_1$ and $u_i$);
- $4 \leq d(P_i) \leq 5$ (because $P_i$ is connected to $v_1$ and to every $u_j \in P_i$, where we assumed that $|P_i| = 3$; also, if $P_i \in A$, then $P_i$ is connected to $v_0$).

Also note that $u_0 \notin P_i$ for every $P_i \in P$. Therefore, for any given $A \subseteq \widehat{A}$, we have: $P_A(u_0) = \emptyset$. In what follows, we will use the aforementioned facts without referring back to them. We will also use $r$ to denote the number of $a_{i,j}$ nodes, i.e., $r = c(m+1)$, and use $h$ to denote the number of $d_{i,j}$ nodes, i.e., $h = (m+1)q - 3q = mq - 2q$.

**Common Neighbours ($s^{\mathrm{CN}}$):** We choose $c = 6$. Then, to prove the correctness of point (a), it suffices to note that for every network $(V, E \cup A) : A \subseteq \widehat{A}$ we have:

$$\forall_{u_j \in \{u_0, \ldots, u_m\}} s^{\mathrm{CN}}(u_j, v_0) = 6 + |P_A(u_j)|.$$

Moving on to point (c), note that for every network $(V, E \cup A) : A \subseteq \widehat{A}$ we have $s^{\mathrm{CN}}(u_0, v_0) = 6$ and that the following holds:

(i) $s^{\mathrm{CN}}(v_0, v_1) = 6(m+1) + |A| > s^{\mathrm{CN}}(u_0, v_0)$, because the common neighbours of $u_0$ and $v_1$ are all the nodes $a_{i,j}$ and all the nodes $P_i$ where $(P_i, v_0) \in A$.

(ii) $s^{\mathrm{CN}}(u_i, P_j) \leq 4 < s^{\mathrm{CN}}(u_0, v_0)$, because the common neighbours of $u_i$ and $P_j$ consist of $v_1$ and every $u_l \in P_j : l \neq i$ (note that we assumed that $|P_j| = 3$, and $u_i$ may or may not be an element of $P_j$).

(iii) $s^{\mathrm{CN}}(u_i, a_{j,l}) = 2 < s^{\mathrm{CN}}(u_0, v_0)$, because the common neighbours of $u_i$ and $a_{j,l}$ are $v_1$ and $u_j$.

(iv) $s^{\mathrm{CN}}(u_i, d_{j,l}) = 2 < s^{\mathrm{CN}}(u_0, v_0)$, because the common neighbours of $u_i$ and $d_{j,l}$ are $v_1$ and $u_j$.

(v) $s^{\mathrm{CN}}(P_i, P_j) \leq 5 < s^{\mathrm{CN}}(u_0, v_0)$, because the common neighbours of $P_i$ and $P_j$ consist of $v_1$, and possibly $v_0$ (if $\{(P_i, v_0), (P_j, v_0)\} \subseteq A$), as well as the every element in $P_i \cap P_j$ (there can be at most 3 such elements, since we assumed that $|P_i| = |P_j| = 3$, and we place no restrictions on having $P_i = P_j$).

(vi) $s^{\mathrm{CN}}(P_i, a_{j,l}) \leq 3 < s^{\mathrm{CN}}(u_0, v_0)$, because the common neighbours of $P_i$ and $a_{j,l}$ consist of $v_1$, and possibly $v_0$ (if $(P_i, v_0) \in A$) and possibly $u_j$ (if $i = j$).

(vii) $s^{\mathrm{CN}}(P_i, d_{j,l}) \leq 2 < s^{\mathrm{CN}}(u_0, v_0)$, because the common neighbours of $P_i$ and $d_{j,l}$ consist of $v_1$ and possibly $u_j$ (if $i = j$).

(viii) $s^{\mathrm{CN}}(a_{i_1,j_1}, a_{i_2,j_2}) \leq 3 < s^{\mathrm{CN}}(u_0, v_0)$, because the common neighbours of $a_{i_1,j_1}$ and $a_{i_2,j_2}$ consist of $v_1$ and $v_0$ and possibly $u_{i_1}$ (if $i_2 = i_1$).

(ix) $s^{\mathrm{CN}}(a_{i_1,j_1}, d_{i_2,j_2}) \leq 2 < s^{\mathrm{CN}}(u_0, v_0)$, because the common neighbours of $a_{i_1,j_1}$ and $d_{i_2,j_2}$ consist of $v_1$ and possibly $u_{i_1}$ (if $i_2 = i_1$).

(x) $s^{\mathrm{CN}}(d_{i_1,j_1}, d_{i_2,j_2}) \leq 2 < s^{\mathrm{CN}}(u_0, v_0)$, because the common neighbours of $d_{i_1,j_1}$ and $d_{i_2,j_2}$ consist of $v_1$ and possibly $u_{i_1}$ (if $i_2 = i_1$).

**Salton similarity index ($s^{\mathrm{Sal}}$):** We choose $c = 1$. Then, to prove the correctness of point (a), it suffices to note that for every network $(V, E \cup A) : A \subseteq \widehat{A}$ we have:

$$s^{\mathrm{Sal}}(u_j, v_0) = \frac{1 + |P_A(u_j)|}{\sqrt{(r + |A|)(m + q + 2)}}, \quad \forall u_j \in \{u_0, \dots, u_m\}.$$

Moving on to point (c), note that $s^{\mathrm{Sal}}(u_0, v_0) \leq \frac{1}{\sqrt{42}}$ (since $m \geq 5$), and that the following holds for every $(V, E \cup A) : A \subseteq \widehat{A}$:

(i) $s^{\mathrm{Sal}}(v_0, v_1) = \frac{\sqrt{r+|A|}}{\sqrt{r+h+q+m+1}} > s^{\mathrm{Sal}}(u_0, v_0)$

(ii) $s^{\mathrm{Sal}}(u_i, P_j) \geq \frac{3}{\sqrt{(m+q+2)5}} > s^{\mathrm{Sal}}(u_0, v_0)$

(iii) $s^{\mathrm{Sal}}(u_i, a_{j,l}) \geq \frac{1}{\sqrt{(m+q+2)3}} > s^{\mathrm{Sal}}(u_0, v_0)$

(iv) $s^{\mathrm{Sal}}(u_i, d_{j,l}) \geq \frac{1}{\sqrt{(m+q+2)2}} > s^{\mathrm{Sal}}(u_0, v_0)$

(v) $s^{\mathrm{Sal}}(P_i, P_j) \geq \frac{1}{\sqrt{20}} > s^{\mathrm{Sal}}(u_0, v_0)$

(vi) $s^{\mathrm{Sal}}(P_i, a_{j,l}) \geq \frac{1}{2\sqrt{3}} > s^{\mathrm{Sal}}(u_0, v_0)$

(vii) $s^{\mathrm{Sal}}(P_i, d_{j,l}) \geq \frac{1}{\sqrt{10}} > s^{\mathrm{Sal}}(u_0, v_0)$

(viii) $s^{\mathrm{Sal}}(a_{i_1,j_1}, a_{i_2,j_2}) \geq \frac{2}{3} > s^{\mathrm{Sal}}(u_0, v_0)$

(ix) $s^{\mathrm{Sal}}(a_{i_1,j_1}, d_{i_2,j_2}) \geq \frac{1}{\sqrt{6}} > s^{\mathrm{Sal}}(u_0, v_0)$

(x) $s^{\mathrm{Sal}}(d_{i_1,j_1}, d_{i_2,j_2}) \geq \frac{1}{2} > s^{\mathrm{Sal}}(u_0, v_0)$

**Jaccard similarity index ($s^{\mathrm{Jac}}$):** We choose $c = 1$. Then, to prove the correctness of point (a), it suffices to note that for every $(V, E \cup A) : A \subseteq \widehat{A}$ we have:

$$s^{\mathrm{Jac}}(u_j, v_0) = \frac{1 + |P_A(u_j)|}{r + q + m + |A| + 2 - |P_A(u_j)|}, \quad \forall u_j \in \{u_0, \ldots, u_m\}.$$

Moving on to point (c), note that $s^{\mathrm{Jac}}(u_0, v_0) \leq \frac{1}{13}$ (since $m \geq 5$), and that the following holds for every $(V, E \cup A) : A \subseteq \widehat{A}$:

(i) $s^{\mathrm{Jac}}(v_0, v_1) = \frac{r+|A|}{r+h+q+m+1} > s^{\mathrm{Jac}}(u_0, v_0)$

(ii) $s^{\mathrm{Jac}}(u_i, P_j) \geq \frac{3}{m+q+3} > s^{\mathrm{Jac}}(u_0, v_0)$

(iii) $s^{\mathrm{Jac}}(u_i, a_{j,l}) \geq \frac{1}{m+q+3} > s^{\mathrm{Jac}}(u_0, v_0)$

(iv) $s^{\mathrm{Jac}}(u_i, d_{j,l}) \geq \frac{1}{m+q+2} > s^{\mathrm{Jac}}(u_0, v_0)$

(v) $s^{\mathrm{Jac}}(P_i, P_j) \geq \frac{1}{8} > s^{\mathrm{Jac}}(u_0, v_0)$

(vi) $s^{\mathrm{Jac}}(P_i, a_{j,l}) \geq \frac{1}{6} > s^{\mathrm{Jac}}(u_0, v_0)$

(vii) $s^{\mathrm{Jac}}(P_i, d_{j,l}) \geq \frac{1}{6} > s^{\mathrm{Jac}}(u_0, v_0)$

(viii) $s^{\mathrm{Jac}}(a_{i_1,j_1}, a_{i_2,j_2}) \geq \frac{2}{4} > s^{\mathrm{Jac}}(u_0, v_0)$

(ix) $s^{\mathrm{Jac}}(a_{i_1,j_1}, d_{i_2,j_2}) \geq \frac{1}{4} > s^{\mathrm{Jac}}(u_0, v_0)$

(x) $s^{\mathrm{Jac}}(d_{i_1,j_1}, d_{i_2,j_2}) \geq \frac{1}{3} > s^{\mathrm{Jac}}(u_0, v_0)$

**Sørensen similarity index ($s^{\mathrm{Sør}}$):** We choose $c = 1$. Then, to prove the correctness of point (a), it suffices to note that for every $(V, E \cup A) : A \subseteq \widehat{A}$ we have:

$$s^{\mathrm{Sør}}(u_j, v_0) = \frac{2 + 2|P_A(u_j)|}{r + q + m + |A| + 2}, \quad \forall u_j \in \{u_0, \ldots, u_m\}.$$

Moving on to point (c), note that $s^{\mathrm{Sør}}(u_0, v_0) \leq \frac{2}{13}$ (since $m \geq 5$), and that the following holds for every $(V, E \cup A) : A \subseteq \widehat{A}$:

(i) $s^{\mathrm{Sør}}(v_0, v_1) = \frac{2r+2|A|}{2r+h+q+m+1+|A|} > s^{\mathrm{Sør}}(u_0, v_0)$

(ii) $s^{\mathrm{Sør}}(u_i, P_j) \geq \frac{6}{m+q+7} > s^{\mathrm{Sør}}(u_0, v_0)$

(iii) $s^{\mathrm{Sør}}(u_i, a_{j,l}) \geq \frac{2}{m+q+5} > s^{\mathrm{Sør}}(u_0, v_0)$

(iv) $s^{\mathrm{Sør}}(u_i, d_{j,l}) \geq \frac{2}{m+q+4} > s^{\mathrm{Sør}}(u_0, v_0)$

(v) $s^{\mathrm{Sør}}(P_i, P_j) \geq \frac{2}{9} > s^{\mathrm{Sør}}(u_0, v_0)$

(vi) $s^{\mathrm{Sør}}(P_i, a_{j,l}) \geq \frac{2}{8} > s^{\mathrm{Sør}}(u_0, v_0)$

(vii) $s^{\mathrm{Sør}}(P_i, d_{j,l}) \geq \frac{2}{7} > s^{\mathrm{Sør}}(u_0, v_0)$

(viii) $s^{\text{Sør}}(a_{i_1,j_1}, a_{i_2,j_2}) \geq \frac{4}{6} > s^{\text{Sør}}(u_0, v_0)$

(ix) $s^{\text{Sør}}(a_{i_1,j_1}, d_{i_2,j_2}) \geq \frac{2}{5} > s^{\text{Sør}}(u_0, v_0)$

(x) $s^{\text{Sør}}(d_{i_1,j_1}, d_{i_2,j_2}) \geq \frac{2}{4} > s^{\text{Sør}}(u_0, v_0)$

**Hub Promoted similarity index ($s^{\text{HPI}}$):** We choose $c = 1$. Then, to prove the correctness of point (a), it suffices to note that for every $(V, E \cup A) : A \subseteq \widehat{A}$ we have:

$$s^{\text{HPI}}(u_j, v_0) = \frac{1 + |P_A(u_j)|}{r + |A|}, \quad \forall u_j \in \{u_0, \ldots, u_m\}.$$

Moving on to point (c), note that $s^{\text{HPI}}(u_0, v_0) \leq \frac{1}{6}$ (since $m \geq 5$), and that the following holds for every $(V, E \cup A) : A \subseteq \widehat{A}$:

(i) $s^{\text{HPI}}(v_0, v_1) = \frac{r+|A|}{r+|A|} = 1 > s^{\text{HPI}}(u_0, v_0)$

(ii) $s^{\text{HPI}}(u_i, P_j) \geq \frac{3}{5} > s^{\text{HPI}}(u_0, v_0)$

(iii) $s^{\text{HPI}}(u_i, a_{j,l}) \geq \frac{1}{3} > s^{\text{HPI}}(u_0, v_0)$

(iv) $s^{\text{HPI}}(u_i, d_{j,l}) \geq \frac{1}{2} > s^{\text{HPI}}(u_0, v_0)$

(v) $s^{\text{HPI}}(P_i, P_j) \geq \frac{1}{4} > s^{\text{HPI}}(u_0, v_0)$

(vi) $s^{\text{HPI}}(P_i, a_{j,l}) \geq \frac{1}{3} > s^{\text{HPI}}(u_0, v_0)$

(vii) $s^{\text{HPI}}(P_i, d_{j,l}) \geq \frac{1}{2} > s^{\text{HPI}}(u_0, v_0)$

(viii) $s^{\text{HPI}}(a_{i_1,j_1}, a_{i_2,j_2}) \geq \frac{2}{3} > s^{\text{HPI}}(u_0, v_0)$

(ix) $s^{\text{HPI}}(a_{i_1,j_1}, d_{i_2,j_2}) \geq \frac{1}{2} > s^{\text{HPI}}(u_0, v_0)$

(x) $s^{\text{HPI}}(d_{i_1,j_1}, d_{i_2,j_2}) \geq \frac{1}{2} > s^{\text{HPI}}(u_0, v_0)$

**Hub Depressed similarity index ($s^{\text{HDI}}$):** We choose $c = 1$. Then, to prove the correctness of point (a), it suffices to note that for every $(V, E \cup A) : A \subseteq \widehat{A}$ we have:

$$s^{\text{HDI}}(u_j, v_0) = \frac{1 + |P_A(u_j)|}{m + q + 2}, \quad \forall u_j \in \{u_0, \ldots, u_m\}.$$

Moving on to point (c), note that $s^{\text{HDI}}(u_0, v_0) \leq \frac{1}{7}$ (since $m \geq 5$), and that the following holds for every $(V, E \cup A) : A \subseteq \widehat{A}$:

(i) $s^{\text{HDI}}(v_0, v_1) = \frac{r+|A|}{r+h+q+m+1} > s^{\text{HDI}}(u_0, v_0)$

(ii) $s^{\text{HDI}}(u_i, P_j) \geq \frac{3}{m+q+2} > s^{\text{HDI}}(u_0, v_0)$

(iii) Either $s^{\text{HDI}}(u_i, a_{j,l}) = \frac{1}{m+q+2} = s^{\text{HDI}}(u_0, v_0)$ (if $i = j$) or $s^{\text{HDI}}(u_i, a_{j,l}) = \frac{2}{m+q+2} > s^{\text{HDI}}(u_0, v_0)$ (otherwise)

(iv) Either $s^{\text{HDI}}(u_i, d_{j,l}) = \frac{1}{m+q+2} = s^{\text{HDI}}(u_0, v_0)$ (if $i = j$) or $s^{\text{HDI}}(u_i, d_{j,l}) = \frac{2}{m+q+2} > s^{\text{HDI}}(u_0, v_0)$ (otherwise)

(v) $s^{\text{HDI}}(P_i, P_j) \geq \frac{1}{5} > s^{\text{HDI}}(u_0, v_0)$

(vi) $s^{\text{HDI}}(P_i, a_{j,l}) \geq \frac{1}{5} > s^{\text{HDI}}(u_0, v_0)$

(vii) $s^{\text{HDI}}(P_i, d_{j,l}) \geq \frac{1}{5} > s^{\text{HDI}}(u_0, v_0)$

(viii) $s^{\text{HDI}}(a_{i_1,j_1}, a_{i_2,j_2}) \geq \frac{2}{3} > s^{\text{HDI}}(u_0, v_0)$

(ix) $s^{\text{HDI}}(a_{i_1,j_1}, d_{i_2,j_2}) \geq \frac{1}{3} > s^{\text{HDI}}(u_0, v_0)$

(x) $s^{\text{HDI}}(d_{i_1,j_1}, d_{i_2,j_2}) \geq \frac{1}{2} > s^{\text{HDI}}(u_0, v_0)$

**Leicht-Holme-Newman similarity index ($s^{\text{LHN}}$):** We choose $c = 1$. Then, to prove the correctness of point (a), it suffices to note that for every $(V, E \cup A) : A \subseteq \widehat{A}$:

$$s^{\text{LHN}}(u_j, v_0) = \frac{1 + |P_A(u_j)|}{(r + |A|)(m + q + 2)}, \quad \forall u_j \in \{u_0, \dots, u_m\}.$$

Moving on to point (c), note that $s^{\text{LHN}}(u_0, v_0) \leq \frac{1}{42}$ (since $m \geq 5$), and that the following holds for every $(V, E \cup A) : A \subseteq \widehat{A}$:

(i) $s^{\text{LHN}}(v_0, v_1) = \frac{1}{r+h+q+m+1} > s^{\text{LHN}}(u_0, v_0)$

(ii) $s^{\text{LHN}}(u_i, P_j) \geq \frac{3}{(m+q+2)5} > s^{\text{LHN}}(u_0, v_0)$

(iii) $s^{\text{LHN}}(u_i, a_{j,l}) \geq \frac{1}{(m+q+2)3} > s^{\text{LHN}}(u_0, v_0)$

(iv) $s^{\text{LHN}}(u_i, d_{j,l}) \geq \frac{1}{(m+q+2)2} > s^{\text{LHN}}(u_0, v_0)$

(v) $s^{\text{LHN}}(P_i, P_j) \geq \frac{1}{20} > s^{\text{LHN}}(u_0, v_0)$

(vi) $s^{\text{LHN}}(P_i, a_{j,l}) \geq \frac{1}{12} > s^{\text{LHN}}(u_0, v_0)$

(vii) $s^{\text{LHN}}(P_i, d_{j,l}) \geq \frac{1}{10} > s^{\text{LHN}}(u_0, v_0)$

(viii) $s^{\text{LHN}}(a_{i_1,j_1}, a_{i_2,j_2}) \geq \frac{2}{9} > s^{\text{LHN}}(u_0, v_0)$

(ix) $s^{\text{LHN}}(a_{i_1,j_1}, d_{i_2,j_2}) \geq \frac{1}{6} > s^{\text{LHN}}(u_0, v_0)$

(x) $s^{\text{LHN}}(d_{i_1,j_1}, d_{i_2,j_2}) \geq \frac{1}{4} > s^{\text{LHN}}(u_0, v_0)$

**Adamic-Adar similarity index ($s^{\text{AA}}$):** We choose $c = 3$. Then, to prove the correctness of point (a), it suffices to note that for every $(V, E \cup A) : A \subseteq \widehat{A}$ we have:

$$s^{\text{AA}}(u_j, v_0) = \frac{3}{\log(3)} + \frac{|P_A(u_j)|}{\log(5)}, \quad \forall u_j \in \{u_0, \dots, u_m\}.$$

Moving on to point (c), note that $s^{\text{AA}}(u_0, v_0) = \frac{3}{\log(3)} > 6$ and that the following holds for every $(V, E \cup A) : A \subseteq \widehat{A}$:

(i) $s^{\text{AA}}(v_0, v_1) = \frac{r}{\log(3)} + \frac{|A|}{\log(5)} > s^{\text{AA}}(u_0, v_0)$

(ii) $s^{\text{AA}}(u_i, P_j) = \frac{1}{\log(r+h+q+m+1)} + \frac{3}{\log(q+m+4)} < s^{\text{AA}}(u_0, v_0)$

(iii) $s^{\text{AA}}(u_i, a_{j,l}) \leq \frac{1}{\log(r+h+q+m+1)} + \frac{1}{\log(q+m+4)} < s^{\text{AA}}(u_0, v_0)$

(iv) $s^{\text{AA}}(u_i, d_{j,l}) \leq \frac{1}{\log(r+h+q+m+1)} + \frac{1}{\log(q+m+4)} < s^{\text{AA}}(u_0, v_0)$

(v) $s^{\text{AA}}(P_i, P_j) \leq \frac{1}{\log(r+h+q+m+1)} + \frac{1}{\log(r+|A|)} + \frac{3}{\log(q+m+4)} < s^{\text{AA}}(u_0, v_0)$

(vi) $s^{\text{AA}}(P_i, a_{j,l}) \leq \frac{1}{\log(r+h+q+m+1)} + \frac{1}{\log(r+|A|)} + \frac{1}{\log(q+m+4)} < s^{\text{AA}}(u_0, v_0)$

(vii) $s^{\mathrm{AA}}(P_i, d_{j,l}) \leq \frac{1}{\log(r+h+q+m+1)} + \frac{1}{\log(q+m+4)} < s^{\mathrm{AA}}(u_0, v_0)$

(viii) $s^{\mathrm{AA}}(a_{i_1,j_1}, a_{i_2,j_2}) \leq \frac{1}{\log(r+h+q+m+1)} + \frac{1}{\log(r+|A|)} + \frac{1}{\log(q+m+4)} < s^{\mathrm{AA}}(u_0, v_0)$

(ix) $s^{\mathrm{AA}}(a_{i_1,j_1}, d_{i_2,j_2}) \leq \frac{1}{\log(r+h+q+m+1)} + \frac{1}{\log(q+m+4)} < s^{\mathrm{AA}}(u_0, v_0)$

(x) $s^{\mathrm{AA}}(d_{i_1,j_1}, d_{i_2,j_2}) \leq \frac{1}{\log(r+h+q+m+1)} + \frac{1}{\log(q+m+4)} < s^{\mathrm{AA}}(u_0, v_0)$

**Resource Allocation similarity index ($s^{\mathrm{RA}}$):** We choose $c = 3$. Then, to prove the correctness of point (a), it suffices to note that for every $(V, E \cup A) : A \subseteq \widehat{A}$ we have:

$$s^{\mathrm{RA}}(u_j, v_0) = \frac{3}{3} + \frac{|P_A(u_j)|}{5}$$

Moving on to point (c), note that $s^{\mathrm{RA}}(u_0, v_0) = 1$ and that the following holds for every $(V, E \cup A) : A \subseteq \widehat{A}$:

(i) $s^{\mathrm{RA}}(v_0, v_1) = \frac{r}{3} + \frac{|A|}{5} > s^{\mathrm{RA}}(u_0, v_0)$

(ii) $s^{\mathrm{RA}}(u_i, P_j) = \frac{1}{r+h+q+m+1} + \frac{3}{q+m+4} < s^{\mathrm{RA}}(u_0, v_0)$

(iii) $s^{\mathrm{RA}}(u_i, a_{j,l}) \leq \frac{1}{r+h+q+m+1} + \frac{1}{q+m+4} < s^{\mathrm{RA}}(u_0, v_0)$

(iv) $s^{\mathrm{RA}}(u_i, d_{j,l}) \leq \frac{1}{r+h+q+m+1} + \frac{1}{q+m+4} < s^{\mathrm{RA}}(u_0, v_0)$

(v) $s^{\mathrm{RA}}(P_i, P_j) \leq \frac{1}{r+h+q+m+1} + \frac{1}{r+|A|} + \frac{3}{q+m+4} < s^{\mathrm{RA}}(u_0, v_0)$

(vi) $s^{\mathrm{RA}}(P_i, a_{j,l}) \leq \frac{1}{r+h+q+m+1} + \frac{1}{r+|A|} + \frac{1}{q+m+4} < s^{\mathrm{RA}}(u_0, v_0)$

(vii) $s^{\mathrm{RA}}(P_i, d_{j,l}) \leq \frac{1}{r+h+q+m+1} + \frac{1}{q+m+4} < s^{\mathrm{RA}}(u_0, v_0)$

(viii) $s^{\mathrm{RA}}(a_{i_1,j_1}, a_{i_2,j_2}) \leq \frac{1}{r+h+q+m+1} + \frac{1}{r+|A|} + \frac{1}{q+m+4} < s^{\mathrm{RA}}(u_0, v_0)$

(ix) $s^{\mathrm{RA}}(a_{i_1,j_1}, d_{i_2,j_2}) \leq \frac{1}{r+h+q+m+1} + \frac{1}{q+m+4} < s^{\mathrm{RA}}(u_0, v_0)$

(x) $s^{\mathrm{RA}}(d_{i_1,j_1}, d_{i_2,j_2}) \leq \frac{1}{r+h+q+m+1} + \frac{1}{q+m+4} < s^{\mathrm{RA}}(u_0, v_0)$

This concludes the proof of Lemma 1. □

Having defined the $\Gamma(c, P)$ network, and having proven the correctness of Lemma 1, we are now ready to present our main theorem.

**Theorem 1.** *The problem of Evading Link Prediction is NP-complete for each of the following similarity indices: Common Neighbours [24], Salton [26], Jaccard [13], Sørensen [28], Hub Promoted [25], Hub Depressed [25], Leicht-Holme-Newman [19], Adamic-Adar [1] and Resource Allocation [33].*

Before we present our proof, let us first explain the intuition behind it. Specifically, the proof is based on a reduction from the NP-complete *3-Set Cover problem* to a particular instance of our problem of *Evading Link Prediction*. Recall that the 3-Set Cover problem is defined by (i) a universe $U = \{u_1, \ldots, u_l\}$; (ii) a collection of subsets $P = \{P_1, \ldots, P_m\}$ such that $\forall_j P_j \subset U$ and $\forall_j |P_j| = 3$; and (iii) an integer $k \leq m$. The goal is then to determine whether there exist $k$ elements of $P$ the union of which equals $U$. In our proof, the 3-Set Cover problem will be reduced to the problem of *Evading Link Prediction* (see Definition 1) where:

- the network under consideration is: $G = \Gamma(c, P)$, where $c \in \mathbb{N}$ satisfies the conditions in Lemma 1;
- the set of non-edges to be hidden is: $H = \{(u_0, v_0)\}$;
- the set of edges that can be added is: $\widehat{A} = \{(P_i, v_0) : P_i \in P\}$;

- the set of edges that can be removed is: $\widehat{R} = \emptyset$;
- the budget that specifies the number of edges that can be modified (i.e., added or removed) is: $b = k$.

Note that the above instance of the problem of Evading Link Prediction is exactly the same as the instance considered in Lemma 1. We already know from this lemma that, in the similarity-based ranking of all non-edges in $\Gamma(c, P)$, the position of $(u_0, v_0)$ is the same as that of any non-edge of the form $(u_i, v_0)$. However, after adding some edges, $A \subseteq \widehat{A}$, the position of $(u_0, v_0)$ becomes lower than that of any $(u_i, v_0)$ such that $\exists P_j \in A : u_i \in P_j$. As for the remaining non-edges, their relative ranking compared to that of $(u_0, v_0)$ remains unchanged after the addition of $A$. Based on this, in order to decrease the position of $(u_0, v_0)$ in the similarity-based ranking as much as possible, we need to add some edges, $A \subseteq \widehat{A}$, such that: $\exists P_j \in A : u_i \in P_j$ for every $u_i \in U$. That is, we need to find a subset of $P$ that covers all the elements in $U$, which leads us to the 3-Set Cover problem.

*Proof.* The problem of Evading Link Prediction is trivially in NP, since computing $AUC$ and $AP$ before and after the addition of a given set of edges $A \subseteq \widehat{A}$ and the removal of a given set of edges $R \subseteq \widehat{R}$ can be done in polynomial time for every similarity index in $\mathcal{S}$.

Next, we will prove that the problem is NP-hard. To this end, we will give a reduction from the NP-complete 3-Set Cover problem. This problem is defined by (i) a universe $U = \{u_1, \dots, u_l\}$; (ii) a collection of subsets $P = \{P_1, \dots, P_m\}$ such that $\forall_j P_j \subset U$ and $\forall_j |P_j| = 3$; and (iii) an integer $k \leq m$. The goal is then to determine whether there exist $k$ elements of $P$ the union of which equals $U$.

Let us assume that $m \geq 5$, as all other cases can be easily solved in polynomial time. Now, for any given similarity index, $s \in \mathcal{S}$, consider the following instance of the problem of Evading Link Prediction $(G, s, f, H, b, \widehat{A}, \widehat{R})$, where:

- $G = (V, E) = \Gamma(c, P)$, where $c \in \mathbb{N}$ is chosen to be a constant that satisfies the conditions in Lemma 1 (the lemma states that such a constant exists);
- $s$ is the similarity index under consideration;
- $f$ is either the $AUC$ or the $AP$ metric;
- $H = \{(u_0, v_0)\}$;
- $b = k$, where $k$ is the parameter of the 3-Set Cover problem, and the goal is to determine whether there exist $k$ elements of $P$ the union of which equals $U$;
- $\widehat{A} = \{(P_i, v_0) : P_i \in P\}$;
- $\widehat{R} = \emptyset$.

Let us also introduce the following notation:

- $\Upsilon^{<}(G) = \{e \in \bar{E} : s(e) < s(u_0, v_0)\}$ in network $G = (V, E)$;
- $\Upsilon^{=}(G) = \{e \in \bar{E} \setminus \{(u_0, v_0)\} : s(e) = s(u_0, v_0)\}$ in network $G = (V, E)$;
- $\Upsilon^{>}(G) = \{e \in \bar{E} : s(e) > s(u_0, v_0)\}$ in network $G = (V, E)$.

Note that $\bar{E}$ is the set of non-edges in $G = (V, E)$, whereas $\bar{E} \setminus A$ is the set of non-edges in $(V, E \cup A)$. For every network $G' = (V, E \cup A) : A \subseteq \widehat{A}$, we know from the definition of $AUC$ in Section S2 that:

$$AUC(E \cup A, H) = \frac{|\Upsilon^{<}(G')| + \frac{1}{2}|\Upsilon^{=}(G')|}{|\bar{E} \setminus A| - 1}. \tag{1}$$

We also know from the definition of $AP$ in Section S2 that:

$$AP(E \cup A, H) = \frac{1}{|\Upsilon^{>}(G')| + 1 + \frac{1}{2}|\Upsilon^{=}(G')|}. \tag{2}$$

Now, let $U_A = \{u_i : \exists_{P_j \in P_A} u_i \in P_j\}$. Point (b) of Lemma 1 implies that:

$$|\Upsilon^{<}(G')| = |\Upsilon^{<}(G)| - |A|. \tag{3}$$

On the other hand, point (a) of Lemma 1 implies that:

$$|\Upsilon^{=}(G')| = |\Upsilon^{=}(G)| - |U_A|, \tag{4}$$

$$|\Upsilon^{>}(G')| = |\Upsilon^{>}(G)| + |U_A|, \tag{5}$$

Equations (1), (3) and (4) imply that:

$$AUC(E \cup A, H) = \frac{|\Upsilon^{<}(G)| - |A| + \frac{1}{2}(|\Upsilon^{=}(G)| - |U_A|)}{|\bar{E}| - |A| - 1} \tag{6}$$

On the other hand, equations (2) and (5) imply that:

$$AP(E \cup A, H) = \frac{1}{|\Upsilon^{>}(G)| + |U_A| + 1 + \frac{1}{2}(|\Upsilon^{=}(G)| - |U_A|)} \tag{7}$$

This, in turn, implies that:

$$AP(E \cup A, H) = \frac{1}{|\Upsilon^{>}(G)| + 1 + \frac{1}{2}(|\Upsilon^{=}(G)| + |U_A|)}. \tag{8}$$

Equations (6) and (8) imply that both $AUC$ and $AP$ decrease with $|U_A|$. Thus, for each of these two metrics an optimal choice of $A$ is one that maximizes $|U_A|$. This happens when $U_A = U$. For any choice of $A$ such that $U_A = U$, the following holds: $\forall_{u_j \in U} \exists_{(P_i, v_0) \in A} u_j \in P_i$. Such an optimal choice of $A$ constitutes a solution to our instance of the problem of Evading Link Prediction. It also corresponds directly to a solution to the 3-Set Cover problem. □

## S3.2 Proof of NP-completeness Using Edge Removal

The proof of Theorem 1 utilized only the addition of edges. We will now prove that the problem of Evading Link Prediction is also NP-complete when the set of actions is restricted to edge removal. In particular, Theorem 2 deals with the Common Neighbors similarity index, while Theorem 3 deals with the remaining similarity indices.

**Theorem 2.** *The problem of Evading Link Prediction is NP-complete given the Common Neighbours [24] similarity index, even when the set of actions is limited to edge removal.*

*Proof.* The problem of Evading Link Prediction is trivially in NP, since computing $AUC$ and $AP$ before and after the removal of a given set of edges $R \subseteq \widehat{R}$ can be done in polynomial time for every similarity index.

Next, we will prove that the problem is NP-hard. To this end, we will give a reduction from the NP-complete Independent Set problem. This problem is defined by a network $G' = (V', E')$ and an integer $k \leq |V'|$. The goal is then to determine whether there exist $k$ different nodes in $G'$ without any edges between them. Based on network $G'$, we will construct network $G$ as follows (an example of this network is illustrated in Figure S2):

- **The set of nodes:** For every $v_i \in V'$ we create a node $v_i$, as well as $2n - d_{G'}(v_i)$ nodes, $a_{i,1}, \ldots, a_{2n-d_{G'}(v_i)}$. Additionally, we create nodes $v_0$, $w_0$ and $w_1$, as well as $2n$ nodes, $z_1, \ldots, z_{2n}$, and $2n-1$ nodes, $a_{0,1}, \ldots, a_{0,2n-1}$.
- **The set of edges:** We create an edge between two nodes, $v_i$ and $v_j$, if and only if this edge is present in network $G'$. For every node $v_i$, such that $i > 0$, we create the edge $(v_i, w_1)$. For every node $a_{i,j}$ we create the edge $(v_i, a_{i,j})$, as well as either edge $(w_0, a_{i,j})$ if $i = 0$, or edge $(w_1, a_{i,j})$ if $i > 0$. Finally, for every node $z_i$ we create edges $(z_i, v_1), \ldots, (z_i, v_n)$.

Now, consider the following instance of the problem of Evading Link Prediction $(G, s, f, H, b, \widehat{A}, \widehat{R})$, where:

- $G$ is the network we just constructed,

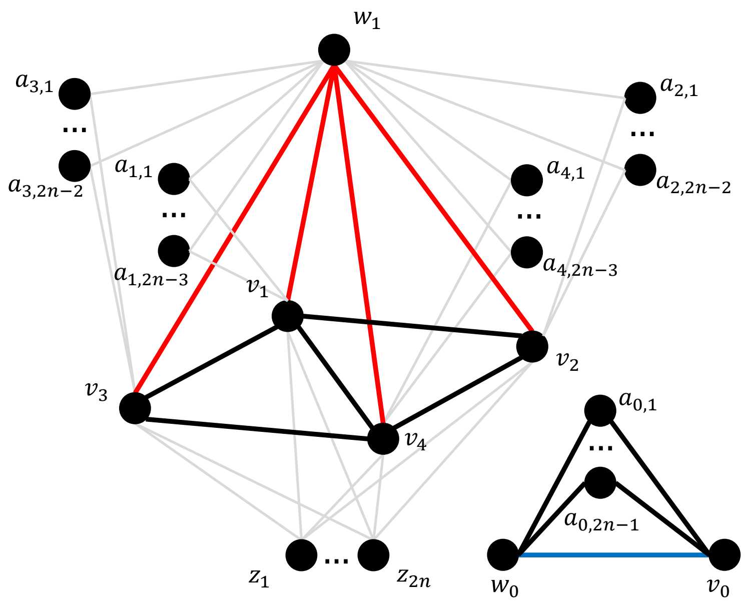


Figure S2: An example of network $G$, which is used in the proof of Theorem 2. The red edges are allowed to be removed, while the blue non-edge, $(v_0, w_0)$, is the one to be hidden.

- $s$ is the Common Neighbors similarity index, i.e., $s(v, w) = |N(v, w)|$,
- $f$ is either the $AUC$ or the $AP$ metric,
- $H = \{(v_0, w_0)\}$,
- $b = k$, where $k$ is the parameter of the Independent Set problem,
- $\widehat{A} = \emptyset$,
- $\widehat{R} = \{(v_1, w_1), \ldots, (v_n, w_1)\}$.

For any $R \subseteq \widehat{R}$ in network $(V, E \setminus R)$ we have:

- $|N(v_0, w_0)| = 2n - 1$,
- $|N(v_i, v_j)| \geq 2n > |N(v_0, w_0)|$,
- $|N(z_i, z_j)| = n < |N(v_0, w_0)|$,
- $|N(z_i, w_1)| \leq n < |N(v_0, w_0)|$,
- $|N(v_1, a_{j,l})| \leq 2 < |N(v_0, w_0)|$,
- $|N(a_{i_1,j_1}, a_{i_2,j_2})| \leq 2 < |N(v_0, w_0)|$,
- $|N(z_i, a_{j,l})| = 1 < |N(v_0, w_0)|$.

Every other pair of nodes has an empty set of common neighbors. Therefore, every non-edge $(v_i, v_j)$ has a greater value of the similarity index $s$ than $(v_0, w_0)$, while every other non-edge from outside $R$ has a smaller value of the similarity index $s$ than $(v_0, w_0)$. As for the value of $s$ for the non-edges $(v_i, w_1)$, this value is: $s(v_i, w_1) = 2n - r_i$, where $r_i$ is the number of $v_j \in N(v_i)$ such that $(v_j, w_1) \in R$. Since $s(v_0, w_0) = 2n - 1$, we have that $s(v_i, w_1) > s(v_0, w_0)$ if and only if $r_i = 0$, i.e., when $R$ does not contain any edges between $w_1$ and the neighbors of $v_i$. Now, let us introduce the following notation:

- $\Upsilon^{<}(R) = \{e \in \bar{E} \setminus R : s(e) < s(v_0, w_0)\}$ in network $(V, E \setminus R)$,
- $\Upsilon^{>}(R) = \{e \in \bar{E} \setminus R : s(e) > s(v_0, w_0)\}$ in network $(V, E \setminus R)$,
- $\Psi^{>}(R) = \{e \in R : s(e) > s(v_0, w_0)\}$ in network $(V, E \setminus R)$,
- $\Psi^{=}(R) = \{e \in R : s(e) = s(v_0, w_0)\}$ in network $(V, E \setminus R)$,
- $\Psi^{<}(R) = \{e \in R : s(e) < s(v_0, w_0)\}$ in network $(V, E \setminus R)$.

For every network $(V, E \setminus R) : R \subseteq \widehat{R}$, we have that:

$$AUC(E \setminus R, H) = \frac{|\Upsilon^{<}(R)| + |\Psi^{<}(R)| + \frac{1}{2}|\Psi^{=}(R)|}{|\bar{E}| + |R| - 1}.$$

Based on this, as well as the fact that $\Psi^{>}(R) + \Psi^{=}(R) + \Psi^{<}(R) = |R|$, we have that:

$$AUC(E \setminus R, H) = \frac{|\Upsilon^{<}(R)| + |R| - |\Psi^{>}(R)| - \frac{1}{2}|\Psi^{=}(R)|}{|\bar{E}| + |R| - 1}.$$

We also have that:

$$AP(E \setminus R, H) = \frac{1}{|\Upsilon^{>}(G')| + 1 + |\Psi^{>}(G')| + \frac{1}{2}|\Psi^{=}(G')|}.$$

We can see that both $AUC$ and $AP$ are minimized when $|\Psi^{>}(R)| = k$ (notice that since the removal budget is $b = k$, we cannot remove more than $k$ edges). However, we have shown that in order for $(v_i, w_1)$ to be part of $\Psi^{>}(R)$, the set $R$ cannot contain any edges between $w_1$ and the neighbors of $v_i$. Therefore, all $v_i$ such that $(v_i, w_1) \in \Psi^{>}(R)$ must form an independent set in the network induced by $V' \times V'$. Since $(v_i, v_j) \in E$ if and only if $(v_i, v_j) \in E'$, such nodes also form an independent set in $G'$, thus providing a solution to the given instance of the Independent Set problem. This concludes the proof. □

Having proven the NP-completeness for the Common Neighbors similarity index in Theorem 2, we now prove the NP-completeness for the remaining similarity indices.

**Lemma 2.** *Let $r_i = |\{(v_i, v_j) \in R\}|$, i.e., $r_i$ is the number of edges in $R$ incident to $v_i$. For any $V'$, any $\widehat{R} \subseteq V' \times V'$, any $|V'| \geq k > 3$, and any similarity index, $s \in \{s^{\text{Sal}}, s^{\text{Jac}}, s^{\text{Sør}}, s^{\text{HPI}}, s^{\text{HDI}}, s^{\text{LHN}}, s^{\text{AA}}, s^{\text{RA}}\}$, there exists a network $G = (V, E)$ with $h \in \bar{E}$ and $\bar{E}^* = \{e_1, \ldots, e_n\} \subseteq \bar{E}$, such that for every $R \subseteq \widehat{R}$ the following statements hold:*

*(a) for every non-edge $e_i \in \bar{E}^*$:*

- *if $r_i = k - 2$ then $s(e_i) = s(h)$ in the network $(V, E \setminus R)$,*
- *if $r_i > k - 2$ then $s(e_i) > s(h)$ in the network $(V, E \setminus R)$,*
- *if $r_i < k - 2$ then $s(e_i) < s(h)$ in the network $(V, E \setminus R)$.*

*(b) for every non-edge $e \in R$ we have that $s(e) > s(h)$ in the network $(V, E \setminus R)$.*

*(c) for every other non-edge, $e \in \bar{E} \setminus (\{h\} \cup \bar{E}^*)$ either:*

- *$s(e) > s(h)$ in the network $(V, E)$, and $s(e) > s(h)$ in the network $(V, E \setminus R)$,*
- *or $s(e) < s(h)$ in the network $(V, E)$, and $s(e) < s(h)$ in the network $(V, E \setminus R)$.*

*Proof.* We will first prove the lemma for indices in $\{s^{\text{Sal}}, s^{\text{Jac}}, s^{\text{Sør}}, s^{\text{HPI}}, s^{\text{HDI}}, s^{\text{LHN}}\}$. Let $G$ be a network created as follows (an example of this is illustrated in Figure S3):

- **The set of nodes:** For every $v_i \in \{v_0, \ldots, v_n\}$ we create three nodes, $v_i$, $w_i$, $a_i$, as well as $n^2$ nodes, $u_{i,1}, \ldots, u_{i,n^2}$. Additionally, we create $\gamma$ nodes, $z_1, \ldots, z_\gamma$, as well as $\gamma + n - k + 1$ nodes, $d_1, \ldots, d_{\gamma+n-k+1}$.

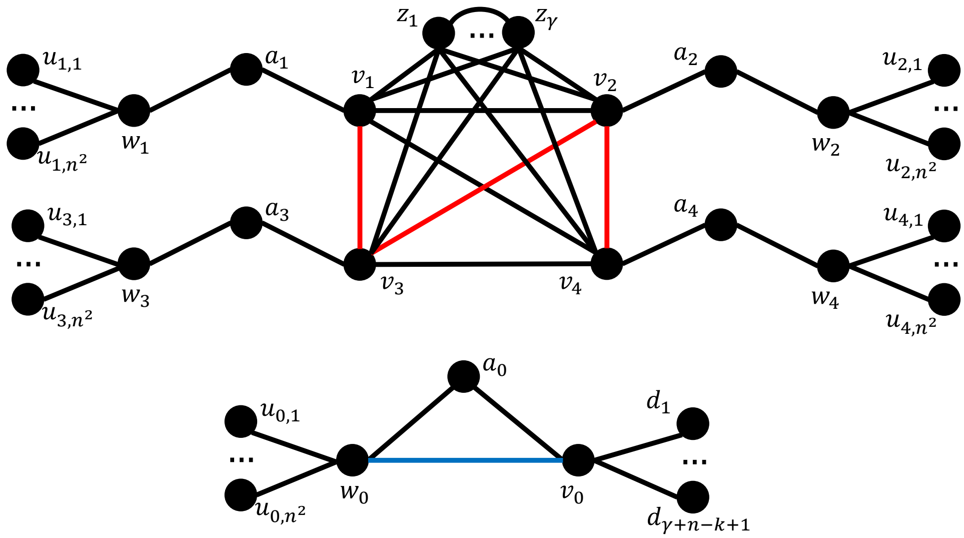


Figure S3: An illustration of the network used in the proof of Lemma 2 given a similarity index $s \in \{s^{\text{Sal}}, s^{\text{Jac}}, s^{\text{Sør}}, s^{\text{HPI}}, s^{\text{HDI}}, s^{\text{LHN}}\}$. The red edges are allowed to be removed, while the blue non-edge, $(v_0, w_0)$, is the one to be hidden.

- **The set of edges:** We connect nodes in $\{v_1, \dots, v_n, z_1, \dots, z_\gamma\}$ into a clique. For every node $a_i$ we create edges $(a_i, v_i)$ and $(a_i, w_i)$. For every node $u_{i,j}$ we create an edge $(u_{i,j}, w_i)$. Finally, for every node $d_i$ we create an edge $(d_i, v_0)$.

We will show that for any given similarity index, $s \in \{s^{\text{Sal}}, s^{\text{Jac}}, s^{\text{Sør}}, s^{\text{HPI}}, s^{\text{HDI}}, s^{\text{LHN}}\}$, there exists a $\gamma \in \mathbb{N}$ such that the network $G$, with $h = (v_0, w_0)$, and $\bar{E}^* = \{e_1, ..., e_n\} : e_i = (v_i, w_i)$, satisfies the lemma. Notice that the value $s(v_i, w_i)$ of every similarity index $s \in \{s^{\text{Sal}}, s^{\text{Jac}}, s^{\text{Sør}}, s^{\text{HPI}}, s^{\text{HDI}}, s^{\text{LHN}}\}$ depends only on the values of $d(v_i)$, $d(w_i)$ and $|N(v_i, w_i)|$ (it can only decrease with $d(v_i)$ and $d(w_i)$ and can only increase with $|N(v_i, w_i)|$). For every non-edge $(v_i, w_i)$ we have $|N(v_i, w_i)| = 1$ and $d(w_i) = n^2 + 1$. Hence, whether $s(v_i, w_i) > s(v_0, w_0)$ or $s(v_i, w_i) = s(v_0, w_0)$ or $s(v_i, w_i) < s(v_0, w_0)$ depends solely on the value of $d(v_i)$. We have that $d(v_0) = \gamma + n - k + 2$ and $d(v_i) = \gamma + n - r_i$ for $i > 0$. Therefore, $s(v_i, w_i) = s(v_0, w_0)$ when $d(v_0) = d(v_i)$, which is the case for $r_i = k - 2$. Since the value of $s(v_i, w_i)$ decreases with $r_i$, we have that $s(v_i, w_i) < s(v_0, w_0)$ when $r_i < k - 2$, and analogously $s(v_i, w_i) > s(v_0, w_0)$ when $r_i > k - 2$. This proves point (a) of the lemma.

Notice also that since we have $|N(v_0, w_0)| = 1$, then we also have $s(v_0, w_0) > 0$ for every similarity index $s \in \{s^{\text{Sal}}, s^{\text{Jac}}, s^{\text{Sør}}, s^{\text{HPI}}, s^{\text{HDI}}, s^{\text{LHN}}\}$. Therefore, for every non-edge $e$ where the ends do not have any common neighbors, we have that $s(e) < s(v_0, w_0)$ in both network $(V, E)$ and network $(V, E \setminus R)$ (notice that the removal of $R$ cannot increase the number of common neighbors for any pair of nodes), hence any such $e$ satisfies point (c) of the lemma.

Finally, notice that $|N(a_i, u_{i,j})| = |N(a_0, d_i)|$ and $d(u_{i,j}) = d(d_i)$, hence we only need to prove point (c) of the lemma for either $(a_i, u_{i,j})$ or $(a_0, d_i)$. The same is true for $(u_{i,j}, u_{i,l})$ and $(d_i, d_j)$. What remains is to give the value of $\gamma$ for every similarity index such that the following conditions are satisfied:

- for every $e \in R$ we have $s(e) > s(v_0, w_0)$ in the network $(V, E \setminus R)$ to prove point (b) of the lemma,
- Each of the following non-edges:
  (i) $(v_i, a_j)$,
  (ii) $(d_i, a_0)$,

(iii) $(d_i, d_j)$,

(iv) $(z_i, a_j)$.

either has always greater or always smaller value of $s$ than $(v_0, w_0)$ (to prove point (c) of the lemma). For all other non-edges either their ends have no common neighbors, or their similarity is the same as one of the non-edges we listed above.

In what follows, we will use the following facts:

- $|N(v_0, w_0)| = 1$,
- $|N(v_i, v_j)| \in [\gamma, \gamma + n - 2]$,
- $|N(v_i, a_j)| \leq 1$,
- $|N(d_i, a_0)| = 1$,
- $|N(d_i, d_j)| = 1$,
- $|N(z_l, a_j)| = 1$,
- $d(v_i) \in [\gamma + 1, \gamma + n]$,
- $d(w_i) = n^2 + 1$,
- $d(a_i) = 2$,
- $d(d_i) = 1$,
- $d(z_i) = n + \gamma - 1$.

Unless stated otherwise, we set $\gamma = 1$.

**Salton similarity index ($s^{\text{Sal}}$):** we have: $s^{\text{Sal}}(v_0, w_0) = \frac{|N(v_0,w_0)|}{\sqrt{d(v_0)d(w_0)}} = \frac{1}{\sqrt{d(v_0)(n^2+1)}}$, which gives us $s^{\text{Sal}}(v_0, w_0) \leq \frac{1}{\sqrt{2(n^2+1)}} < \frac{1}{\sqrt{2}n}$. We then have:

$$s^{\text{Sal}}(v_i, v_j) = \frac{|N(v_i, v_j)|}{\sqrt{d(v_i)d(v_j)}} \geq \frac{1}{n+1} > \frac{1}{\sqrt{2}n} > s^{\text{Sal}}(v_0, w_0),$$

which satisfies point (b) of the lemma. Now, to satisfy point (c) of the lemma we show the following:

(i) $s^{\text{Sal}}(v_i, a_j) = \frac{|N(v_i,a_j)|}{\sqrt{d(v_i)d(a_j)}} \geq \frac{1}{\sqrt{2(n+1)}} > \frac{1}{\sqrt{2}n} > s^{\text{Sal}}(v_0, w_0)$,

(ii) $s^{\text{Sal}}(d_i, a_0) = \frac{|N(d_i,a_0)|}{\sqrt{d(d_i)d(a_0)}} = \frac{1}{\sqrt{2}} > \frac{1}{\sqrt{2}n} > s^{\text{Sal}}(v_0, w_0)$,

(iii) $s^{\text{Sal}}(d_i, d_j) = \frac{|N(d_i,d_j)|}{\sqrt{d(d_i)d(d_j)}} = 1 > s^{\text{Sal}}(v_0, w_0)$,

(iv) $s^{\text{Sal}}(z_i, a_j) = \frac{|N(z_i,a_j)|}{\sqrt{d(z_i)d(a_j)}} = \frac{1}{\sqrt{2n}} > \frac{1}{\sqrt{2}n} > s^{\text{Sal}}(v_0, w_0)$.

**Jaccard similarity index ($s^{\text{Jac}}$):** we have $s^{\text{Jac}}(v_0, w_0) = \frac{|N(v_0,w_0)|}{|N(v_0)\cup N(w_0)|} = \frac{1}{n^2+|N(v_0)|}$, which gives us $s^{\text{Jac}}(v_0, w_0) \leq \frac{1}{n^2+2}$. We then have

$$s^{\text{Jac}}(v_i, v_j) = \frac{|N(v_i, v_j)|}{|N(v_i) \cup N(v_j)|} \geq \frac{1}{n+1} > \frac{1}{n^2+2} > s^{\text{Jac}}(v_0, w_0),$$

which satisfies point (b) of the lemma. Now, to satisfy point (c) of the lemma we show the following:

(i) $s^{\text{Jac}}(v_i, a_j) = \frac{|N(v_i,a_j)|}{|N(v_i)\cup N(a_j)|} \geq \frac{1}{n+2} > \frac{1}{n^2+2} \geq s^{\text{Jac}}(v_0, w_0)$,

(ii) $s^{\text{Jac}}(d_i, a_0) = \frac{|N(d_i,a_0)|}{|N(d_i)\cup N(a_0)|} = \frac{1}{2} > \frac{1}{n^2+2} \geq s^{\text{Jac}}(v_0, w_0)$,

(iii) $s^{\text{Jac}}(d_i, d_j) = \frac{|N(d_i,d_j)|}{|N(d_i)\cup N(d_j)|} = 1 > s^{\text{Jac}}(v_0, w_0)$,

(iv) $s^{\text{Jac}}(z_i, a_j) = \frac{|N(z_i,a_j)|}{|N(z_i)\cup N(a_j)|} = \frac{1}{n+1} > \frac{1}{n^2+2} \geq s^{\text{Jac}}(v_0, w_0)$.

**Sørensen similarity index ($s^{\text{Sør}}$):** we have $s^{\text{Sør}}(v_0, w_0) = \frac{2|N(v_0,w_0)|}{d(v_0)+d(w_0)} = \frac{2}{n^2+1+d(v_0)}$, which gives us $s^{\text{Sør}}(v_0, w_0) \leq \frac{2}{n^2+3}$. We then have

$$s^{\text{Sør}}(v_i, v_j) = \frac{2|N(v_i, v_j)|}{d(v_i) + d(v_j)} \geq \frac{1}{n+1} > \frac{2}{n^2+3} \geq s^{\text{Sør}}(v_0, w_0),$$

which satisfies point (b) of the lemma. Now, to satisfy point (c) of the lemma we show the following:

(i) $s^{\text{Sør}}(v_i, a_j) = \frac{2|N(v_i,a_j)|}{d(v_i)+d(a_j)} \geq \frac{2}{n+3} > \frac{2}{n^2+3} \geq s^{\text{Sør}}(v_0, w_0)$,

(ii) $s^{\text{Sør}}(d_i, a_0) = \frac{2|N(d_i,a_0)|}{d(d_i)+d(a_0)} = \frac{2}{3} > \frac{2}{n^2+3} \geq s^{\text{Sør}}(v_0, w_0)$,

(iii) $s^{\text{Sør}}(d_i, d_j) = \frac{2|N(d_i,d_j)|}{d(d_i)+d(d_j)} = 1 > s^{\text{Sør}}(v_0, w_0)$,

(iv) $s^{\text{Sør}}(z_i, a_j) = \frac{2|N(z_i,a_j)|}{d(z_i)+d(a_j)} = \frac{2}{n+2} > \frac{2}{n^2+3} \geq s^{\text{Sør}}(v_0, w_0)$.

**Hub Promoted similarity index ($s^{\text{HPI}}$):** we set $\gamma = n^2$. We then have $s^{\text{HPI}}(v_0, w_0) = \frac{|N(v_0,w_0)|}{\min(d(v_0),d(w_0))} = \frac{1}{\min(d(v_0),n^2+1)}$, which gives us $s^{\text{HPI}}(v_0, w_0) \leq \frac{1}{n^2+1}$. We then have

$$s^{\text{HPI}}(v_i, v_j) = \frac{|N(v_i, v_j)|}{\min(d(v_i), d(v_j))} \geq \frac{\gamma}{\min(d(v_i), d(v_j))} \geq \frac{n^2}{n^2+n} > \frac{1}{n^2+1} \geq s^{\text{HPI}}(v_0, w_0),$$

which satisfies point (b) of the lemma. Now, to satisfy point (c) of the lemma we show the following:

(i) $s^{\text{HPI}}(v_i, a_j) = \frac{|N(v_i,a_j)|}{\min(d(v_i),d(a_j))} \geq \frac{1}{2} > \frac{1}{n^2+1} \geq s^{\text{HPI}}(v_0, w_0)$,

(ii) $s^{\text{HPI}}(d_i, a_0) = \frac{|N(d_i,a_0)|}{\min(d(d_i),d(a_0))} = 1 > s^{\text{HPI}}(v_0, w_0)$,

(iii) $s^{\text{HPI}}(d_i, d_j) = \frac{|N(d_i,d_j)|}{\min(d(d_i),d(d_j))} = 1 > s^{\text{HPI}}(v_0, w_0)$,

(iv) $s^{\text{HPI}}(z_i, a_j) = \frac{|N(z_i,a_j)|}{\min(d(z_i),d(a_j))} = \frac{1}{2} > \frac{1}{n^2+1} \geq s^{\text{HPI}}(v_0, w_0)$.

**Hub Depressed similarity index ($s^{\text{HDI}}$):** we have $s^{\text{HDI}}(v_0, w_0) = \frac{|N(v_0,w_0)|}{\max(d(v_0),d(w_0))} = \frac{1}{\max(d(v_0),n^2+1)}$, which gives us $s^{\text{HDI}}(v_0, w_0) \leq \frac{1}{n^2+1}$. We then have

$$s^{\text{HDI}}(v_i, v_j) = \frac{|N(v_i, v_j)|}{\max(d(v_i), d(v_j))} \geq \frac{1}{n+1} > \frac{1}{n^2+1} \geq s^{\text{HDI}}(v_0, w_0),$$

which satisfies point (b) of the lemma. Now, to satisfy point (c) of the lemma we show the following:

(i) $s^{\text{HDI}}(v_i, a_j) = \frac{|N(v_i,a_j)|}{\max(d(v_i),d(a_j))} \geq \frac{1}{n+1} > \frac{1}{n^2+1} \geq s^{\text{HDI}}(v_0, w_0)$,

(ii) $s^{\text{HDI}}(d_i, a_0) = \frac{1|N(d_i,a_0)|}{\max(d(d_i),d(a_0))} = \frac{1}{2} > \frac{1}{n^2+1} \geq s^{\text{HDI}}(v_0, w_0)$,

(iii) $s^{\mathrm{HDI}}(d_i, d_j) = \frac{|N(d_i,d_j)|}{\max(d(d_i),d(d_j))} = 1 > s^{\mathrm{HDI}}(v_0, w_0)$,

(iv) $s^{\mathrm{HDI}}(z_i, a_j) = \frac{|N(z_i,a_j)|}{\max(d(z_i),d(a_j))} = \frac{1}{n} > \frac{1}{n^2+1} \geq s^{\mathrm{HDI}}(v_0, w_0)$.

**Leicht-Holme-Newman similarity index ($s^{\mathrm{LHN}}$):** we have $s^{\mathrm{LHN}}(v_0, w_0) = \frac{|N(v_0,w_0)|}{d(v_0)d(w_0)} = \frac{1}{d(v_0)(n^2+1)}$, which gives us $s^{\mathrm{LHN}}(v_0, w_0) \leq \frac{1}{2(n^2+1)}$. We then have

$$s^{\mathrm{LHN}}(v_i, v_j) = \frac{|N(v_i, v_j)|}{d(v_i)d(v_j)} \geq \frac{1}{(n+1)^2} > \frac{1}{2(n^2+1)} \geq s^{\mathrm{LHN}}(v_0, w_0),$$

which satisfies point (b) of the lemma. Now, to satisfy point (c) of the lemma we show the following:

(i) $s^{\mathrm{LHN}}(v_i, a_j) = \frac{|N(v_i,a_j)|}{d(v_i)d(a_j)} \geq \frac{1}{2(n+1)} > \frac{1}{2(n^2+1)} \geq s^{\mathrm{LHN}}(v_0, w_0)$,

(ii) $s^{\mathrm{LHN}}(d_i, a_0) = \frac{|N(d_i,a_0)|}{d(d_i)d(a_0)} = \frac{1}{2} > \frac{1}{2(n^2+1)} \geq s^{\mathrm{LHN}}(v_0, w_0)$,

(iii) $s^{\mathrm{LHN}}(d_i, d_j) = \frac{|N(d_i,d_j)|}{d(d_i)d(d_j)} = 1 > s^{\mathrm{LHN}}(v_0, w_0)$,

(iv) $s^{\mathrm{LHN}}(z_i, a_j) = \frac{|N(z_i,a_j)|}{d(z_i)d(a_j)} = \frac{1}{2n} > \frac{1}{2(n^2+1)} \geq s^{\mathrm{LHN}}(v_0, w_0)$.

Next, we will prove the lemma for the indices in $\{s^{\mathrm{AA}}, s^{\mathrm{RA}}\}$. To this end, the network $G$ will be created as follows (an example of network $G$ is illustrated in Figure S4):

- **The set of nodes:** For every $v_i \in \{v_0, \ldots, v_n\}$ we create nodes $v_i$, $w_{i,1}$ and $w_{i,2}$, as well as a node $a_i$. Additionally, we create $n$ nodes, $z_1, \ldots, z_n$, as well as $2n - k + 3$ nodes, $d_1, \ldots, d_{2n-k+3}$.
- **The set of edges:** We connect nodes in $\{v_1, \ldots, v_n\}$ into a clique. For every node $w_{i,j}$ we create edges $(v_i, w_{i,j})$ and $(a_i, w_{i,j})$. For every node $z_i$ we create edges $(z_i, v_1), \ldots, (z_i, v_n)$. Finally, for every node $d_i$ we create an edge $(d_i, v_0)$.

We will show that for any given similarity index $s \in \{s^{\mathrm{AA}}, s^{\mathrm{RA}}\}$ the network $G$ with $h = (w_{0,1}, w_{0,2})$ and $\bar{E}^* = \{e_1, ..., e_n\} : e_i = (w_{i,1}, w_{i,2})$ satisfies the lemma. Notice that for any $(w_{i,1}, w_{i,2}) \in \bar{E}^*$ we have $s^{\mathrm{AA}}(e_i) = \frac{1}{\log(2)} + \frac{1}{\log(2n+1-r_i)}$ and $s^{\mathrm{RA}}(e_i) = \frac{1}{2} + \frac{1}{2n+1-r_i}$, while for $h = (w_{0,1}, w_{0,2})$ we have $s^{\mathrm{AA}}(h) = \frac{1}{\log(2)} + \frac{1}{\log(2n-k+3)}$ and $s^{\mathrm{RA}}(h) = \frac{1}{2} + \frac{1}{2n-k+3}$. Therefore, $s(e_i) = s(h)$ when $2n + 1 - r_i = 2n - k + 3$, which is the case for $r_i = k - 2$. Since the value of $s(e_i)$ increases with $r_i$, we have that $s(e_i) < s(h)$ when $r_i < k - 2$, and analogously $s(e_i) > s(h)$ when $r_i > k - 2$. This proves point (a) of the lemma.

Notice also that since $N(w_{0,1}, w_{0,2}) \neq \emptyset$, for every similarity index $s \in \{s^{\mathrm{AA}}, s^{\mathrm{RA}}\}$ we have that $s(h) > 0$. Therefore, for every non-edge $e$ where the ends do not have any common neighbors, we have that $s(e) < s(h)$ in both network $(V, E)$ and network $(V, E \setminus R)$ (notice that the removal of $R$ cannot increase the number of common neighbors for any pair of nodes), hence any such $e$ satisfies point (c) of the lemma.

Finally, notice that for any similarity index $s \in \{s^{\mathrm{AA}}, s^{\mathrm{RA}}\}$ we have $s(d_i, d_j) = s(d_i, w_{i,j})$ and $s(v_i, w_{i,j}) = s(z_i, w_{i,j})$. Now, we have to show that for every similarity index the following conditions are satisfied:

- for every non-edge $e \in R$ we have $s(e) > s(h)$ in the network $(V, E \setminus R)$ to prove point (b) of the lemma,
- each of the following non-edges:
  (i) $(v_i, a_i)$,
  (ii) $(z_i, w_{i,j})$,
  (iii) $(z_i, z_j)$,
  (iv) $(d_i, d_j)$.

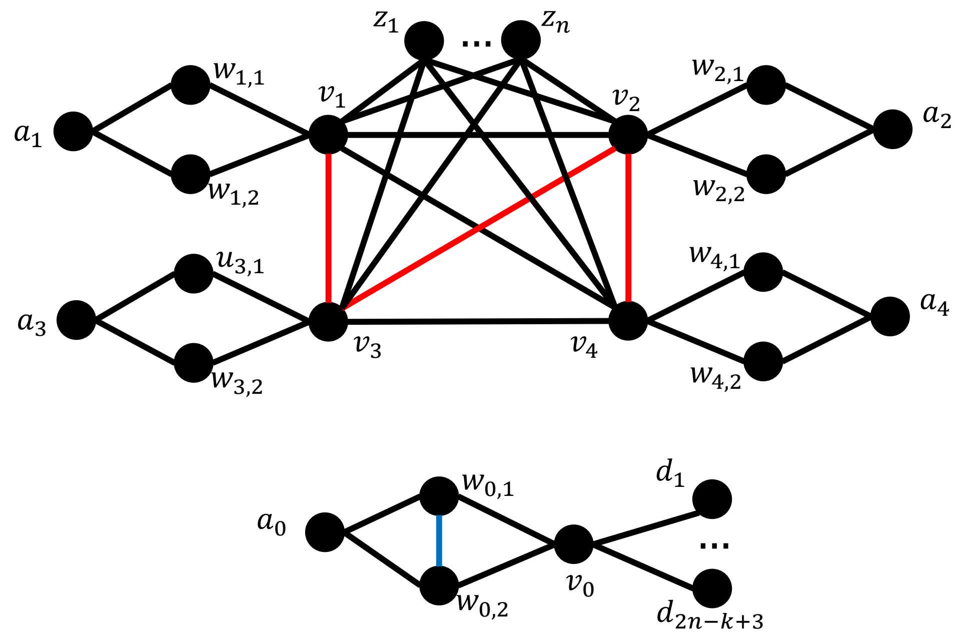


Figure S4: An illustration of the network used in the proof of Lemma 2 for a similarity index $s \in \{s^{\mathrm{AA}}, s^{\mathrm{RA}}\}$. The red edges are allowed to be removed, while the blue non-edge, $(w_{0,1}, w_{0,2})$, is the one to be hidden.

either has always greater or always smaller value of $s$ than $h$ (to prove point (c) of the lemma). For all other non-edges either their ends have no common neighbors, or their similarity is the same as one of the non-edges we listed above.

In what follows, we will use the following facts:

- $d(v_i) \in [n+2, 2n+1]$,
- $d(w_{i,j}) = 2$,
- $d(a_i) = 2$,
- $d(z_i) = n$.

**Adamic-Adar similarity index ($s^{\mathrm{AA}}$):** we have $s^{\mathrm{AA}}(h) = \frac{1}{\log(2)} + \frac{1}{\log(d(v_0))}$, which gives us $s^{\mathrm{AA}}(h) \geq \frac{1}{\log(2)} + \frac{1}{\log(2n-1)} > 3$ as well as $s^{\mathrm{AA}}(h) \leq \frac{1}{\log(2)} + \frac{1}{\log(n+3)} < 5$. We then have

$$s^{\mathrm{AA}}(v_i, v_j) = \frac{n}{\log(n)} + \sum_{v_l \in N(v_i,v_j)} \frac{1}{\log(d(v_l))} \geq \frac{n}{\log(n)} > 5 > s^{\mathrm{AA}}(h),$$

which satisfies point (b) of the lemma. Now, to satisfy point (c) of the lemma we show the following:

(i) $s^{\mathrm{AA}}(v_i, a_i) = \frac{2}{\log(2)} > 6 > s^{\mathrm{AA}}(h)$,

(ii) $s^{\mathrm{AA}}(z_i, w_{i,j}) = \frac{1}{\log(d(v_i))} \leq \frac{1}{\log(n+2)} < 3 < s^{\mathrm{AA}}(h)$,

(iii) $s^{\mathrm{AA}}(z_i, z_j) = \sum_{v_l \in N(v_i,v_j)} \frac{1}{\log(d(v_l))} \leq \frac{n}{\log(n+2)} > 5 > s^{\mathrm{AA}}(h)$,

(iv) $s^{\mathrm{AA}}(d_i, d_j) = \frac{1}{\log(d(v_0))} = \frac{1}{\log(2n-k+3)} < 3 < s^{\mathrm{AA}}(h)$.

**Resource Allocation similarity index ($s^{\mathrm{RA}}$):** we have $s^{\mathrm{RA}}(h) = \frac{1}{2} + \frac{1}{d(v_0)}$, which gives us $s^{\mathrm{RA}}(h) \leq \frac{1}{2} + \frac{1}{n+2}$, as well as $s^{\mathrm{RA}}(h) \geq \frac{1}{2} + \frac{1}{2n+1}$. We then have

$$s^{\mathrm{RA}}(v_i, v_j) = \frac{n}{n} + \sum_{v_l \in N(v_i,v_j)} \frac{1}{d(v_l)} \geq 1 > \frac{1}{2} + \frac{1}{n+2} \geq s^{\mathrm{RA}}(h),$$

which satisfies point (b) of the lemma. Now, to satisfy point (c) of the lemma we show the following:

(i) $s^{\mathrm{RA}}(v_i, a_i) = 1 > \frac{1}{2} + \frac{1}{n+2} \geq s^{\mathrm{RA}}(h)$,

(ii) $s^{\mathrm{RA}}(z_i, w_{i,j}) = \frac{1}{d(v_i)} \leq \frac{1}{n+2} < \frac{1}{2} + \frac{1}{2n+2} \leq s^{\mathrm{RA}}(h)$,

(iii) $s^{\mathrm{RA}}(z_i, z_j) = \sum_{v_l \in N(v_i,v_j)} \frac{1}{d(v_l)} \leq \frac{n}{n+2} > \frac{1}{2} + \frac{1}{n+2} \geq s^{\mathrm{RA}}(h)$,

(iv) $s^{\mathrm{RA}}(d_i, d_j) = \frac{1}{d(v_0)} = \frac{1}{3n-k+3} < \frac{1}{2} + \frac{1}{2n+2} \leq s^{\mathrm{RA}}(h)$.

□

Finally, we will use network $G$, the existence of which is shown in Lemma 2 to prove the NP-completeness of the problem of Evading Link Prediction for similarity indices other than Common Neighbors when the set of actions is limited to edge removal.

**Theorem 3.** *The problem of Evading Link Prediction is NP-complete for each of the following similarity indices: Salton [26], Jaccard [13], Sørensen [28], Hub Promoted [25], Hub Depressed [25], Leicht-Holme-Newman [19], Adamic-Adar [1] and Resource Allocation [33], even when the set of actions is limited to edge removal.*

*Proof.* The problem of Evading Link Prediction is trivially in NP, since computing $AUC$ and $AP$ before and after the removal of a given set of edges $R \subseteq \widehat{R}$ can be done in polynomial time for every similarity index.

Next, we will prove that the problem is NP-hard. To this end, we will give a reduction from the NP-complete problem of Finding $k$-Clique. This problem is defined by a network $G' = (V', E')$ and an integer $k \leq |V'|$. The goal is then to determine whether there exist $k$ nodes that induce a clique in $G'$.

We will assume that $k > 3$ (for $k \leq 3$ the problem can be solved in polynomial time). Now, for a given similarity index, $s \in \{s^{\mathrm{Sal}}, s^{\mathrm{Jac}}, s^{\mathrm{Sør}}, s^{\mathrm{HPI}}, s^{\mathrm{HDI}}, s^{\mathrm{LHN}}, s^{\mathrm{AA}}, s^{\mathrm{RA}}\}$, consider the following instance of the problem of Evading Link Prediction $(G, s, f, H, b, \widehat{A}, \widehat{R})$, where:

- $G$ is the network defined in Lemma 2,
- $s$ is the similarity index under consideration,
- $f$ is either the $AUC$ or the $AP$ metric,
- $H = \{h\}$, where $h$ is the non-edge defined in Lemma 2,
- $b = \frac{k(k-1)}{2}$, where $k$ is the parameter of the Finding $k$-Clique problem,
- $\widehat{A} = \emptyset$,
- $\widehat{R} = E'$.

Let us also introduce the following notation:

- $\Upsilon^{<}(R) = \{e \in \bar{E} \setminus \bar{E}^* : s(e) < s(h)\}$ in network $(V, E \setminus R)$,
- $\Upsilon^{>}(R) = \{e \in \bar{E} \setminus \bar{E}^* : s(e) > s(h)\}$ in network $(V, E \setminus R)$,
- $\Psi^{>}(R) = \{e \in \bar{E}^* : s(e) > s(h)\}$ in network $(V, E \setminus R)$,
- $\Psi^{=}(R) = \{e \in \bar{E}^* : s(e) = s(h)\}$ in network $(V, E \setminus R)$,
- $\Psi^{<}(R) = \{e \in \bar{E}^* : s(e) < s(h)\}$ in network $(V, E \setminus R)$.

Given Lemma 2, for every network $(V, E \setminus R) : R \subseteq \widehat{R}$, we have that:

$$AUC(E \setminus R, H) = \frac{|\Upsilon^{<}(R)| + |\Psi^{<}(R)| + \frac{1}{2}|\Psi^{=}(R)|}{|\bar{E}| + |R| - 1}.$$

This, as well as the fact that $\Psi^{>}(R) + \Psi^{=}(R) + \Psi^{<}(R) = n$, gives us:

$$AUC(E \setminus R, H) = \frac{|\Upsilon^{<}(R)| + n - |\Psi^{>}(R)| - \frac{1}{2}|\Psi^{=}(R)|}{|\bar{E}| + |R| - 1}.$$

We also have that:

$$AP(E \setminus R, H) = \frac{1}{|\Upsilon^{>}(G')| + |R| + 1 + |\Psi^{>}(G')| + \frac{1}{2}|\Psi^{=}(G')|}.$$

We can see that both $AUC$ and $AP$ decrease with the size of $R$, and also decrease with increasing values of $|\Psi^{>}(R)| + \frac{|\Psi^{=}(R)|}{2}$. Hence, since the goal is to minimize the values of $AUC$ and $AP$, we need to maximize the value of $|\Psi^{>}(R)| + \frac{|\Psi^{=}(R)|}{2}$, while removing as many edges as possible. We will first prove the following lemma regarding the values of $|\Psi^{>}(R)|$ and $|\Psi^{=}(R)|$.

**Lemma 3.** *We have that:*

- *if* $|\Psi^{>}(R)| \geq 3$ *then* $|\Psi^{=}(R)| \leq k - |\Psi^{>}(R)|$,
- *if* $|\Psi^{>}(R)| = 2$ *then* $|\Psi^{=}(R)| \leq k - 1$,
- *if* $|\Psi^{>}(R)| = 1$ *then* $|\Psi^{=}(R)| \leq k$,
- *if* $|\Psi^{>}(R)| = 0$ *then* $|\Psi^{=}(R)| \leq k + 2$.

*Proof.* Since the removal budget is $b = \frac{k(k-1)}{2}$, and since (as shown in Lemma 2) in order for edge $e_i$ to be part of $\Psi^{>}(R)$ (resp. $\Psi^{=}(R)$) we have to remove at least $k-1$ edges (resp. exactly $k-2$ edges) incident to $v_i$, then the following holds:

$$2\frac{k(k-1)}{2} \geq |\Psi^{>}(R)|(k-1) + |\Psi^{=}(R)|(k-2),$$

which gives us:

$$|\Psi^{=}(R)| \leq k - |\Psi^{>}(R)| + \frac{k - |\Psi^{>}(R)|}{k-2}.$$

Consequently:

- for $|\Psi^{>}(R)| \geq 3$ we have $|\Psi^{=}(R)| \leq k - |\Psi^{>}(R)| + \frac{k-3}{k-2}$, which results in $|\Psi^{=}(R)| \leq k - |\Psi^{>}(R)|$ since $|\Psi^{=}(R)|$ is integer,
- for $|\Psi^{>}(R)| = 2$ we have $|\Psi^{=}(R)| \leq k - 2 + \frac{k-2}{k-2} = k - 1$,
- for $|\Psi^{>}(R)| = 1$ we have $|\Psi^{=}(R)| \leq k - 1 + \frac{k-1}{k-2}$ which, since $k > 3$, results in $|\Psi^{=}(R)| \leq k - 1 + \frac{3}{2}$, which gives us $|\Psi^{=}(R)| \leq k$ since $|\Psi^{=}(R)|$ is integer,
- for $|\Psi^{>}(R)| = 0$ we have $|\Psi^{=}(R)| \leq k + \frac{k}{k-2} \leq k + 2$ since $k > 3$.

□

We will now show that $\Delta = k - \left(|\Psi^{>}(R)| + \frac{|\Psi^{=}(R)|}{2}\right) > 0$ for any $|\Psi^{<}(R)| < k$. Given Lemma 3:

- for $|\Psi^{>}(R)| \geq 3$ we have $\Delta \geq \frac{k}{2} - \frac{|\Psi^{>}(R)|}{2} > 0$ since $|\Psi^{<}(R)| < k$,

- for $|\Psi^{>}(R)| = 2$ we have $\Delta \geq \frac{k}{2} - \frac{3}{2} > 0$ since $k > 3$,
- for $|\Psi^{>}(R)| = 1$ we have $\Delta \geq \frac{k}{2} - 1 > 0$ since $k > 3$,
- for $|\Psi^{>}(R)| = 0$ we have $\Delta \geq \frac{k}{2} - 1 > 0$ since $k > 3$.

Therefore, for any $|\Psi^{>}(R)| < k$ we have that $k > |\Psi^{>}(R)| + \frac{|\Psi^{=}(R)|}{2}$. Hence, the optimal $R$ decreases the degree of at least $k$ nodes in $V$ by at least $k-1$ each (notice that we then have $|\Psi^{>}(R)| = k$). However, since the removal budget is $b = \frac{k(k-1)}{2}$, this is possible only if the nodes form a clique in $\widehat{R}$. Since $\widehat{R} = E'$, the nodes belonging to $R$ form a $k$-clique in $G'$, providing a solution to the given instance of the problem of Finding $k$-Clique. This concludes the proof. □

# S4 Proof of Theorem 4

**Theorem 4.** *Let $G' = (V, E')$ be a network, and let $(x, w)$ be a non-edge in $G'$. Furthermore, let $v$ be a node in $G'$ such that $v \in N_{G'}(x)$ and $v \notin N_{G'}(w)$. Finally, let $G$ be the network that results from adding $(v, w)$ to $G'$, i.e., $G = (V, E)$ where $E = E' \cup \{(v, w)\}$. Then, for every similarity index, $s \in \mathcal{S}$, we have:*

$$s_{G'}(x, w) \leq s_G(x, w)$$

*Proof.* From the definitions of $G$ and $G'$, we know that:

- $d_{G'}(x) = d_G(x)$;
- $d_{G'}(w) = d_G(w) - 1$;
- $|N_{G'}(x, w)| = |N_G(x, w)| - 1$;
- $\forall u \in N_{G'}(x, w) : d_{G'}(u) = d_G(u)$.

With these facts in mind, we will now handle each similarity index in $\mathcal{S}$ separately. In particular:

- For $s^{\text{CN}}(x, w) = |N(x, w)|$, we know that $s^{\text{CN}}_{G'}(x, w) < s^{\text{CN}}_G(x, w)$ because $|N_{G'}(x, w)| = |N_G(x, w)| - 1$.
- For $s^{\text{Sal}}(x, w) = \frac{|N(x,w)|}{\sqrt{d(x)d(w)}}$, to prove that $s^{\text{Sal}}_{G'}(x, w) \leq s^{\text{Sal}}_G(x, w)$, it suffices to prove that:

$$\frac{|N_G(x, w)| - 1}{\sqrt{d_G(x)(d_G(w) - 1)}} \leq \frac{|N_G(x, w)|}{\sqrt{d_G(x)d_G(w)}}$$

  This holds if and only if: $|N_G(x, w)|^2 \leq d_G(w)(2|N_G(x, w)| - 1)$. This, in turn, always holds since $|N_G(x, w)| \leq d_G(w)$ and $|N_G(x, w)| \leq 2|N_G(x, w)| - 1$.

- For $s^{\text{Jac}}(x, w) = \frac{|N(x,w)|}{|N(x) \cup N(w)|}$, we know that the following holds in any network: $|N(x) \cup N(w)| = d(x) + d(w) - |N(x, w)|$. Based on this, to prove that $s^{\text{Jac}}_{G'}(x, w) < s^{\text{Jac}}_G(x, w)$, it suffices to note that:

$$\frac{|N_G(x, w)| - 1}{d_G(x) + (d_G(w) - 1) - (|N_G(x, w)| - 1)} < \frac{|N_G(x, w)|}{d_G(x) + d_G(w) - |N_G(x, w)|}$$

- For $s^{\text{Sør}}(x, w) = \frac{2|N(x,w)|}{d(x)+d(w)}$, to prove that $s^{\text{Sør}}_{G'}(x, w) \leq s^{\text{Sør}}_G(x, w)$, it suffices to prove that:

$$\frac{2|N_G(x, w)| - 2}{d_G(x) + d_G(w) - 1} \leq \frac{2|N_G(x, w)|}{d_G(x) + d_G(w)}$$

  This holds if and only if: $|N_G(x, w)| \leq d_G(x) + d_G(w)$. This, in turn, always holds since $N_G(x, w) \subseteq N_G(x)$ and $N_G(x, w) \subseteq N_G(w)$.

- For $s^{\text{HPI}}(x, w) = \frac{|N(x,w)|}{\min(d(x),d(w))}$, let us first consider the case where $d_G(x) < d_G(w)$. In this case, we have: $\min(d_G(x), d_G(w)) = d_G(x)$ and $\min(d_{G'}(x), d_{G'}(w)) = \min(d_G(x), d_G(w) - 1) = d_G(x)$. This implies that $s^{\text{HPI}}_{G'}(x, w) < s^{\text{HPI}}_G(x, w)$, since:

$$\frac{|N_{G'}(x, w)|}{\min(d_{G'}(x), d_{G'}(w))} = \frac{|N_G(x, w)| - 1}{d_G(x)} < \frac{|N_G(x, w)|}{d_G(x)} = \frac{|N_G(x, w)|}{\min(d_G(x), d_G(w))}$$

  On the other hand, if $d_G(x) \geq d_G(w)$, then $\min(d_G(x), d_G(w)) = d_G(w)$ and $\min(d_{G'}(x), d_{G'}(w)) = \min(d_G(x), d_G(w) - 1) = d_G(w) - 1$. Based on this, in order to prove that $s^{\text{HPI}}_{G'}(x, w) \leq s^{\text{HPI}}_G(x, w)$, we need to prove that:

$$\frac{|N_G(x, w)| - 1}{d_G(w) - 1} \leq \frac{|N_G(x, w)|}{d_G(w)}$$

  This holds if and only if: $|N_G(x, w)| \leq d_G(w)$. This, in turn, always holds since $N_G(x, w) \subseteq N_G(w)$.

- For $s^{\text{HDI}}(x,w) = \frac{|N(x,w)|}{\max(d(x),d(w))}$, let us first consider the case where $d_G(x) \geq d_G(w)$. In this case, we have: $\max(d_G(x), d_G(w)) = d_G(x)$ and $\max(d_{G'}(x), d_{G'}(w)) = \max(d_G(x), d_G(w)-1) = d_G(x)$. This implied that $s^{\text{HDI}}_{G'}(x,w) < s^{\text{HDI}}_{G}(x,w)$, since:

$$\frac{|N_{G'}(x,w)|}{\max(d_{G'}(x), d_{G'}(w))} = \frac{|N_G(x,w)|-1}{d_G(x)} < \frac{|N_G(x,w)|}{d_G(x)} = \frac{|N_G(x,w)|}{\max(d_G(x), d_G(w))}$$

  On the other hand, if $d_G(x) < d_G(w)$, then $\max(d_G(x), d_G(w)) = d_G(w)$ and $\max(d_{G'}(x), d_{G'}(w)) = \max(d_G(x), d_G(w)-1) = d_G(w)-1$. Based on this, in order to prove that $s^{\text{HDI}}_{G'}(x,w) \leq s^{\text{HDI}}_{G}(x,w)$, we need to prove that:

$$\frac{|N_G(x,w)|-1}{d_G(w)-1} \leq \frac{|N_G(x,w)|}{d_G(w)}$$

  This holds if and only if: $|N_G(x,w)| \leq d_G(w)$. This, in turn, always holds since $N_G(x,w) \subseteq N_G(w)$.

- For $s^{\text{LHN}}(x,w) = \frac{|N(x,w)|}{d(x)d(w)}$, to prove that $s^{\text{LHN}}_{G'}(x,w) \leq s^{\text{LHN}}_{G}(x,w)$, it suffices to prove that:

$$\frac{|N_G(x,w)|-1}{d_G(x)(d_G(w)-1)} \leq \frac{|N_G(x,w)|}{d_G(x)d_G(w)}$$

  This holds if and only if: $|N_G(x,w)| \leq d_G(w)$. This, in turn, always holds since $N_G(x,w) \subseteq N_G(w)$.

- For $s^{\text{AA}}(x,w) = \sum_{u \in N(x,w)} \frac{1}{\log(d(u))}$, to prove that $s^{\text{AA}}_{G'}(x,w) \leq s^{\text{AA}}_{G}(x,w)$, it suffices to note that:

$$\sum_{u \in N_{G'}(x,w)} \frac{1}{\log(d_{G'}(u))} = \left( \sum_{u \in N_G(x,w)} \frac{1}{\log(d_G(u))} \right) - \frac{1}{\log(d_G(v))} \leq \sum_{u \in N_G(x,w)} \frac{1}{\log(d_G(u))}$$

- For $s^{\text{RA}}(x,w) = \sum_{u \in N(x,w)} \frac{1}{d(u)}$, to prove that $s^{\text{RA}}_{G'}(x,w) \leq s^{\text{RA}}_{G}(x,w)$, it suffices to note that:

$$\sum_{u \in N_{G'}(x,w)} \frac{1}{d_{G'}(u)} = \left( \sum_{u \in N_G(x,w)} \frac{1}{d_G(u)} \right) - \frac{1}{d_G(v)} \leq \sum_{u \in N_G(x,w)} \frac{1}{d_G(u)}$$

□

# S5 The Pseudo-code of CTR

The pseudo-code of CTR is presented in Algorithm 1. Specifically, in Line 1, out of all the edges that can be removed (i.e., all the edges in $\widehat{R}$), the algorithm narrows the search to only the subset $R' \subseteq \widehat{R}$ in which every edge has at least one end that belongs to some non-edge in $H$. After that, in Lines 3 to 13, the algorithm computes for every edge, $(v, w) \in R'$, a score, $\sigma_{(v,w)}$, which reflects the gain from removing $(v, w)$ from the network. More specifically, this score is computed by counting the number of closed triads that contain $(v, w)$ and two other edges, one of which is in $H$. The edge with the greatest gain is chosen in Line 14, and removed from the network in Line 16. This entire process is repeated until the budget, $b$, runs out.

**ALGORITHM 1:** *Closed-Triad-Removal (CTR)*

**Input:** A network, $(V, E)$, a budget, $b \in \mathbb{N}$, a set of edges that can be removed, $\widehat{R} \subseteq E$, and a set of non-edges to be hidden, $H \subset \bar{E}$.

1 $R' \leftarrow \Big\{(v, w) \in \widehat{R} : \Big(\exists x \in N(v) : (x,v) \in H\Big) \vee \Big(\exists x \in N(w) : (x,w) \in H\Big)\Big\}$;<br>
2 **for** $i = 1, \ldots, b$ **do**<br>
3     **for** $(v, w) \in R'$ **do**<br>
4         $\sigma_{(v,w)} \leftarrow 0$;<br>
5     **end**<br>
6     **for** $(x, w) \in H$ **do**<br>
7         **for** $v \in N(x, w)$ **do**<br>
8             **if** $\big((v, w) \in E\big) \wedge \big((v, x) \in E\big)$ **then**<br>
9                 **if** $(v, w) \in R'$ **then** $\sigma_{(v,w)} \leftarrow \sigma_{(v,w)} + 1$;<br>
10                 **if** $(v, x) \in R'$ **then** $\sigma_{(v,x)} \leftarrow \sigma_{(v,x)} + 1$;<br>
11             **end**<br>
12         **end**<br>
13     **end**<br>
14     $(v^*, w^*) \leftarrow \arg\max_{(v,w) \in R'} \sigma_{(v,w)}$;<br>
15     **if** $\sigma_{(v^*,w^*)} > 0$ **then**<br>
16         $E \leftarrow E \setminus (v^*, w^*)$;<br>
17     **end**<br>
18 **end**

The complexity of such a naive implementation of CTR is $\mathcal{O}(b|H||V|)$. This is because for every non-edge $(x, w) \in H$ (there are $|H|$ such edges), the algorithm updates the score of every $(v, w) : v \in N(w)$ (there are at most $|V|$ such edges) and updates the score of every $(v, x) : v \in N(x)$ (again there are at most $|V|$ such edges); this process is repeated $b$ times.

Note that when $|H| = \omega(\log(|V|))$, an implementation utilizing a priority queue would be faster, with a complexity of $\mathcal{O}(|H||V| + b|V|\log(|V|))$; see Algorithm 2. More specifically, this implementation utilizes a priority queue such as, e.g., a heap [6]. Such a priority queue can be built in time $\mathcal{O}(|H||V|)$. The cost of all operations of extracting an element with maximal score is then $\mathcal{O}(b\log(|H||V|))$, which equals $\mathcal{O}(b\log(|V|))$, since $|H|$ is at most $\Theta(|V|^2)$. However, the cost of updating the scores becomes $\mathcal{O}(b|V|\log(|V|))$, since it can only involve decreasing the scores (decreasing scores is realized by removing an element and adding it with a lower score).

**ALGORITHM 2:** *Closed-Triad-Removal* (CTR) with priority queue

**Input:** A network, $(V, E)$, a budget, $b \in \mathbb{N}$, a set of edges that can be removed, $\widehat{R} \subseteq E$, and a set of non-edges to be hidden, $H \subset \bar{E}$.

$R' \leftarrow \Big\{(v,w) \in \widehat{R} : \big(\exists x \in N(v) : (x,v) \in H\big) \vee \big(\exists x \in N(w) : (x,w) \in H\big)\Big\}$;

**for** $(v,w) \in R'$ **do**
    $\sigma_{(v,w)} \leftarrow 0$;
**end**
**for** $(x,w) \in H$ **do**
    **for** $v \in N(x,w)$ **do**
        **if** $\big((v,w) \in E\big) \wedge \big((v,x) \in E\big)$ **then**
            **if** $(v,w) \in R'$ **then** $\sigma_{(v,w)} \leftarrow \sigma_{(v,w)} + 1$;
            **if** $(v,x) \in R'$ **then** $\sigma_{(v,x)} \leftarrow \sigma_{(v,x)} + 1$;
        **end**
    **end**
**end**
**for** $i = 1, \ldots, b$ **do**
    $(v^*, w^*) \leftarrow \arg\max_{(v,w) \in R'} \sigma_{(v,w)}$;
    **if** $\sigma_{(v^*,w^*)} > 0$ **then**
        $E = E \setminus (v^*, w^*)$;
        **for** $z \in N(v^*) \cup N(w^*)$ **do**
            **if** $(z,v^*) \in H \wedge (z,w^*) \in R'$ **then** $\sigma_{(z,w^*)} \leftarrow \sigma_{(z,w^*)} - 1$;
            **if** $(z,w^*) \in H \wedge (z,v^*) \in R'$ **then** $\sigma_{(z,v^*)} \leftarrow \sigma_{(z,v^*)} - 1$;
        **end**
    **end**
**end**

# S6 The Pseudo-code of OTC

The pseudo-code of OTC is presented in Algorithm 3. In Line 1, out of all the non-edges that can be added (i.e., all the edges in $\widehat{A}$), the algorithm narrows the search to only the subset $A' \subseteq \widehat{A}$ in which every non-edge has at least one end that belongs to some non-edge in $H$. In Lines 3 to 9, the algorithm computes for every non-edge $(v, w) \in A'$ a score, $\sigma_{(v,w)}$, which reflects the gain from adding $(v, w)$ to the network. Here, Lines 4 and 5 ensure that the algorithm does not increase the number of common neighbours of some non-edge in $H$. This way, the algorithm never accidentally makes any non-edge in $H$ more exposed. Moving on to Line 7, it counts the non-edges whose number of common neighbours will increase as a result of adding $(v, w)$. In Lines 10 to 12, the algorithm selects the non-edge with the highest score, and adds it to the network if it is beneficial to do so. This entire process is repeated until the budget, $b$, runs out.

**ALGORITHM 3:** *Open-Triad-Creation (OTC)*

**Input:** A network, $(V, E)$, a budget, $b \in \mathbb{N}$, a set of edges that can be added, $\widehat{A} \subseteq \bar{E} \setminus H$, and a set of non-edges to be hidden, $H \subset \bar{E}$.

1 $A' \leftarrow \left\{(v, w) \in \widehat{A} : \left(\exists u \in N(v) : (u,v) \in H\right) \vee \left(\exists u \in N(w) : (u,w) \in H\right)\right\}$;
2 **for** $i = 1, \dots, b$ **do**
3     **for** $(v, w) \in A'$ **do**
4         **if** $\exists\, u \in V : \left((v,u) \in E \,\wedge\, (w,u) \in H\right) \vee \left((w,u) \in E \,\wedge\, (v,u) \in H\right)$ **then**
5             $\sigma_{(v,w)} \leftarrow -\infty$;
6         **else**
7             $\sigma_{(v,w)} \leftarrow \left|\left(N_{\widehat{G}}(v) \cup N_{\widehat{G}}(w)\right) \setminus N_{\widehat{G}}(v, w)\right|$, where $\widehat{G} = (V, E)$;
8         **end**
9     **end**
10     $(v^*, w^*) \leftarrow \arg\max_{(v,w) \in A'} \sigma_{(v,w)}$;
11     **if** $\sigma_{(v^*,w^*)} > -\infty$ **then**
12         $E \leftarrow E \cup (v^*, w^*)$;
13     **end**
14 **end**

The complexity of such a naive implementation of OTC is $\mathcal{O}(b|H||V|^2)$. In more detail, computing a score, $\sigma_{(v,w)}$, for each non-edge, $(v, w) \in A'$, can be done in time linear in $|V|$ for each of the $|H||V|$ non-edges. Searching for a non-edge in $A'$ with the maximal score takes $b|H||V|$ operations. Finally, updating the scores after adding each of the $b$ edges can be done in time linear in $|V|$. Next, we will present a more efficient implementation, the complexity of which is $\mathcal{O}(|H||V|^2 + b|V|\log(|V|))$ when using a priority queue, and is $\mathcal{O}(|H||V|^2 + b|H||V|)$ without a priority queue.

Algorithm 4 presents a more efficient implementation of OTC compared to Algorithm 3. The complexity of this implementation is $\mathcal{O}(|H||V|^2 + b|H||V|)$. Here, the $|H||V|^2$ term comes from computing an initial score, $\sigma_{(v,w)}$, for each non-edge, $(v, w) \in A'$ (this can be done in time linear in $|V|$ for each of the $|H||V|$ non-edges). The $b|H||V|$ term comes from searching for a non-edge in $\widehat{A}$ with the maximal score. Finally, updating the scores after adding each of the $b$ edges can be done in time linear in $|V|$.

Notice that when $|H| = \omega(\log(|V|))$ and $b = \omega(|V|)$, an implementation utilizing a priority queue (such as, e.g., a heap [6]) would be faster. The complexity of such an implementation is $\mathcal{O}(|H||V|^2 + b|V|\log(|V|))$. In more detail, a priority queue can be built in time $\mathcal{O}(|H||V|^2)$. The cost of all operations of extracting an element with maximal score is then $\mathcal{O}(b\log(|H||V|))$, which equals $\mathcal{O}(b\log(|V|))$, since $|H|$ is at most $\Theta(|V|^2)$. However, the cost of updating the scores is now $\mathcal{O}(b|V|\log(|V|))$, since it could involve either increasing or decreasing the scores (decreasing scores is realized by removing an element and adding it with a lower score).

**ALGORITHM 4:** A more efficient implementation of *Open-Triad-Creation (OTC)*

**Input:** A network, $(V, E)$, a budget, $b \in \mathbb{N}$, a set of edges that can be added, $\widehat{A} \subseteq \bar{E} \setminus H$, and a set of non-edges to be hidden, $H \subset \bar{E}$.

$A' \leftarrow \Big\{(v, w) \in \widehat{A} : \big(\exists u \in N(v) : (u, v) \in H\big) \vee \big(\exists u \in N(w) : (u, w) \in H\big)\Big\}$;
**for** $(v, w) \in A'$ **do**
  **if** $\exists_{u \in V} \big((v, u) \in E \wedge (w, u) \in H\big) \vee \big((w, u) \in E \wedge (v, u) \in H\big)$ **then**
    $\sigma_{(v,w)} \leftarrow -\infty$;
  **else**
    $\sigma_{(v,w)} \leftarrow \left|\big(N_{(V,E)}(v) \cup N_{(V,E)}(w)\big) \setminus N_{(V,E)}(v, w)\right|$;
  **end**
**end**
**for** $i = 1, \ldots, b$ **do**
  $(v^*, w^*) \leftarrow \arg\max_{(v,w) \in A'} \sigma_{(v,w)}$;
  **if** $\sigma_{(v^*,w^*)} > -\infty$ **then**
    $E \leftarrow E \cup (v^*, w^*)$;
    **for** $u \in V \setminus \{v^*, w^*\}$ **do**
      **if** $(u, v^*) \in A'$ **then**
        **if** $(u, w^*) \in H$ **then** $\sigma_{(u,v^*)} \leftarrow -\infty$;
        **if** $(u, w^*) \in E \wedge \sigma_{(u,v^*)} > -\infty$ **then** $\sigma_{(u,v^*)} \leftarrow \sigma_{(u,v^*)} - 1$;
        **if** $(u, w^*) \in \bar{E} \wedge \sigma_{(u,v^*)} > -\infty$ **then** $\sigma_{(u,v^*)} \leftarrow \sigma_{(u,v^*)} + 1$;
      **end**
      **if** $(u, w^*) \in A'$ **then**
        **if** $(u, v^*) \in H$ **then** $\sigma_{(u,w^*)} \leftarrow -\infty$;
        **if** $(u, v^*) \in E \wedge \sigma_{(u,w^*)} > -\infty$ **then** $\sigma_{(u,w^*)} \leftarrow \sigma_{(u,w^*)} - 1$;
        **if** $(u, v^*) \in \bar{E} \wedge \sigma_{(u,w^*)} > -\infty$ **then** $\sigma_{(u,w^*)} \leftarrow \sigma_{(u,w^*)} + 1$;
      **end**
    **end**
  **end**
**end**

# S7 Experimental Evaluation

## S7.1 Networks Considered in Our Study

In our experiments, we considered both real-life networks as well as randomly-generated networks. As for the random ones, they were generated using the following standard models:

- *Scale-free* networks, generated using the Barabasi-Albert model [2]: We denote such a network by $\mathit{ScaleFree}(n, d)$, where $n$ is the number of nodes and $d$ is the number of links added with each node;
- *Small-world* networks, generated using the Watts-Strogatz model [30]: We denote every such network by $\mathit{SmallWorld}(n, d, p)$, where $n$ is the number of nodes, $d$ is the average degree, and $p$ is the rewiring probability;
- *Random graphs*, generated using the Erdos-Renyi model [7]: We denote every such network by $\mathit{RandomGraph}(n, d)$, with $n$ being the number of nodes, and $d$ being the expected average degree.

Next, we describe the real-life networks used in our experiments:

- Facebook [20]: we consider three fragments of Facebook's social network: (i) a "small" fragment consisting of 61 nodes and 272 edges; (ii) a "medium" fragment consisting of 333 nodes and 2,523 edges; and (iii) a "large" fragment consisting of 786 nodes and 14,027 edges;
- Madrid terrorist network [12]—the network of terrorists behind the 2004 Madrid bombing, consisting of 70 nodes and 98 edges;
- Bali terrorist network [12]—the network of terrorists behind the 2002 Bali attack, consisting of 17 nodes and 63 edges;
- WTC terrorist network [18]—the network of terrorists behind the 9/11 attacks, consisting of 36 nodes and 64 edges;
- Zachary's Karate Club [31]—the social network of participants of a university karate club, consisting of 34 nodes and 78 edges;
- Les Misérables [17]—the network of co-occurances of characters in Victor Hugo's novel "Les Misérables", consisting of 77 nodes and 254 edges;
- Greek blogs [32]—a network of Greek political blogs, consisting of 142 nodes and 354 edges;
- Telecommunication networks [23]—two fragments of a telecommunication network where the nodes correspond to the users of a particular European telecom operator and the links correspond to all the calls between those users: (i) a "small" fragment consisting of $56,073$ users who live in a single district in the UK and $174,608$ links between them, and (ii) a "large" fragment consisting of $248,763$ users who live in four geographically continuous districts and $829,725$ links between those users.

Next, we summarize the results for *local* similarity indices given all of these networks (Figure S5), before detailing the result for each network separately (Figures S6 to S10). After that, we do the same but for *global* similarity indices, i.e., we start by summarizing the results given all of the above networks (Figure S11), and then detail the result for each network separately (Figures S12 to S16).

### S7.2 Evaluating CTR and OTC Against Local Link Prediction Algorithms

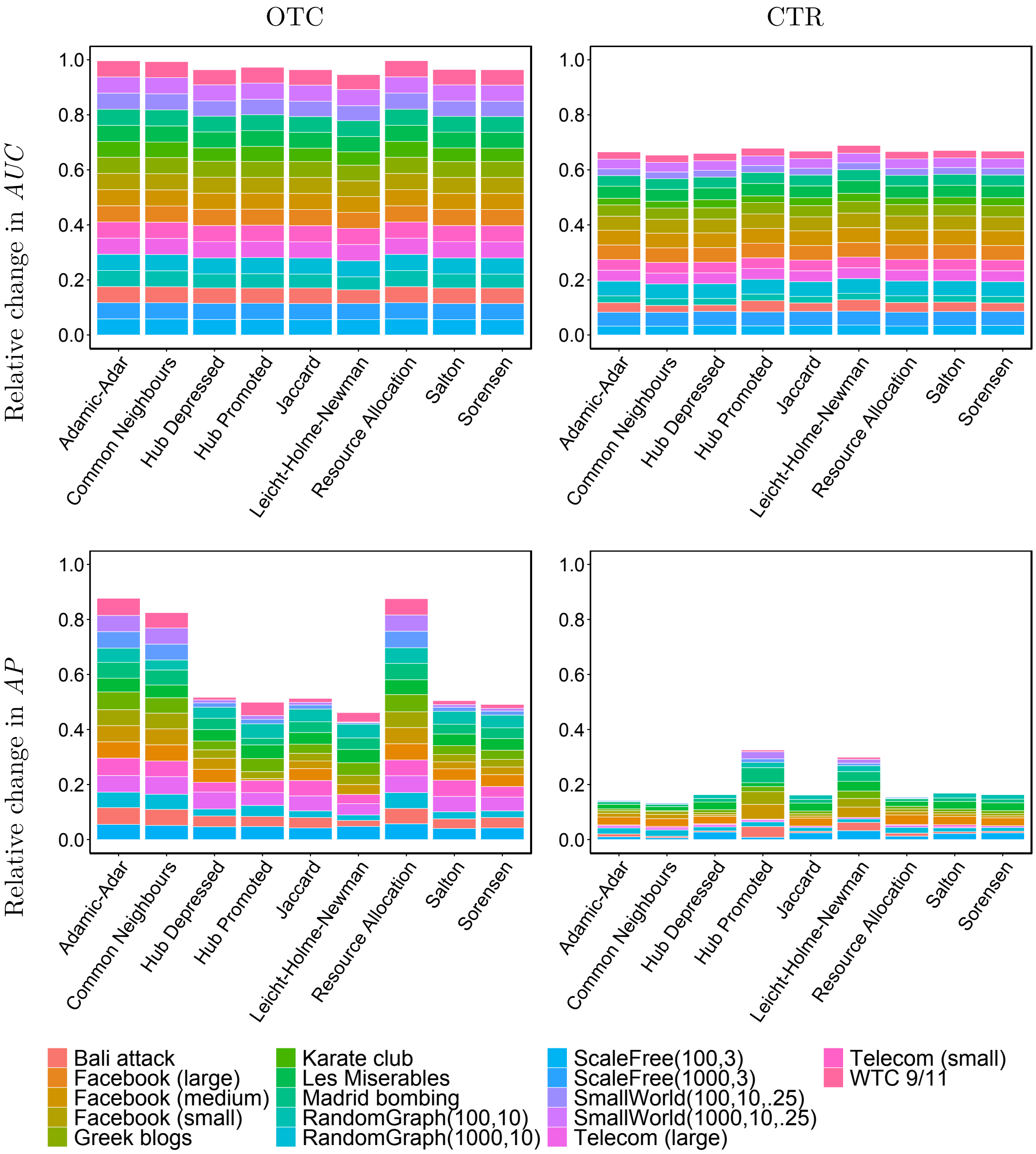


Figure S5: Given different **local similarity** indices, the figure depicts the relative change in $AUC$ (the area under the ROC curve) and $AP$ (the average precision) after running OTC and CTR in different networks. For each such network, we experiment with 10 different, randomly-chosen evaders that have at least 9 connections each (to ensures that no evader is entirely diconnected when running CTR), where $b = 5$ and $H$ consists of 3 edges chosen randomly from the evader's connections. The experiment is repeated 5 times for each evader. Moreover, for each model of random networks, the entire process is repeated 50 times, with a new network generated each time. In the bar plots, the height of the bar corresponding to each similarity index represents the average change taken over all networks, with the height of each segment in that bar being proportional to the change within the corresponding network. We disregard any $H$ for which the performance metric is below 0.001, and assume the edges therein to be hidden already.

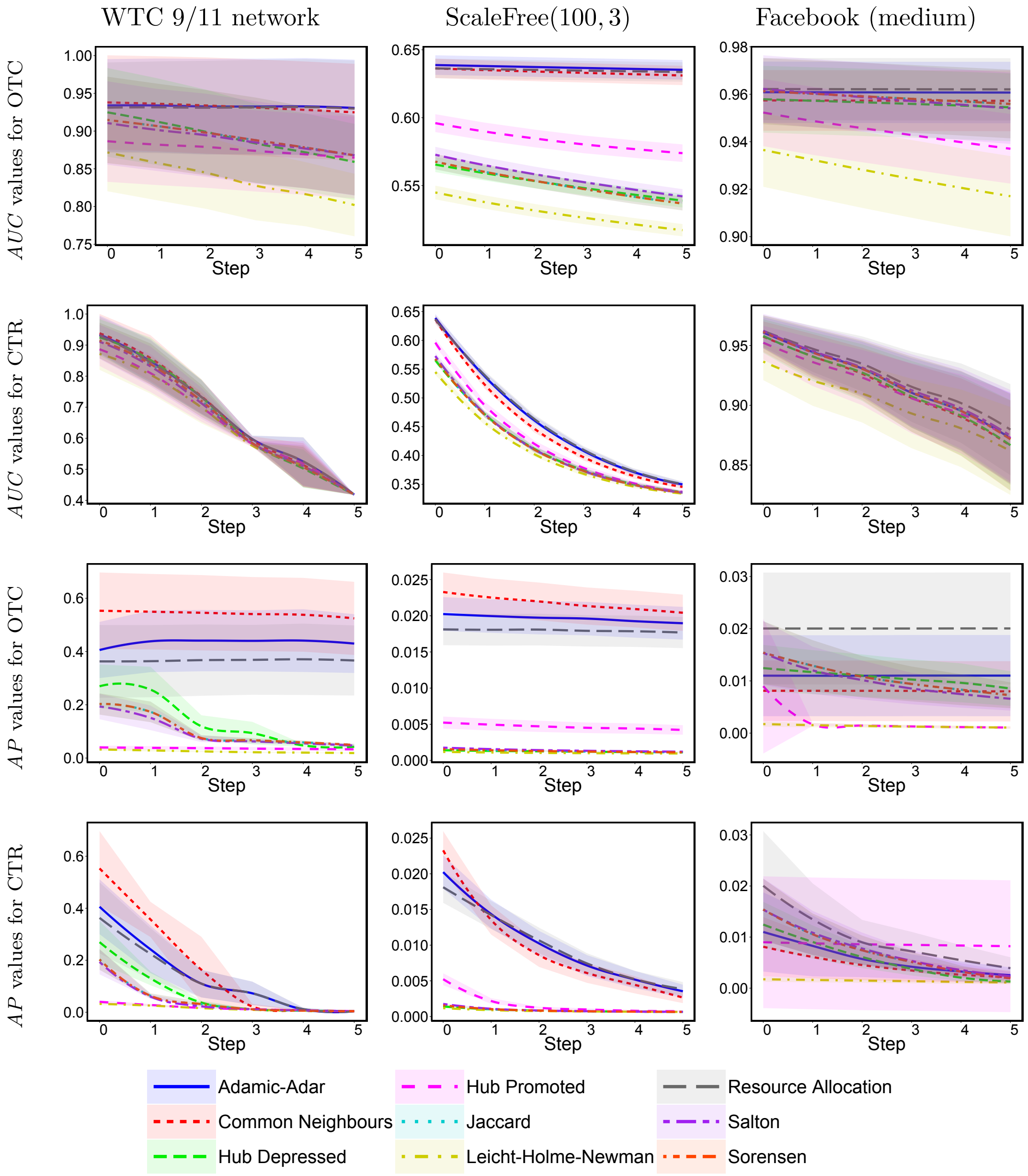


Figure S6: Given different **local similarity** indices, and three networks: (i) the **WTC 9/11 terrorist network**; (ii) **ScaleFree(100,3)**; and (iii) **a medium fragment of Facebook**, the figure depicts the relative change in $AUC$ and $AP$ during the execution of OTC and CTR by 10 different, randomly-chosen evaders that have at least 9 connections each (to ensures that no evader is entirely diconnected when running CTR), where $b = 5$ and $H$ consists of 3 edges chosen randomly from the evader's connections. The experiment is repeated 5 times for each evader. Moreover, for scale-free networks, the entire process is repeated 50 times, with a new network generated each time. Coloured areas represent 95% confidence intervals.

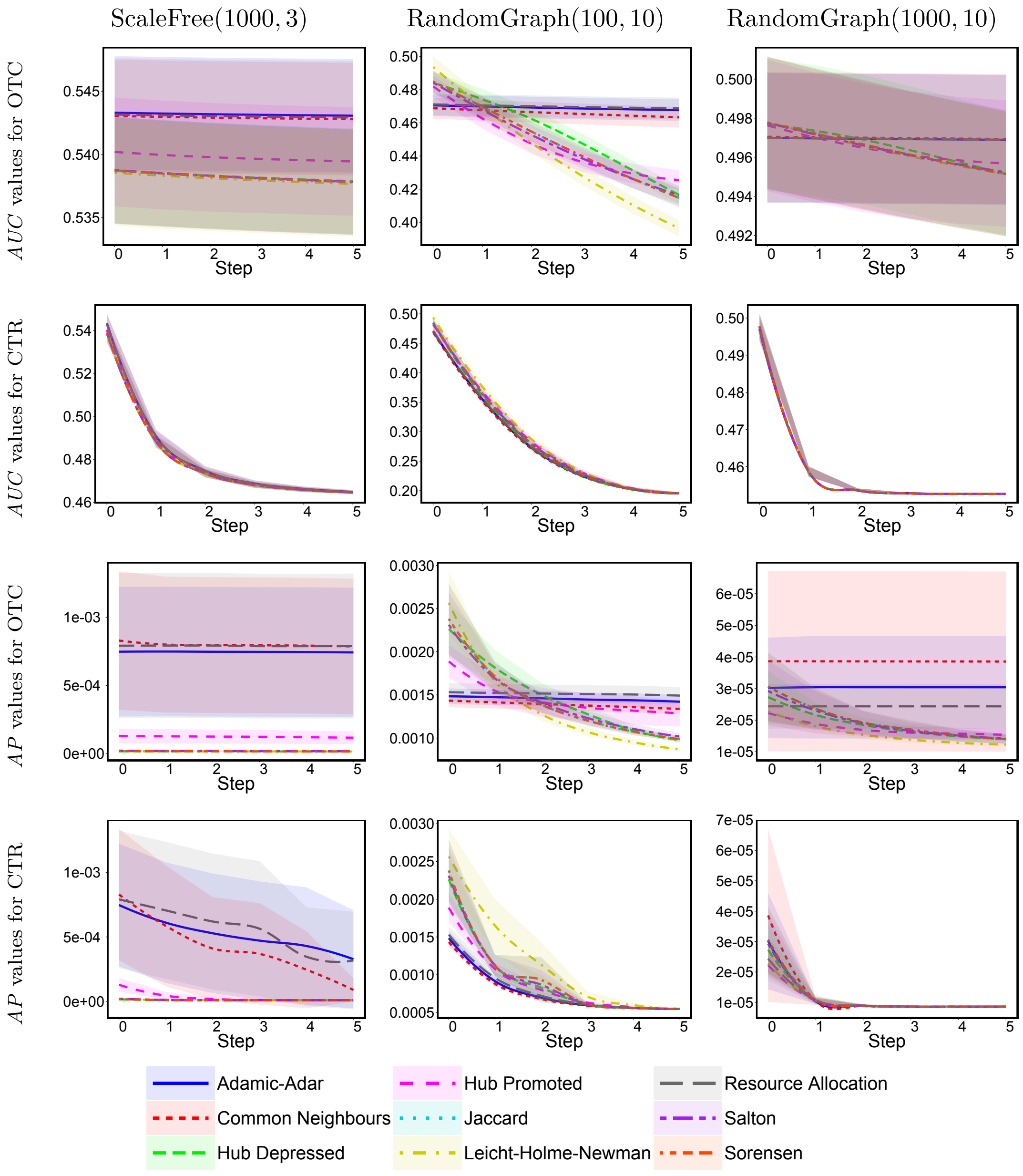


Figure S7: Given different **local similarity** indices, and three network generation models: (i) **Scale-Free(1000,3)**; (ii) **RandomGraph(100,10)**; and (iii) **RandomGraph(1000,10)**, the figure depicts the relative change in $AUC$ and $AP$ during the execution of OTC and CTR by 10 different, randomly-chosen evaders that have at least 9 connections each (to ensures that no evader is entirely diconnected when running CTR), where $b = 5$ and $H$ consists of 3 edges chosen randomly from the evader's connections. The experiment is repeated 5 times for each evader. Moreover, for each model of random networks, the entire process is repeated 50 times, with a new network generated each time. Coloured areas represent 95% confidence intervals.

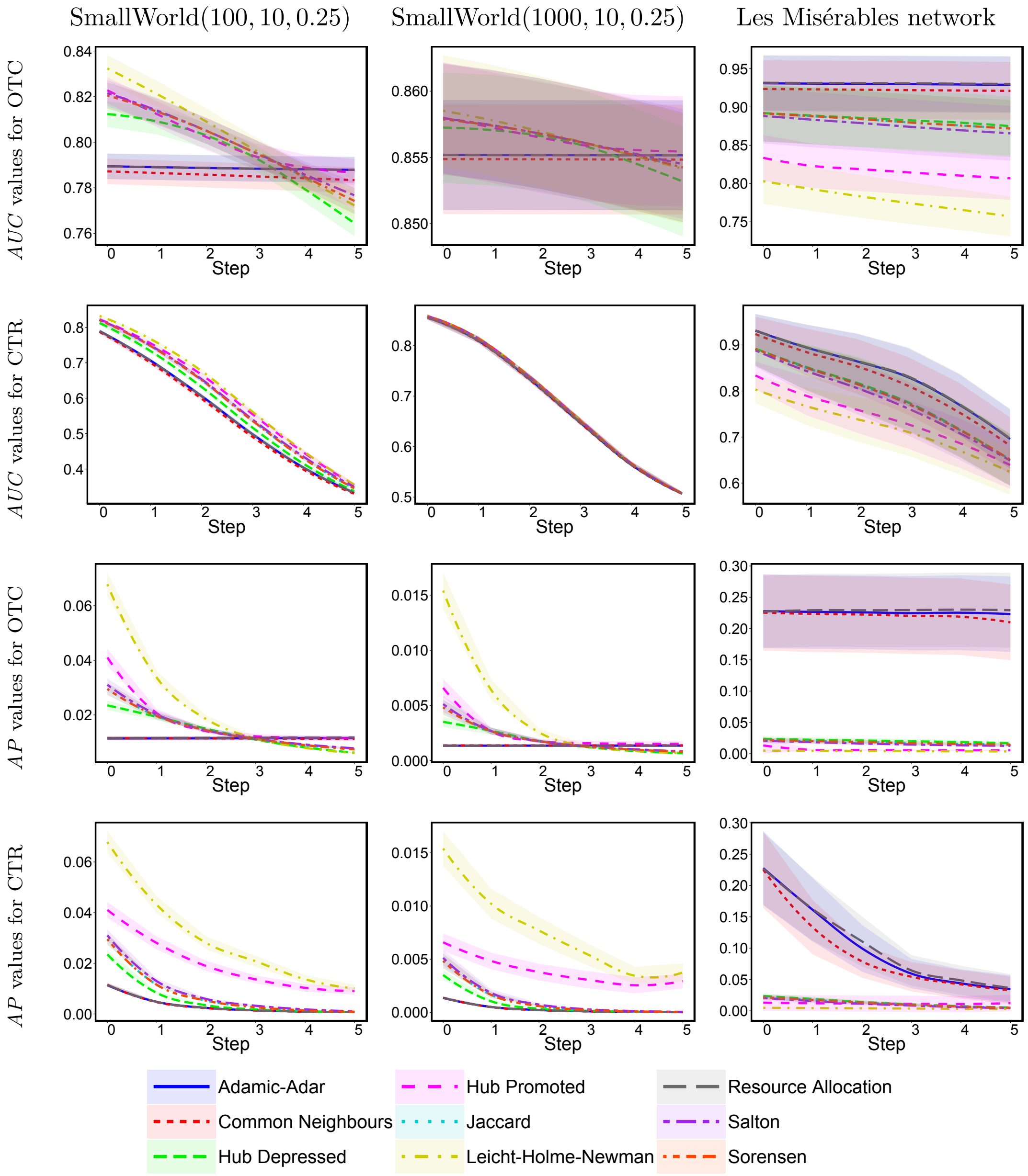


Figure S8: Given different **local similarity** indices, and three networks: (i) **SmallWorld(100,10,0.25)**; (ii) **SmallWorld(1000,10,0.25)**; and (iii) **Les Misérables network**, the figure depicts the relative change in $AUC$ and $AP$ during the execution of OTC and CTR by 10 different, randomly-chosen evaders that have at least 9 connections each (to ensures that no evader is entirely diconnected when running CTR), where $b = 5$ and $H$ consists of 3 edges chosen randomly from the evader's connections. The experiment is repeated 5 times for each evader. Moreover, for small-world networks, the entire process is repeated 50 times, with a new network generated each time. Coloured areas represent 95% confidence intervals.

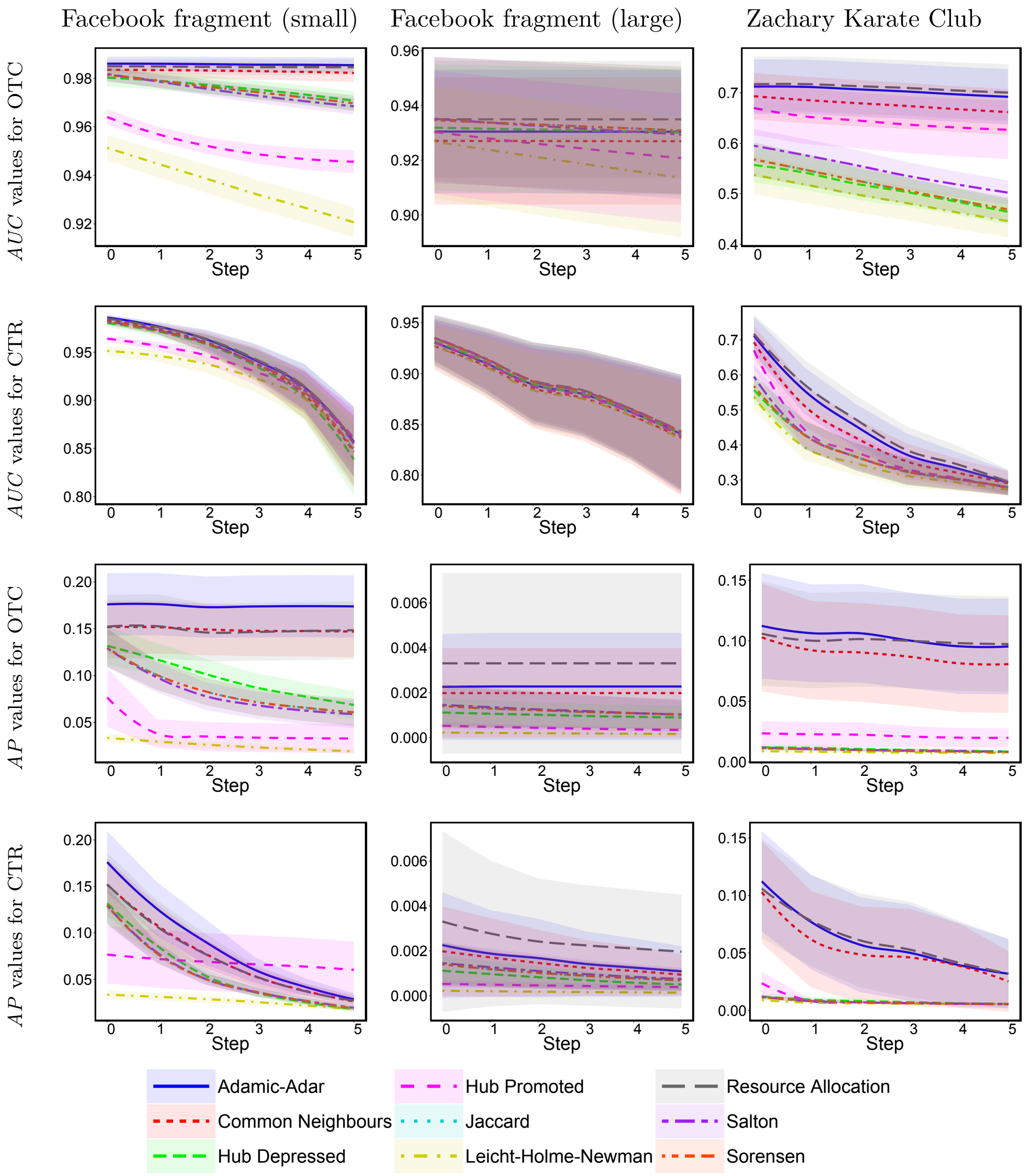


Figure S9: Given different **local similarity** indices, and three networks: (i) **a small fragment of Facebook**; (ii) **a large fragment of Facebook**; and (iii) **the Zachary karate club network**, the figure depicts the relative change in *AUC* and *AP* during the execution of OTC and CTR by 10 different, randomly-chosen evaders that have at least 9 connections each (to ensures that no evader is entirely diconnected when running CTR), where $b = 5$ and $H$ consists of 3 edges chosen randomly from the evader's connections. The experiment is repeated 5 times for each evader. Coloured areas represent 95% confidence intervals.

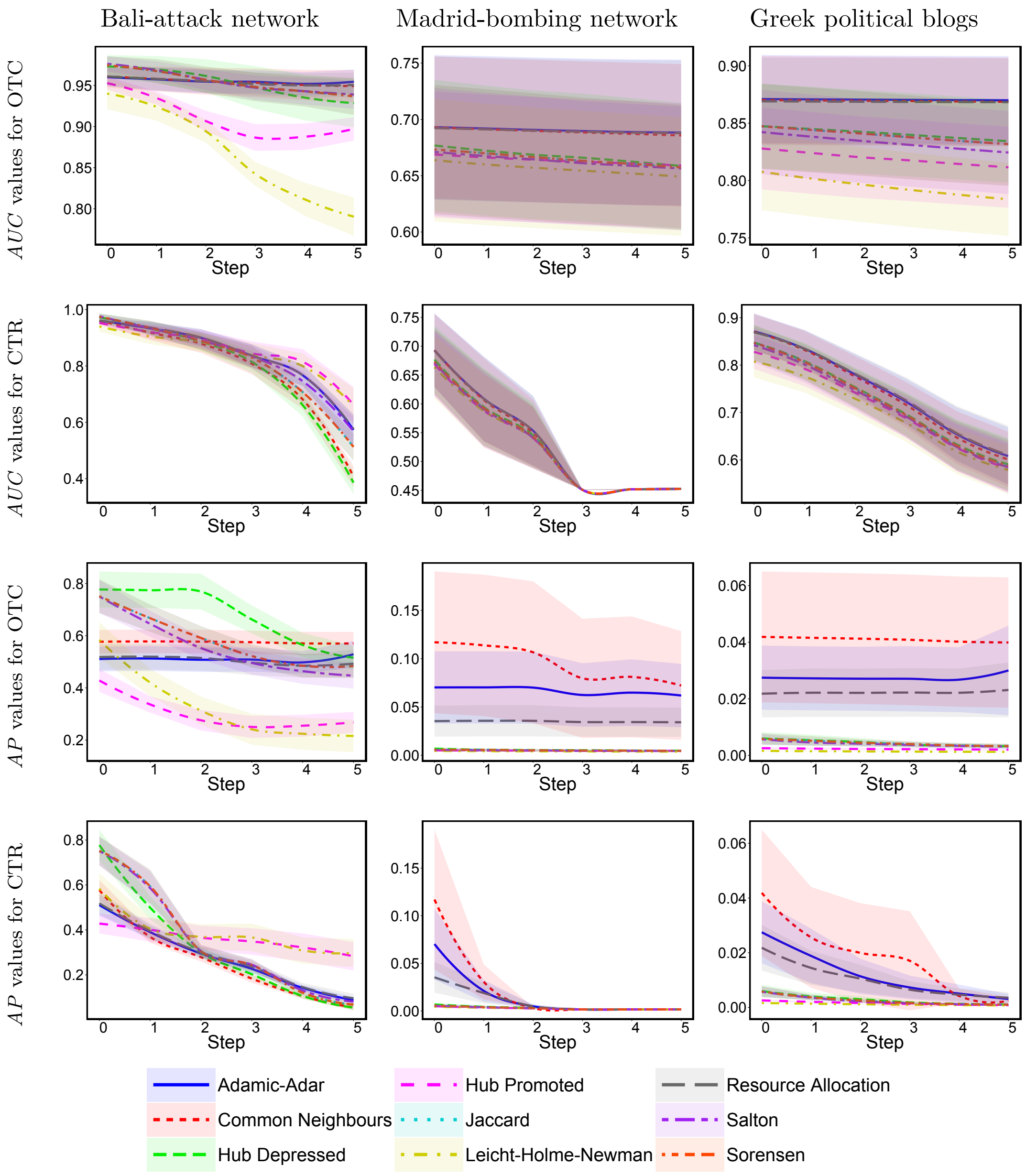


Figure S10: Given different **local similarity** indices, and three networks: (i) **the Bali-attack network**; (ii) **the Madrid-bombing network**; and (iii) **the Greek political blog network**, the figure depicts the relative change in $AUC$ and $AP$ during the execution of OTC and CTR by 10 different, randomly-chosen evaders that have at least 9 connections each (to ensures that no evader is entirely diconnected when running CTR), where $b = 5$ and $H$ consists of 3 edges chosen randomly from the evader's connections. The experiment is repeated 5 times for each evader. Coloured areas represent 95% confidence intervals.

## S7.3 Evaluating CTR and OTC Against Global Link Prediction Algorithms

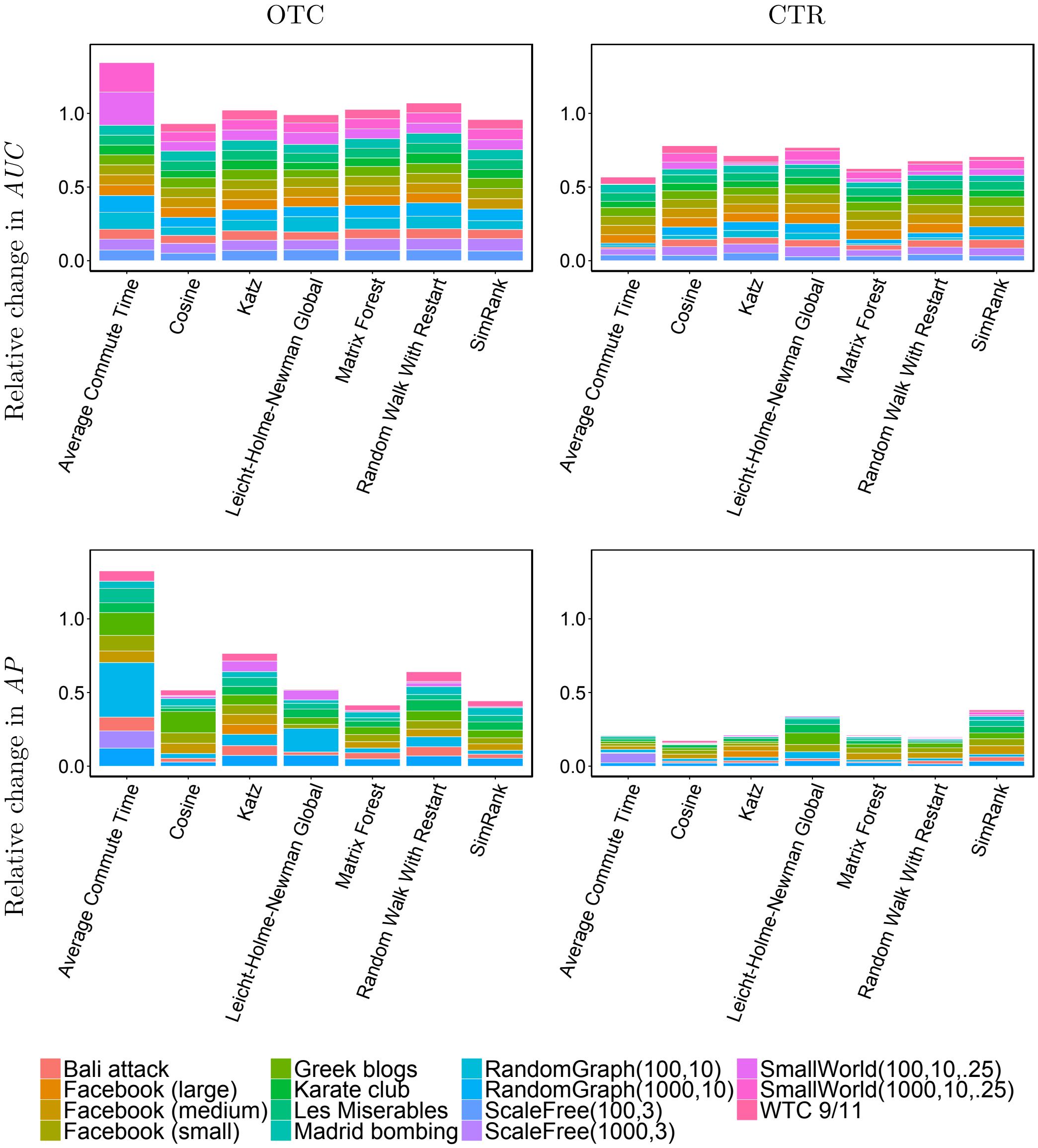


Figure S11: Given different **global similarity** indices, the figure depicts the relative change in $AUC$ (the area under the ROC curve) and $AP$ (the average precision) after running OTC and CTR in different networks. For each such network, we experiment with 10 different, randomly-chosen evaders that have at least 9 connections each (to ensures that no evader is entirely diconnected when running CTR), where $b = 5$ and $H$ consists of 3 edges chosen randomly from the evader's connections. The experiment is repeated 5 times for each evader. Moreover, for each model of random networks, the entire process is repeated 50 times, with a new network generated each time. In the bar plots, the height of the bar corresponding to each similarity index represents the average change taken over all networks, with the height of each segment in that bar being proportional to the change within the corresponding network. We disregard any $H$ for which the performance metric is below 0.001, and assume the edges therein to be hidden already.

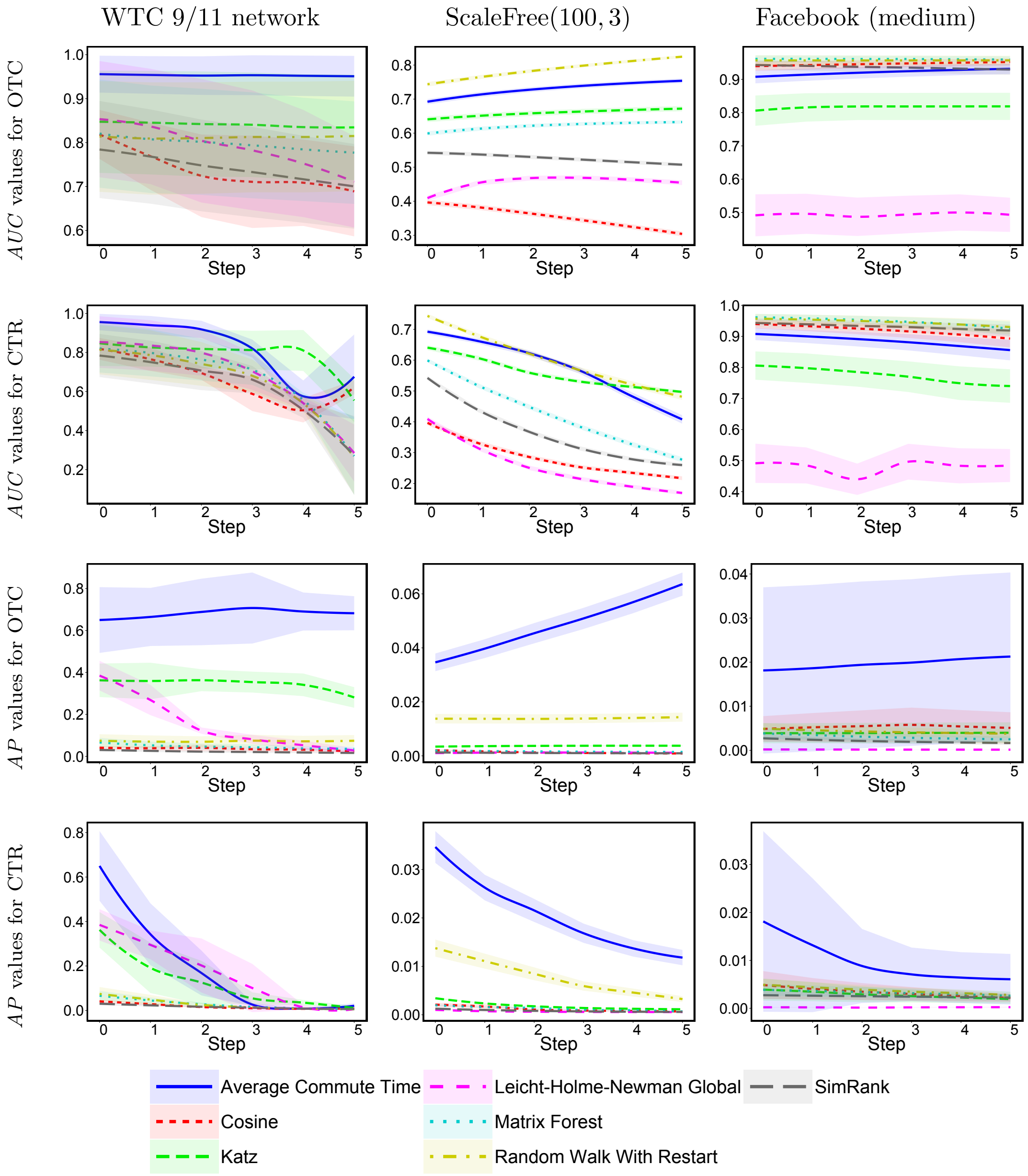


Figure S12: Given different **global similarity** indices, and three networks: (i) the **WTC 9/11 terrorist network**; (ii) **ScaleFree(100,3)**; and (iii) **a medium fragment of Facebook**, the figure depicts the relative change in *AUC* and *AP* during the execution of OTC and CTR by 10 different, randomly-chosen evaders that have at least 9 connections each (to ensures that no evader is entirely diconnected when running CTR), where $b = 5$ and $H$ consists of 3 edges chosen randomly from the evader's connections. The experiment is repeated 5 times for each evader. Moreover, for scale-free networks, the entire process is repeated 50 times, with a new network generated each time. Coloured areas represent 95% confidence intervals.

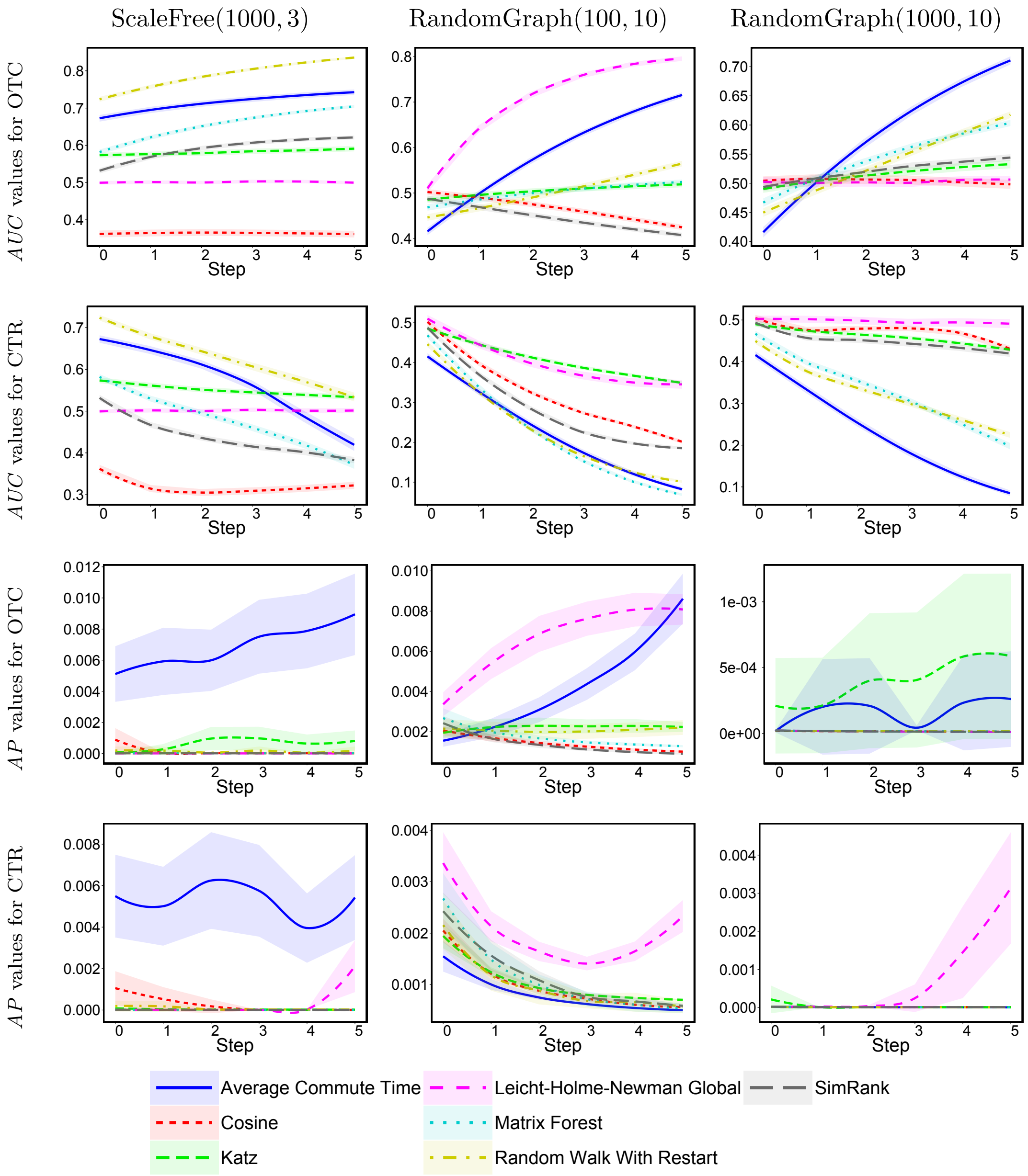


Figure S13: Given different **global similarity** indices, and three network generation models: (i) **Scale-Free(1000,3)**; (ii) **RandomGraph(100,10)**; and (iii) **RandomGraph(1000,10)**, the figure depicts the relative change in $AUC$ and $AP$ during the execution of OTC and CTR by 10 different, randomly-chosen evaders that have at least 9 connections each (to ensures that no evader is entirely diconnected when running CTR), where $b = 5$ and $H$ consists of 3 edges chosen randomly from the evader's connections. The experiment is repeated 5 times for each evader. Moreover, for each model of random networks, the entire process is repeated 50 times, with a new network generated each time. Coloured areas represent 95% confidence intervals.

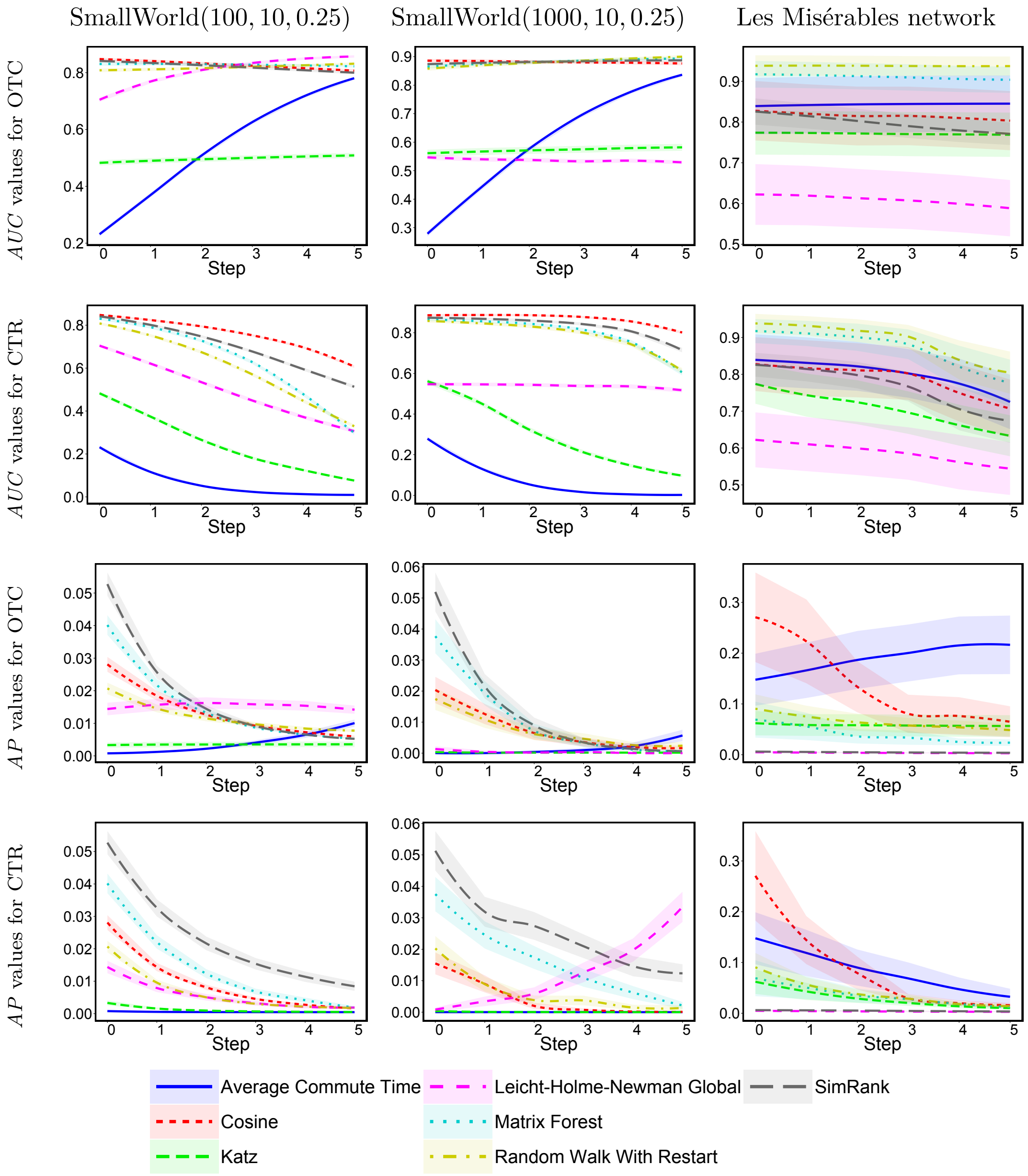


Figure S14: Given different **global similarity** indices, and three networks: (i) **SmallWorld(100,10,0.25)**; (ii) **SmallWorld(1000,10,0.25)**; and (iii) **Les Misérables network**, the figure depicts the relative change in $AUC$ and $AP$ during the execution of OTC and CTR by 10 different, randomly-chosen evaders that have at least 9 connections each (to ensures that no evader is entirely diconnected when running CTR), where $b = 5$ and $H$ consists of 3 edges chosen randomly from the evader's connections. The experiment is repeated 5 times for each evader. Moreover, for small-world networks, the entire process is repeated 50 times, with a new network generated each time. Coloured areas represent 95% confidence intervals.

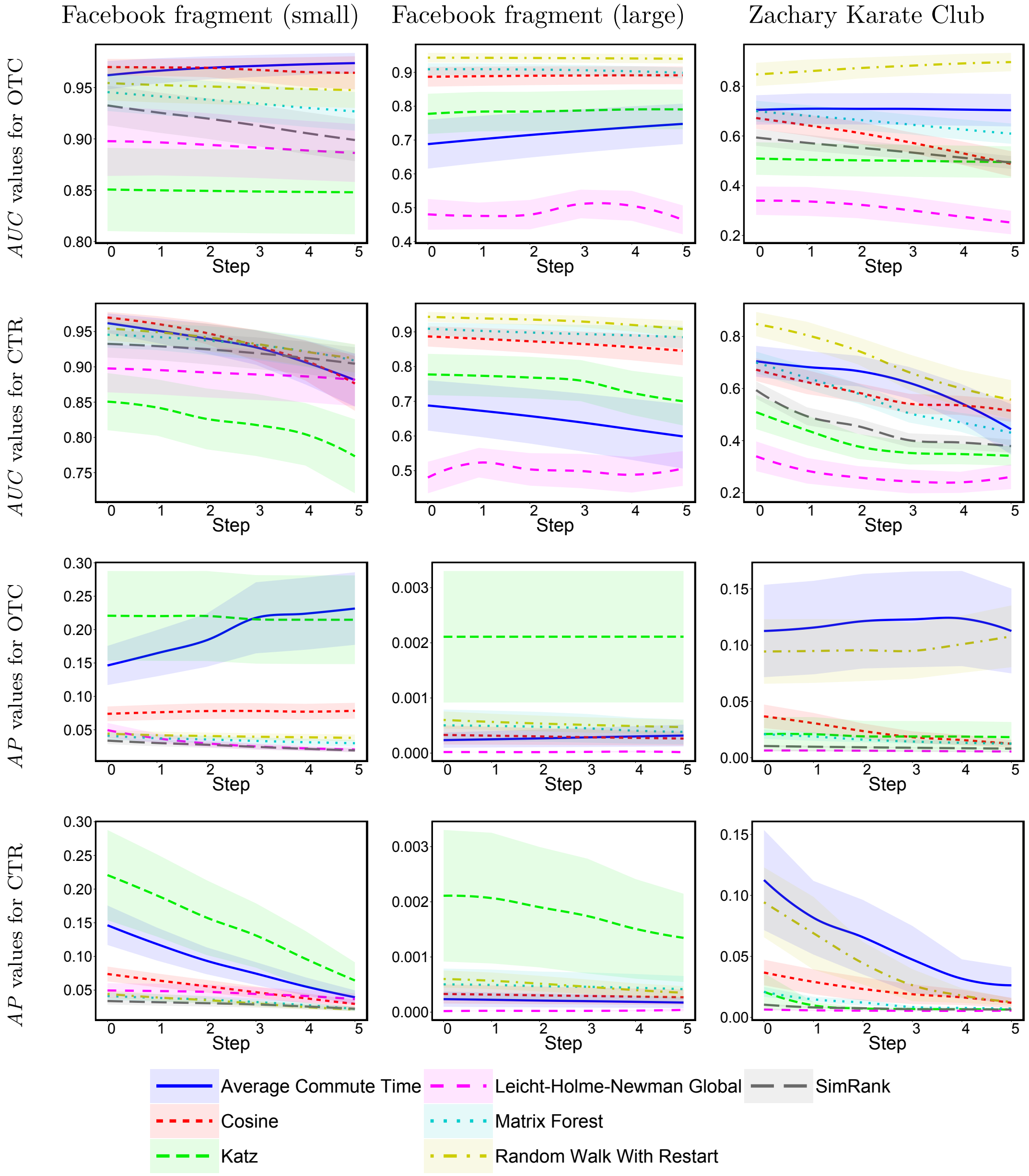


Figure S15: Given different **global similarity** indices, and three networks: (i) **a small fragment of Facebook**; (ii) **a large fragment of Facebook**; and (iii) **the Zachary karate club network**, the figure depicts the relative change in $AUC$ and $AP$ during the execution of OTC and CTR by 10 different, randomly-chosen evaders that have at least 9 connections each (to ensures that no evader is entirely diconnected when running CTR), where $b = 5$ and $H$ consists of 3 edges chosen randomly from the evader's connections. The experiment is repeated 5 times for each evader. Coloured areas represent 95% confidence intervals.

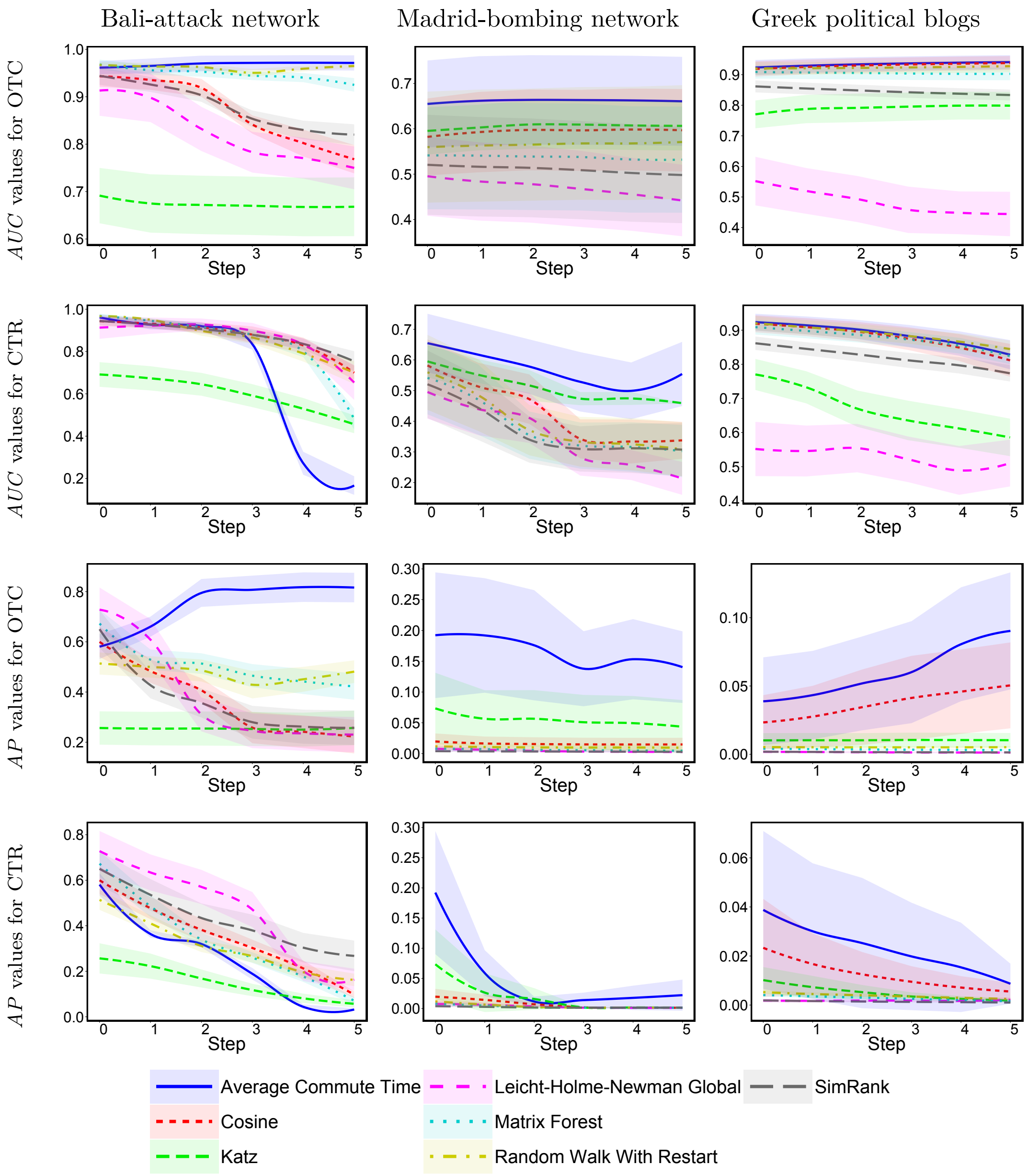


Figure S16: Given different **global similarity** indices, and three networks: (i) **the Bali-attack network**; (ii) **the Madrid-bombing network**; and (iii) **the Greek political blog network**, the figure depicts the relative change in $AUC$ and $AP$ during the execution of OTC and CTR by 10 different, randomly-chosen evaders that have at least 9 connections each (to ensures that no evader is entirely diconnected when running CTR), where $b = 5$ and $H$ consists of 3 edges chosen randomly from the evader's connections. The experiment is repeated 5 times for each evader. Coloured areas represent 95% confidence intervals.

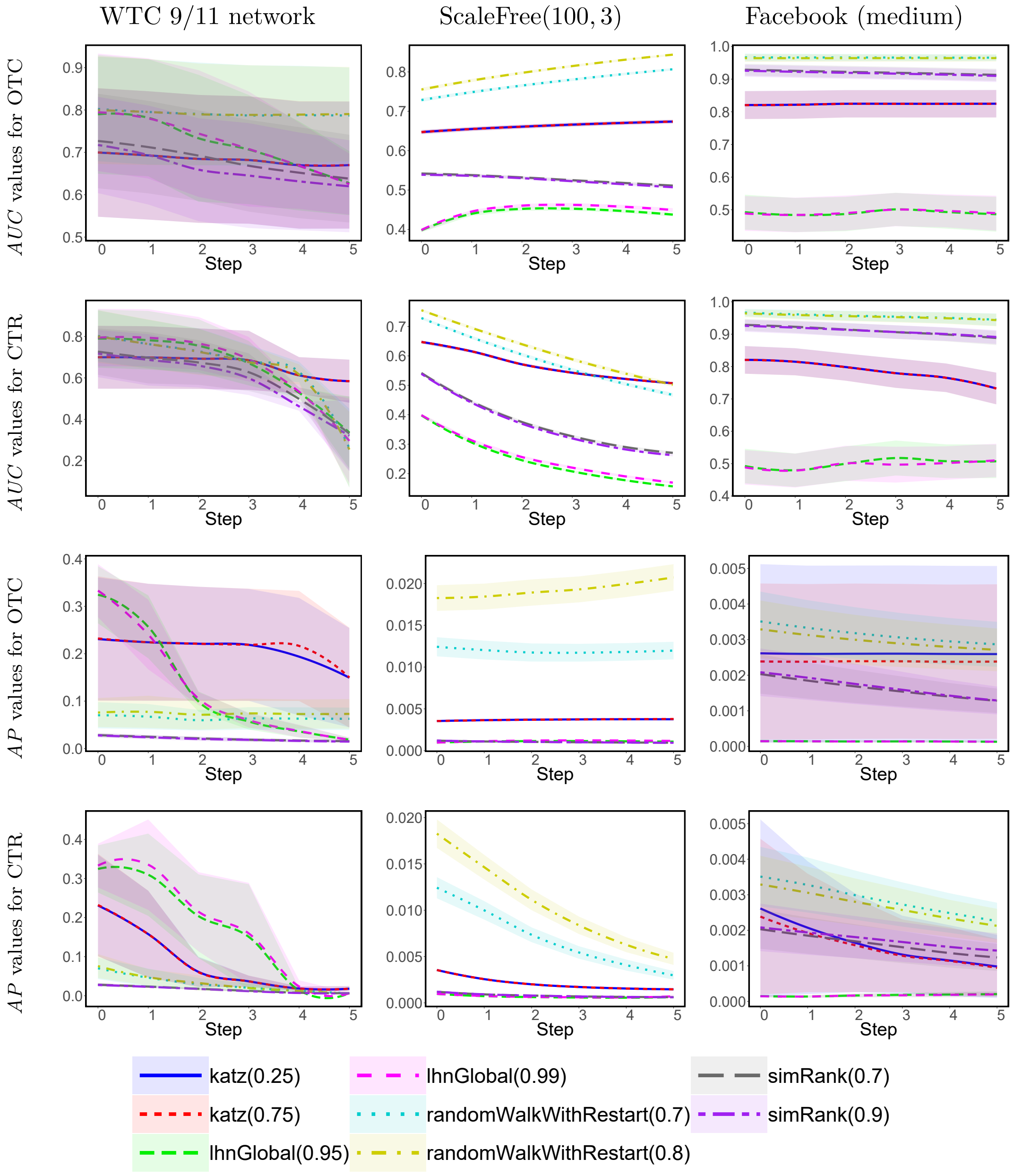


Figure S17: **Comparison of different paramterizations of global similarity indices.** Given different **global similarity** indices, and three networks: (i) the **WTC 9/11 terrorist network**; (ii) **Scale-Free(100,3)**; and (iii) **a medium fragment of Facebook**, the figure depicts the relative change in $AUC$ and $AP$ during the execution of OTC and CTR by 10 different, randomly-chosen evaders that have at least 9 connections each (to ensures that no evader is entirely diconnected when running CTR), where $b = 5$ and $H$ consists of 3 edges chosen randomly from the evader's connections. The experiment is repeated 5 times for each evader. Moreover, for scale-free networks, the entire process is repeated 50 times, with a new network generated each time. Coloured areas represent 95% confidence intervals.

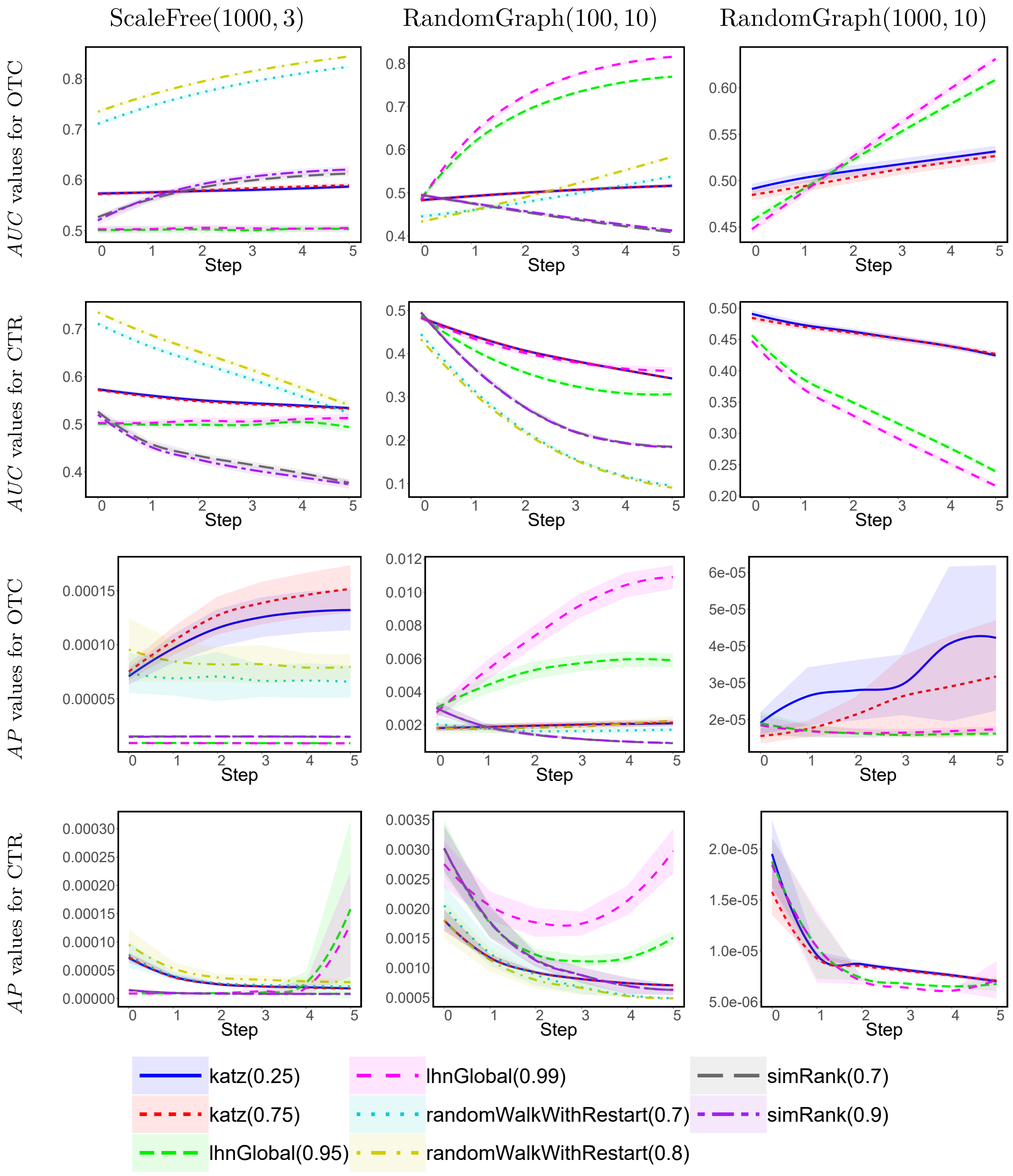


Figure S18: **Comparison of different paramterizations of global similarity indices.** Given different **global similarity** indices, and three network generation models: (i) **ScaleFree(1000,3)**; (ii) **RandomGraph(100,10)**; and (iii) **RandomGraph(1000,10)**, the figure depicts the relative change in $AUC$ and $AP$ during the execution of OTC and CTR by 10 different, randomly-chosen evaders that have at least 9 connections each (to ensures that no evader is entirely diconnected when running CTR), where $b = 5$ and $H$ consists of 3 edges chosen randomly from the evader's connections. The experiment is repeated 5 times for each evader. Moreover, for each model of random networks, the entire process is repeated 50 times, with a new network generated each time. Coloured areas represent 95% confidence intervals.

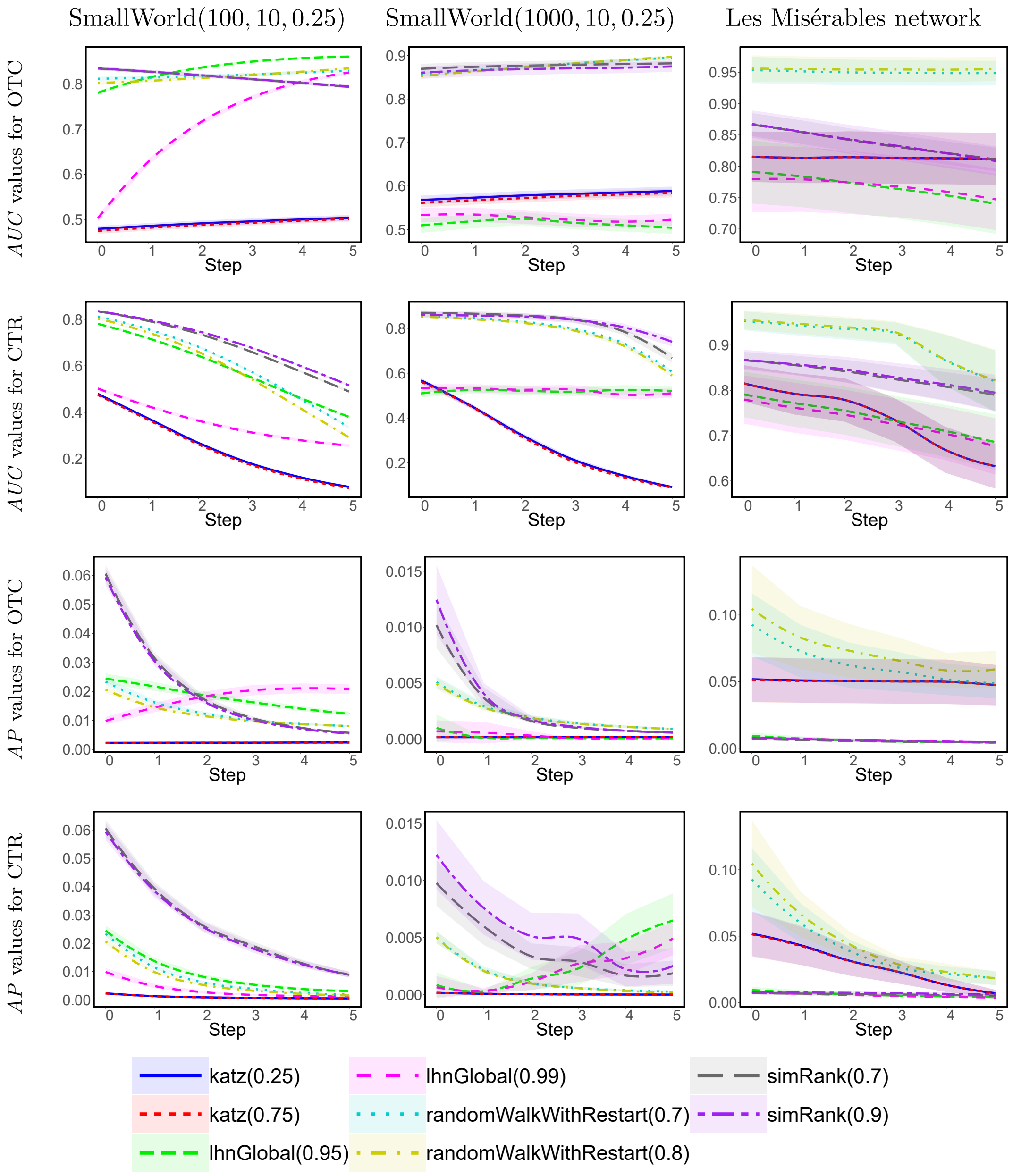


Figure S19: **Comparison of different paramterizations of global similarity indices.** Given different **global similarity** indices, and three networks: (i) **SmallWorld(100,10,0.25)**; (ii) **SmallWorld(1000,10,0.25)**; and (iii) **Les Misérables network**, the figure depicts the relative change in $AUC$ and $AP$ during the execution of OTC and CTR by 10 different, randomly-chosen evaders that have at least 9 connections each (to ensures that no evader is entirely diconnected when running CTR), where $b = 5$ and $H$ consists of 3 edges chosen randomly from the evader's connections. The experiment is repeated 5 times for each evader. Moreover, for small-world networks, the entire process is repeated 50 times, with a new network generated each time. Coloured areas represent 95% confidence intervals.

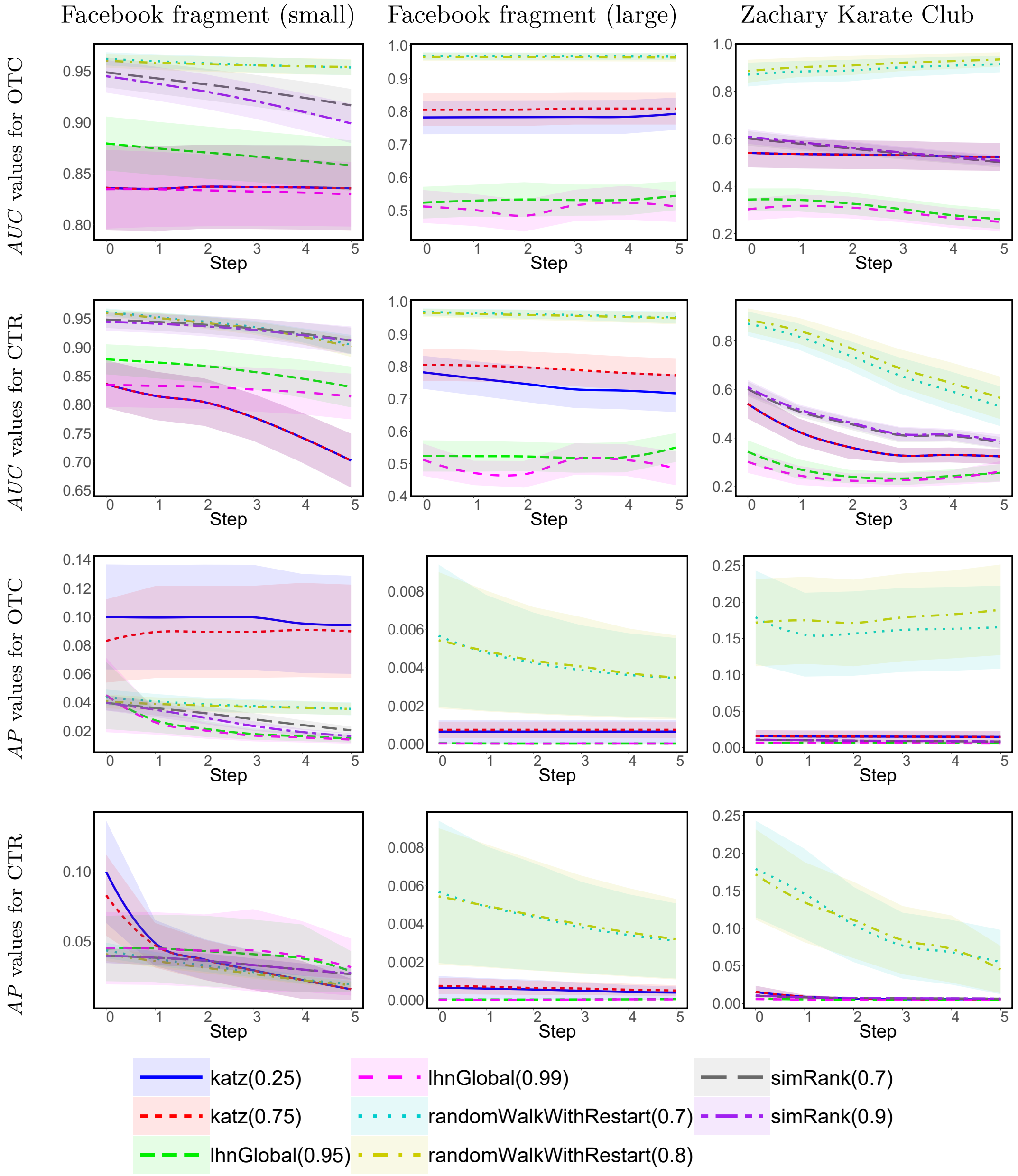


Figure S20: **Comparison of different paramterizations of global similarity indices.** Given different **global similarity** indices, and three networks: (i) **a small fragment of Facebook**; (ii) **a large fragment of Facebook**; and (iii) **the Zachary karate club network**, the figure depicts the relative change in $AUC$ and $AP$ during the execution of OTC and CTR by 10 different, randomly-chosen evaders that have at least 9 connections each (to ensures that no evader is entirely diconnected when running CTR), where $b = 5$ and $H$ consists of 3 edges chosen randomly from the evader's connections. The experiment is repeated 5 times for each evader. Coloured areas represent 95% confidence intervals.

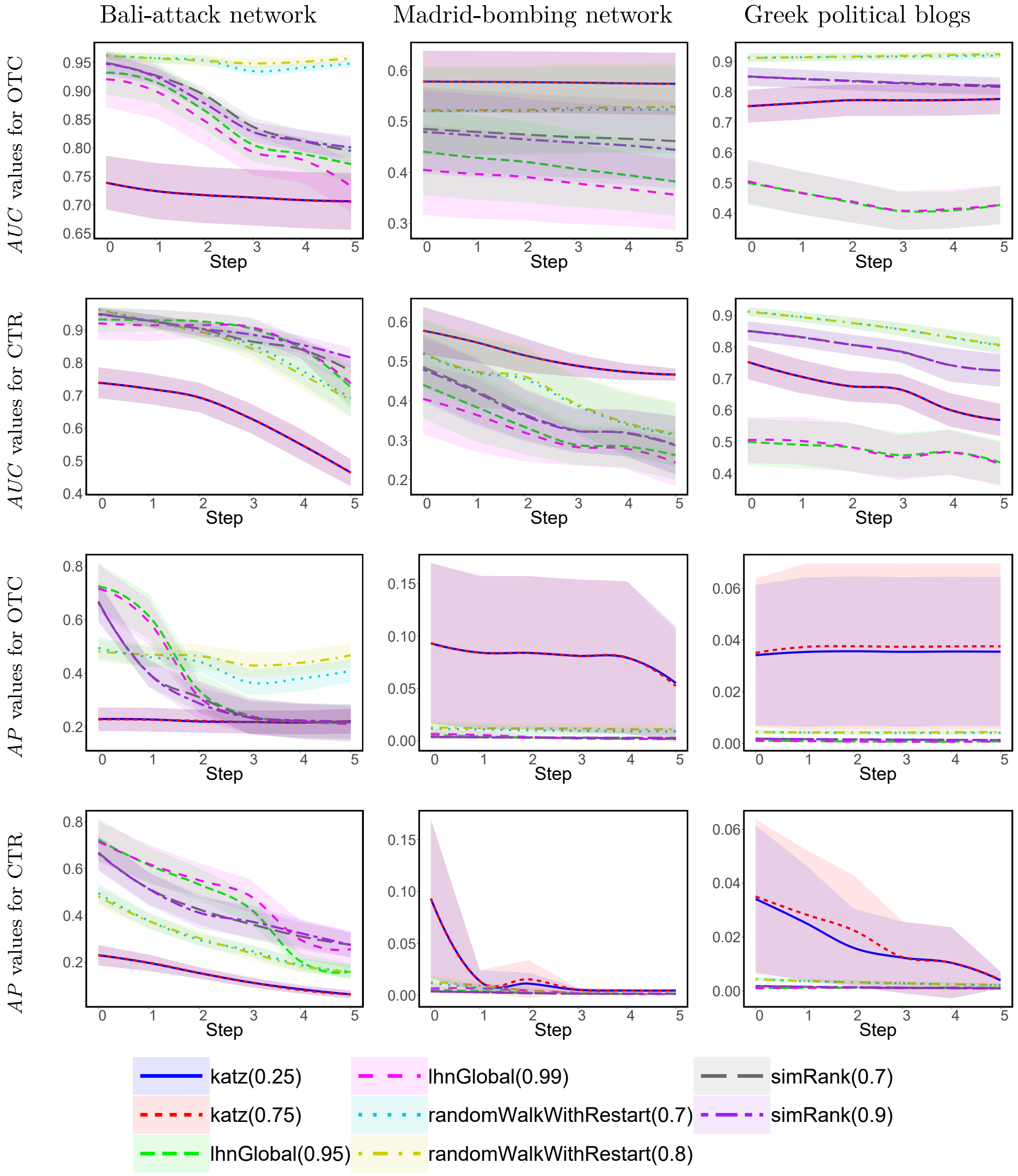


Figure S21: **Comparison of different paramterizations of global similarity indices.** Given different **global similarity** indices, and three networks: (i) **the Bali-attack network**; (ii) **the Madrid-bombing network**; and (iii) **the Greek political blog network**, the figure depicts the relative change in $AUC$ and $AP$ during the execution of OTC and CTR by 10 different, randomly-chosen evaders that have at least 9 connections each (to ensures that no evader is entirely diconnected when running CTR), where $b = 5$ and $H$ consists of 3 edges chosen randomly from the evader's connections. The experiment is repeated 5 times for each evader. Coloured areas represent 95% confidence intervals.

## S7.4 Hiding in Large-Scale Real-Life Networks

In the main article, we evaluated OCT and CTR in a telecommunication network consisting of 248,763 nodes and 829,725 edges. The nodes of that network corresponded to all the users of a particular European telecom operator, who live in four geographically continuous districts in the UK, and the links corresponded to all the calls between those users (see Figure 3 in the main article). In this section, we consider a smaller telecommunication network consisting of 56,073 nodes and 174,608 links, where the nodes correspond to all users living in just a single district in the UK, and the links correspond to all the calls between those users. The results depicted in Figure S22 exhibit similar trends to those presented in Figure 3, i.e., CTR is effective in terms of both $AP$ and $AUC$, while OTC is less effective in terms of $AP$ and not effective at all in terms of $AUC$; mixing the two heuristics does not seem to produce any synergistic effects.

To validate our results, we also provide results for two alternative large-scale networks. The first one is Enron email network [16] consisting of 36,692 nodes and 183,831 edges. Each node corresponds to a person considered in the Enron scandal investigation, with two people connected with an edge if they exchanged an email. The other network is another fragment of Facebook [29] consisting of 63,731 nodes and 817,035 edges. The results for these two networks are presented in Figures S23 and S24, and they show similar trends to the results for the telecommunication networks.

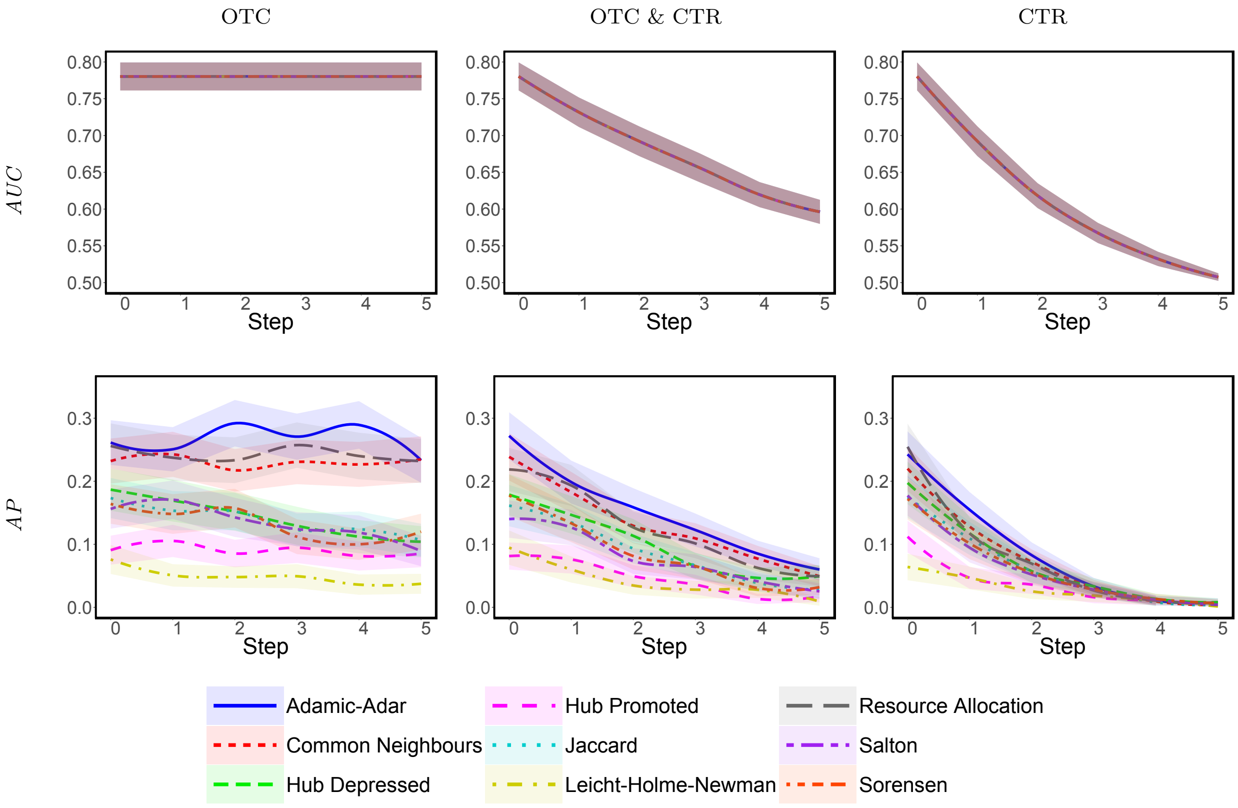


Figure S22: Given different local similarity indices, and a telecommunication network consisting of 56,073 nodes and 174,608 edges, the figure depicts the average $AUC$ and $AP$ during the execution of OTC and CTR with a budget $b = 5$. For each similarity index, we consider 10 different, randomly-chosen evaders that have at least 9 connections each (to ensures that no evader is entirely diconnected when running CTR); $H$ consists of 3 edges chosen randomly from the evader's connections. The experiment is repeated 5 times for each evader, and the average results are reported with the coloured areas representing the 95% confidence intervals.

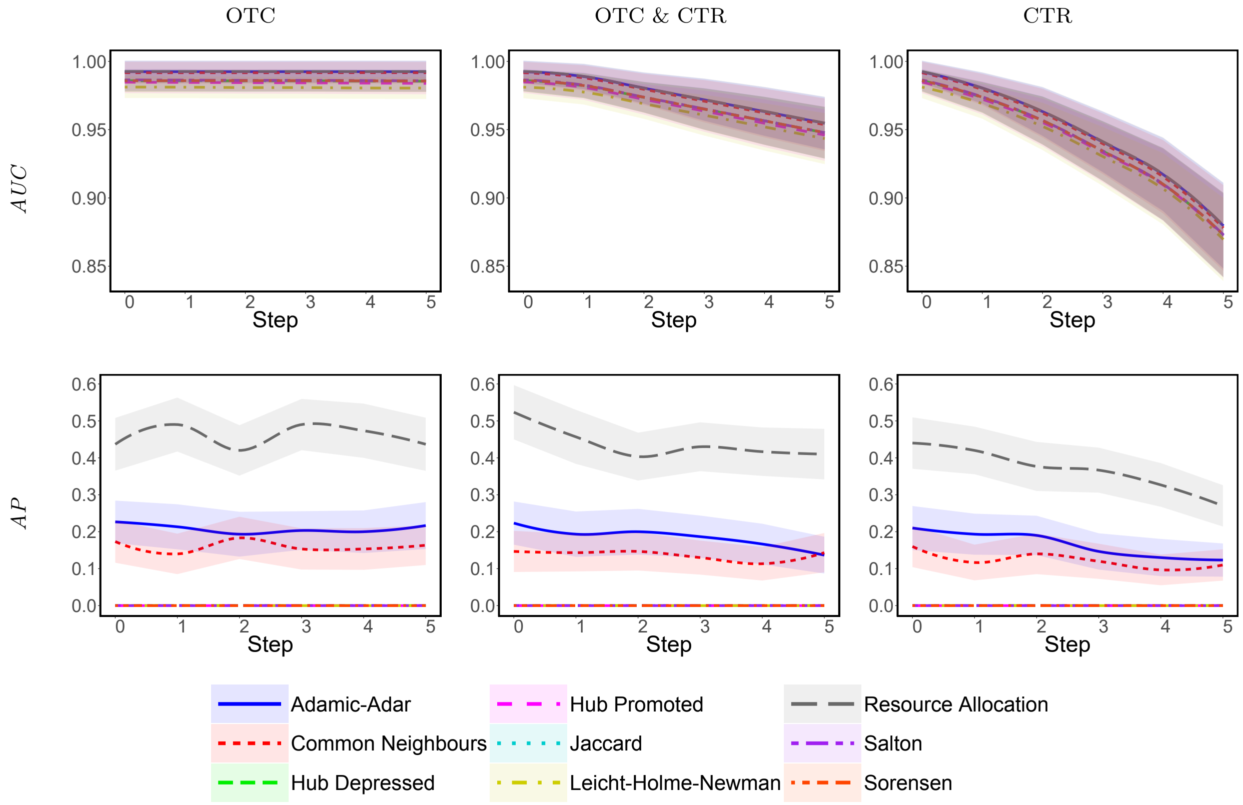


Figure S23: Given different local similarity indices, and an Enron email network consisting of 36, 692 nodes and 183, 831 edges, the figure depicts the average $AUC$ and $AP$ during the execution of OTC and CTR with a budget $b = 5$. For each similarity index, we consider 10 different, randomly-chosen evaders that have at least 9 connections each (to ensures that no evader is entirely diconnected when running CTR); $H$ consists of 3 edges chosen randomly from the evader's connections. The experiment is repeated 5 times for each evader, and the average results are reported with the coloured areas representing the 95% confidence intervals.

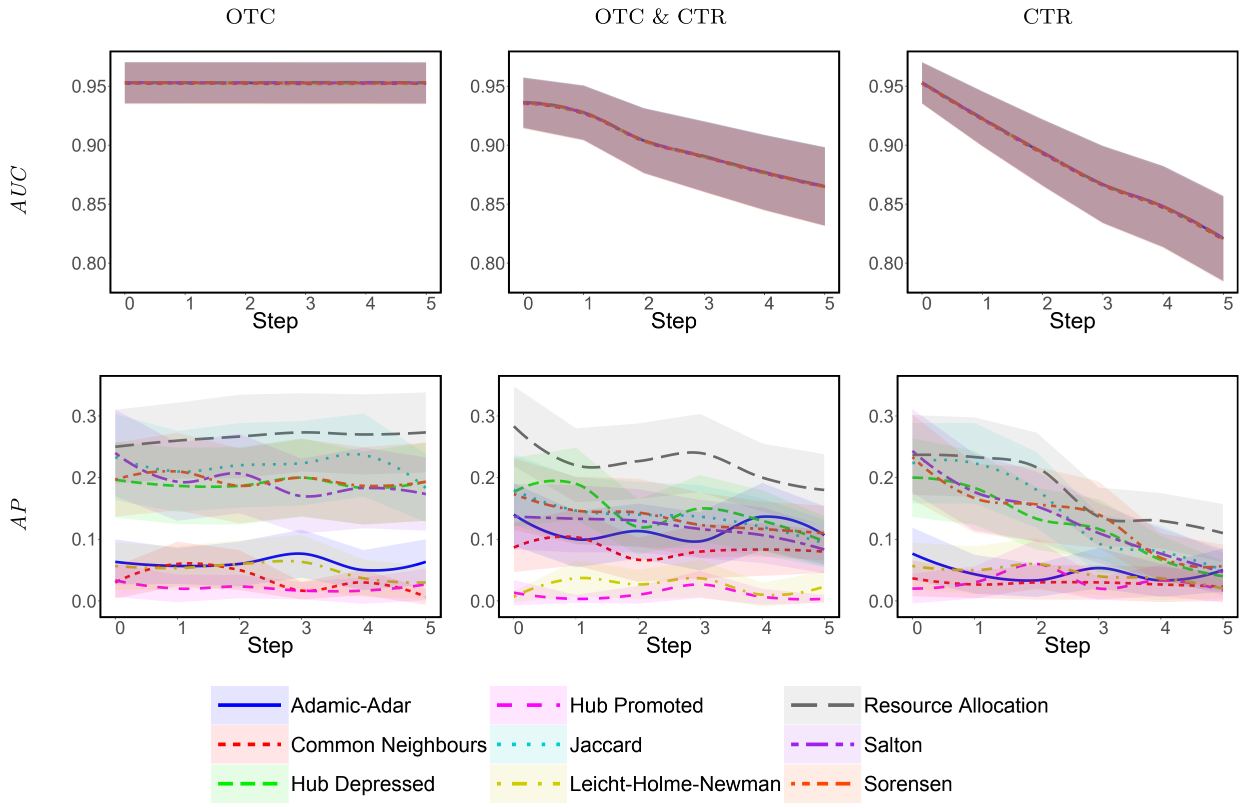


Figure S24: Given different local similarity indices, and a very large Facebook fragment consisting of 63,731 nodes and 817,035 edges, the figure depicts the average $AUC$ and $AP$ during the execution of OTC and CTR with a budget $b = 5$. For each similarity index, we consider 10 different, randomly-chosen evaders that have at least 9 connections each (to ensures that no evader is entirely diconnected when running CTR); $H$ consists of 3 edges chosen randomly from the evader's connections. The experiment is repeated 5 times for each evader, and the average results are reported with the coloured areas representing the 95% confidence intervals.

## S8 Evaluating Attack Tolerance of Link Prediction Algorithms

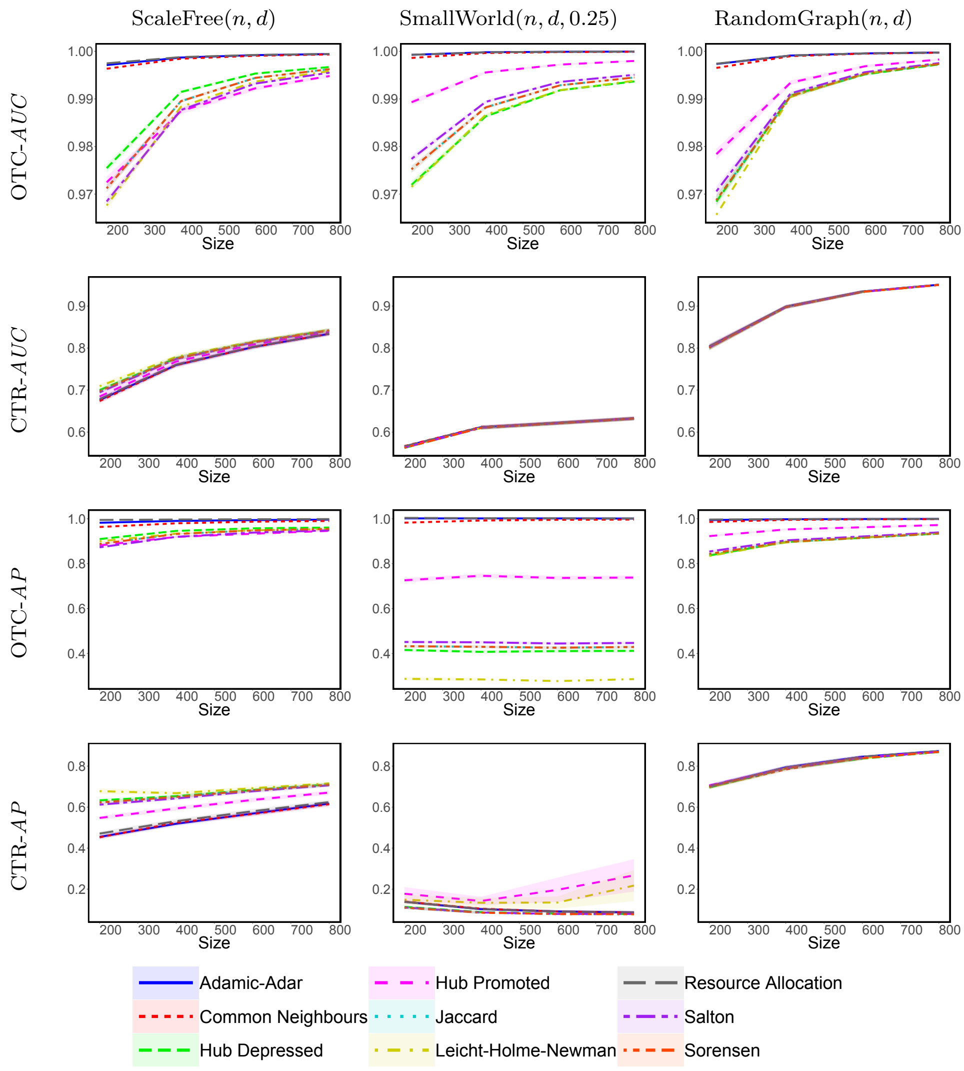


Figure S25: Evaluating the attack tolerance of the local similarity indices against OTC and CTR by measuring the relative change in $AUC$ and $AP$ while varying the **number of nodes**, $n$, in three types of networks: (i) ScaleFree($n, d$); (ii) SmallWorld($n, d, 0.25$); and (iii) RandomGraph($n, d$). For each $n$, we report the average over $d = 4, 6, 8, 10$. For any given $n$ and $d$, we run 50 experiments, each with 10 randomly-chosen evaders, none of which have less than 9 connections (to ensures that no evader is entirely diconnected when running CTR), where $b = 5$ and $H$ consists of 3 edges chosen randomly from the evader's connections. The experiment is repeated 5 times for each evader. Coloured areas represent 95% confidence intervals.

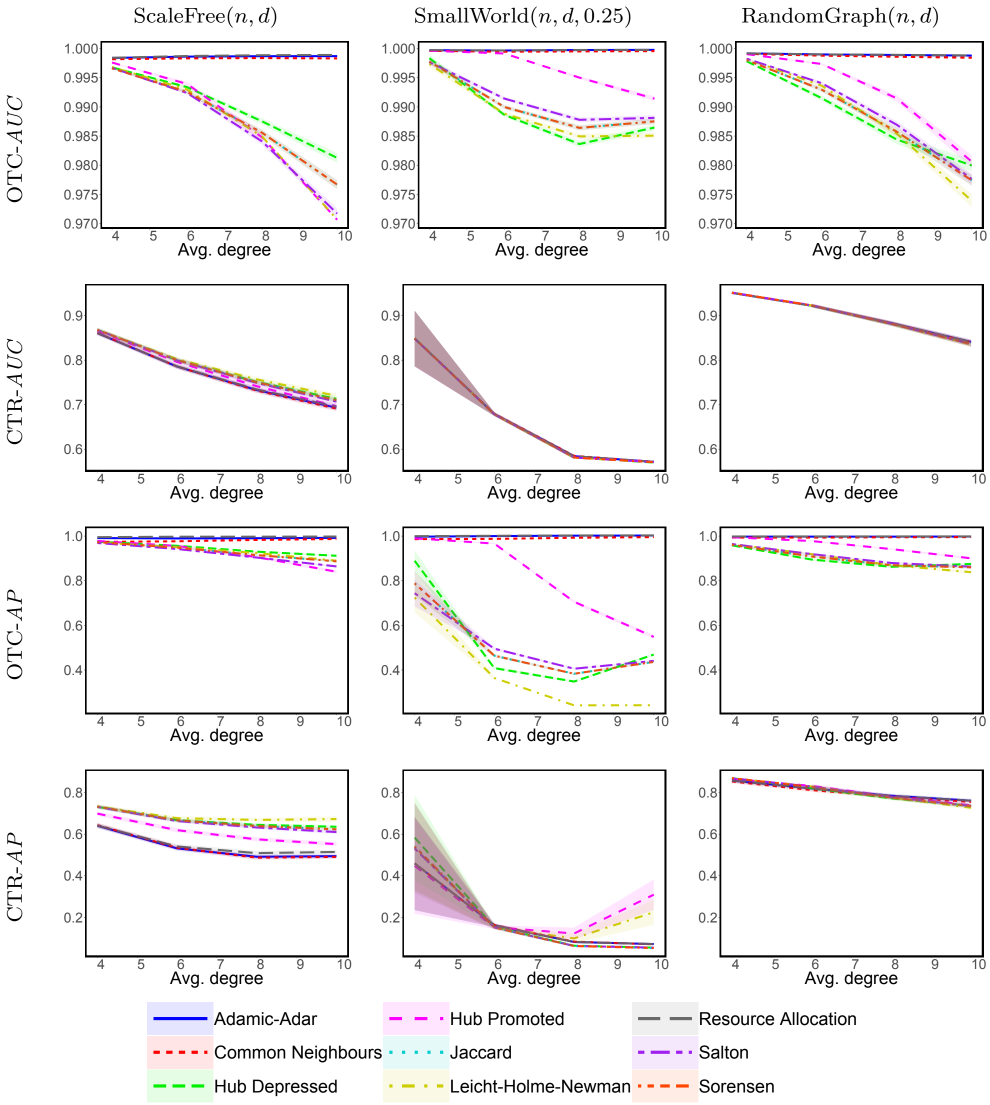


Figure S26: Evaluating the attack tolerance of the local similarity indices against OTC and CTR by measuring the relative change in $AUC$ and $AP$ while varying the **average degree**, $d$, in three types of networks: (i) ScaleFree$(n, d)$; (ii) SmallWorld$(n, d, 0.25)$; and (iii) RandomGraph$(n, d)$. For each $d$, we report the average over $n = 200, 400, 600, 800$. For any given $n$ and $d$, we run 50 experiments, each with 10 randomly-chosen evaders, none of which have less than 9 connections (to ensures that no evader is entirely diconnected when running CTR), where $b = 5$ and $H$ consists of 3 edges chosen randomly from the evader's connections. The experiment is repeated 5 times for each evader. Coloured areas represent 95% confidence intervals.

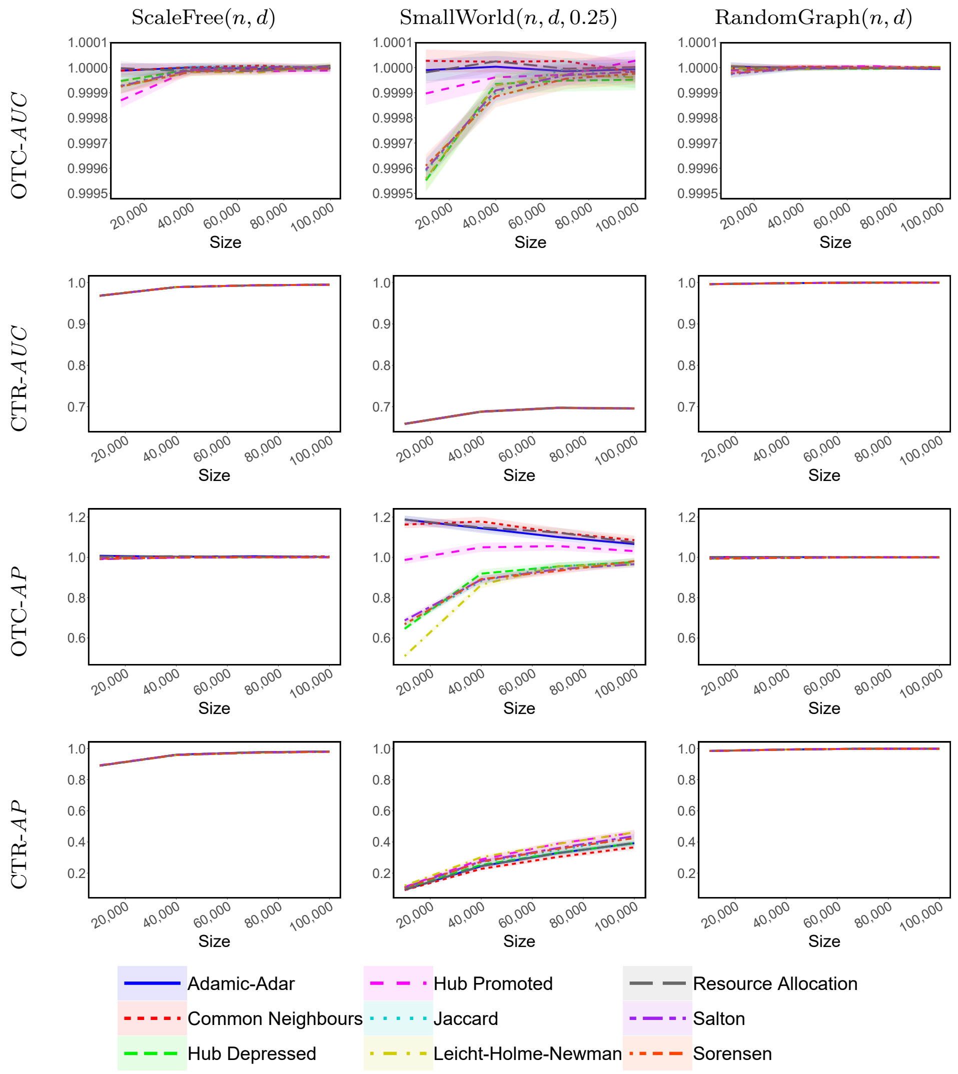


Figure S27: Evaluating the attack tolerance of the local similarity indices against OTC and CTR by measuring the relative change in $AUC$ and $AP$ while varying the **number of nodes**, $n$, in three types of networks: (i) ScaleFree($n, d$); (ii) SmallWorld($n, d, 0.25$); and (iii) RandomGraph($n, d$). For each $n$, we report the average over $d = 4, 6, 8, 10$. For any given $n$ and $d$, we run 50 experiments, each with 10 randomly-chosen evaders, none of which have less than 9 connections (to ensures that no evader is entirely diconnected when running CTR), where $b = 5$ and $H$ consists of 3 edges chosen randomly from the evader's connections. The experiment is repeated 5 times for each evader. Coloured areas represent 95% confidence intervals.

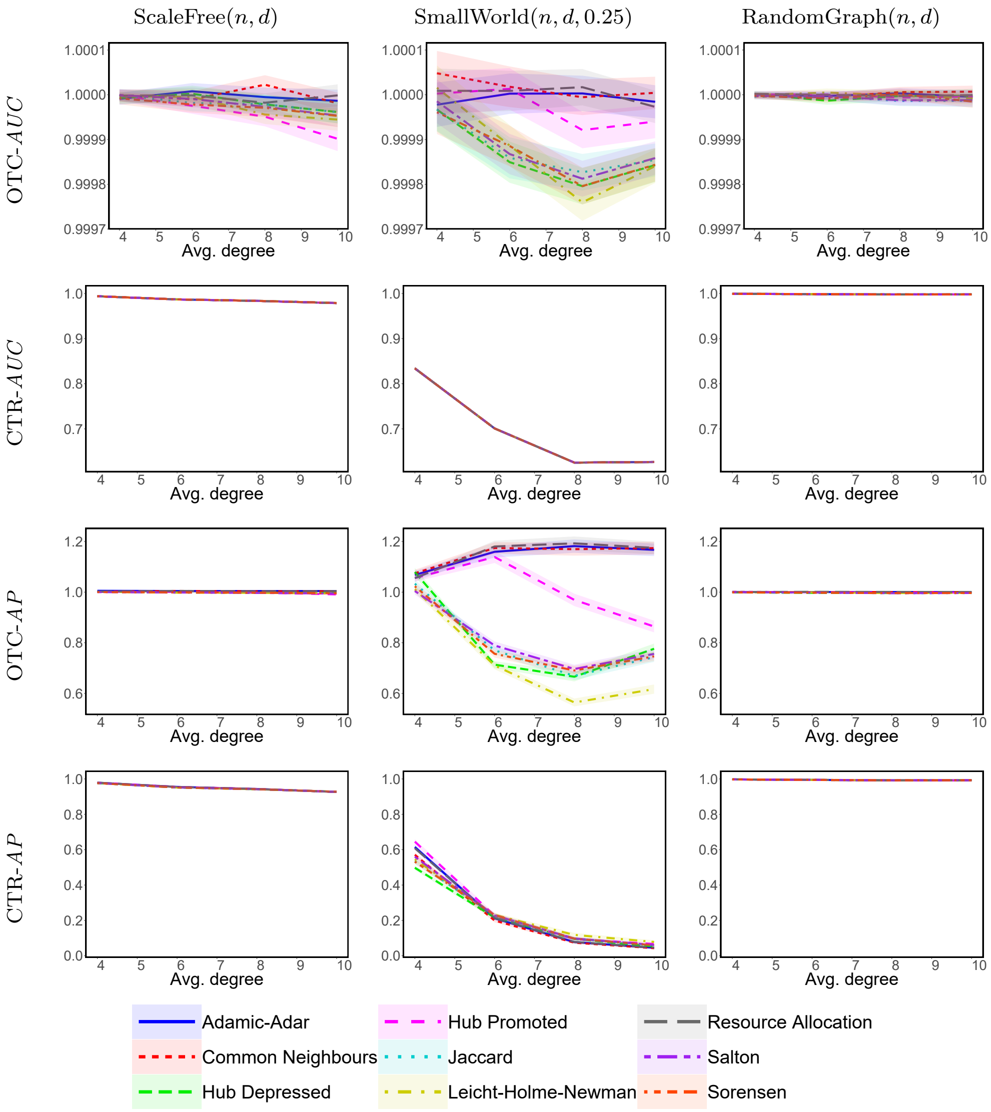


Figure S28: Evaluating the attack tolerance of the local similarity indices against OTC and CTR by measuring the relative change in $AUC$ and $AP$ while varying the **average degree**, $d$, in three types of networks: (i) ScaleFree($n, d$); (ii) SmallWorld($n, d, 0.25$); and (iii) RandomGraph($n, d$). For each $d$, we report the average over $n = 200, 400, 600, 800$. For any given $n$ and $d$, we run 50 experiments, each with 10 randomly-chosen evaders, none of which have less than 9 connections (to ensures that no evader is entirely diconnected when running CTR), where $b = 5$ and $H$ consists of 3 edges chosen randomly from the evader's connections. The experiment is repeated 5 times for each evader. Coloured areas represent 95% confidence intervals.

## S9 The Impact of the Hiding Process on the Network

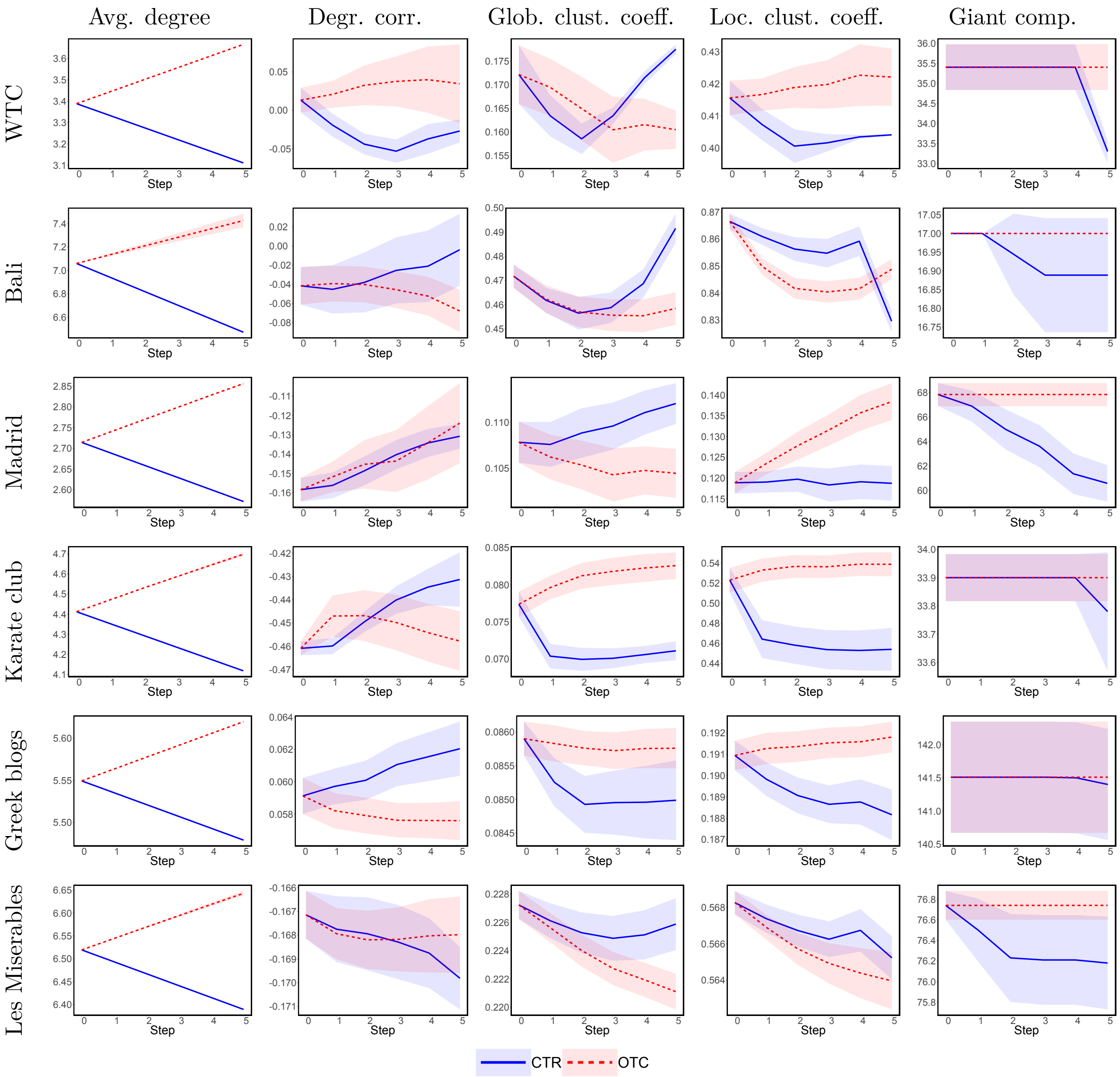


Figure S29: Quantifying the impact that our heuristics have on the properties of six networks: (i) **WTC 9/11 terrorist network**; (ii) **the Bali-attack network**; (iii) **the Madrid-bombing network**; (iv) **the Zachary karate club network**; (v) **the Greek political blog network**; (vi) **Les Misérables network**. The experiment is carried out for 10 randomly-chosen evaders, none of which have less than 9 connections (to ensures that no evader is entirely diconnected when running CTR). In each experiment, we have: $|H| = 3$ and $b = 5$, and the links in $H$ are chosen at random from the connections of the evader. The process is repeated 5 times for each evader. The impact on the network is measured in terms of (i) average degree; (ii) degree correlation; (iii) global clustering coefficient; (iv) local clustering coefficient; (v) the size of the giant connected component. Coloured areas represent the 95% confidence intervals.

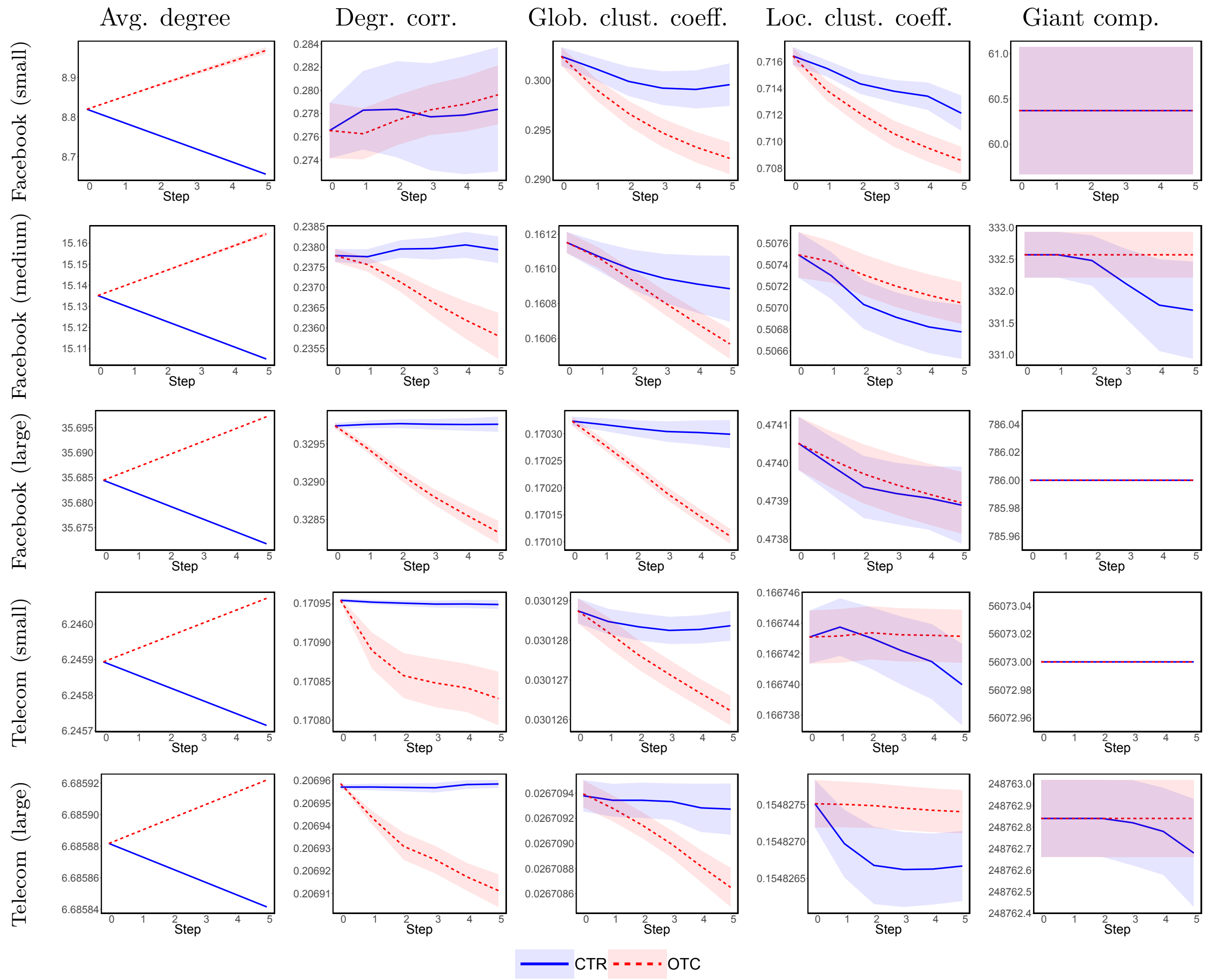


Figure S30: Quantifying the impact that our heuristics have on the properties of five networks: (i) **a small fragment of Facebook**; (ii) **a medium fragment of Facebook**; (iii) **a large fragment of Facebook**; (iv) **telecommunication (small)**; (v) **telecommunication (large)**. The experiment is carried out for 10 randomly-chosen evaders, none of which have less than 9 connections (to ensures that no evader is entirely diconnected when running CTR). In each experiment, we have: $|H| = 3$ and $b = 5$, and the links in $H$ are chosen at random from the connections of the evader. The process is repeated 5 times for each evader. The impact on the network is measured in terms of (i) average degree; (ii) degree correlation; (iii) global clustering coefficient; (iv) local clustering coefficient; (v) the size of the giant connected component. Coloured areas represent the 95% confidence intervals.

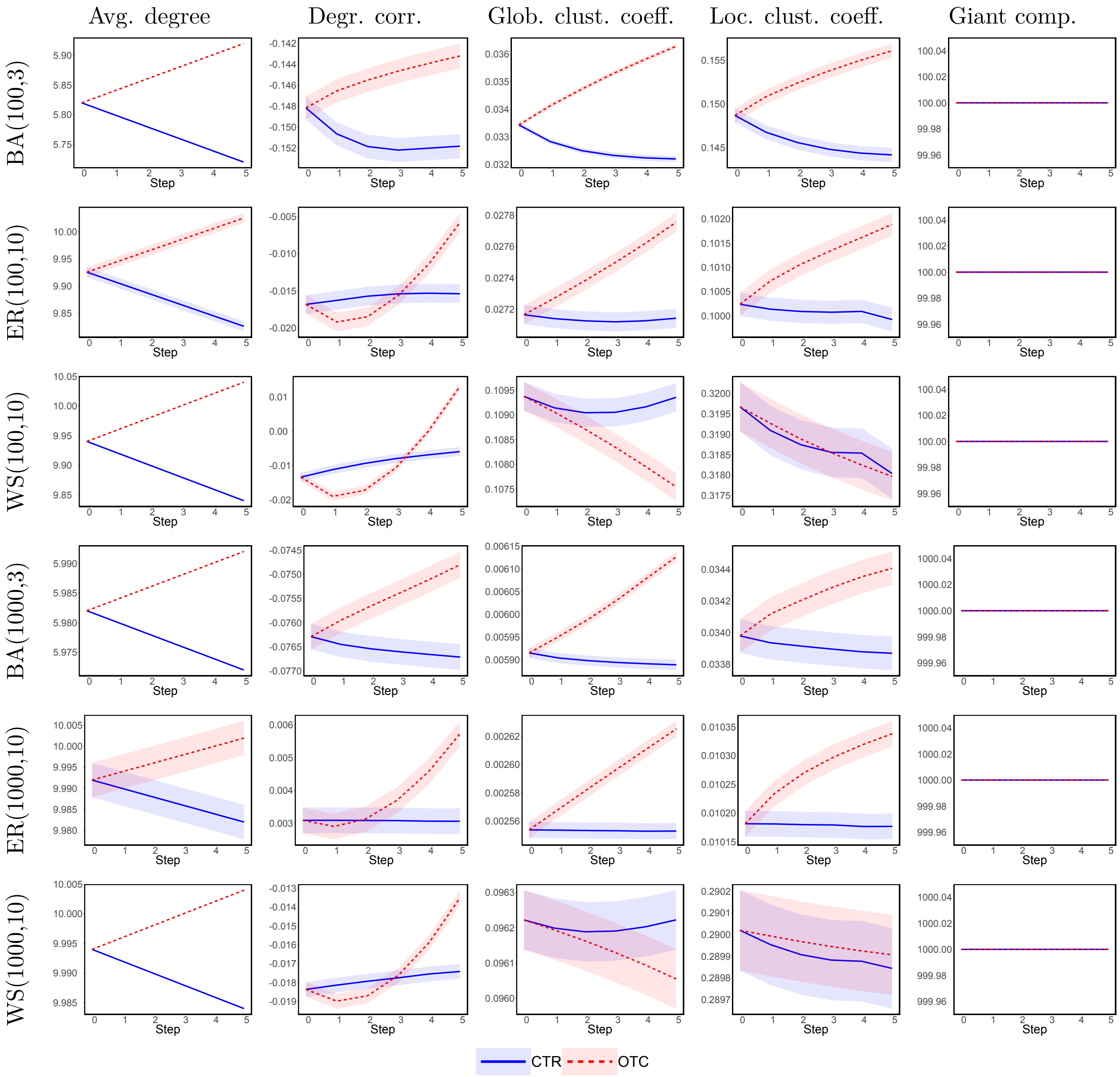


Figure S31: Quantifying the impact that our heuristics have on the properties of random networks generated using the following models: (i) **ScaleFree(100,3)**; (ii) **ScaleFree(1000,3)**; (iii) **RandomGraph(100,10)**; (iv) **RandomGraph(1000,10)**; (v) **SmallWorld(100,10,0.25)**; (vi) **SmallWorld(1000,10,0.25)**. The experiment is carried out for 10 randomly-chosen evaders, none of which have less than 9 connections (to ensures that no evader is entirely diconnected when running CTR). In each experiment, we have: $|H| = 3$ and $b = 5$, and the links in $H$ are chosen at random from the connections of the evader. The process is repeated 5 times for each evader. For each network type, the experiment is repeated over 50 randomly-generated networks. The impact on the network is measured in terms of (i) average degree; (ii) degree correlation; (iii) global clustering coefficient; (iv) local clustering coefficient; (v) the size of the giant connected component. Coloured areas represent the 95% confidence intervals.

# S10 The Impact of the Hiding Process on the Evader's Centrality

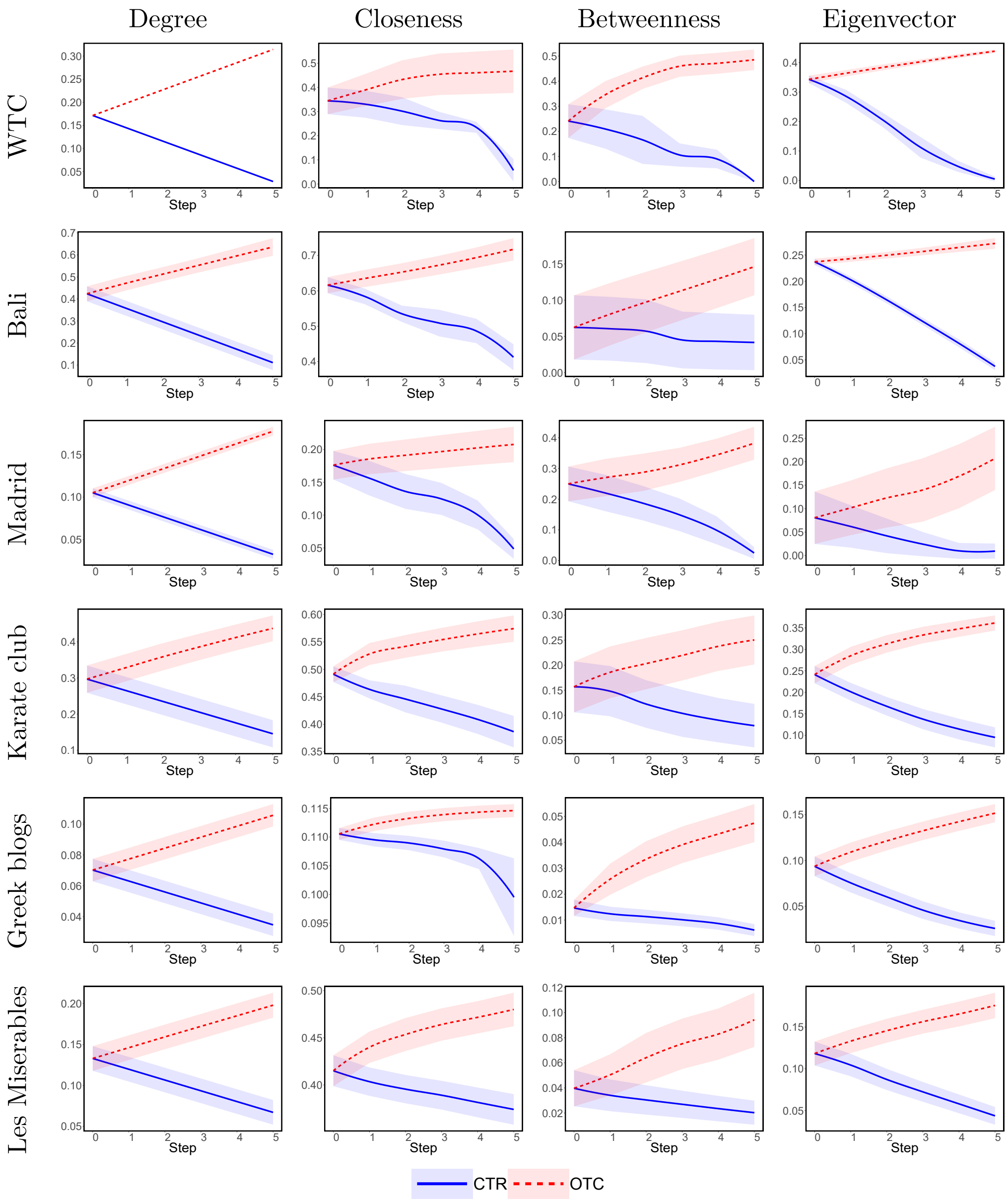


Figure S32: Quantifying the impact that our heuristics have on the evader's centrality in six networks: (i) **WTC 9/11 terrorist network**; (ii) **the Bali-attack network**; (iii) **the Madrid-bombing network**; (iv) **the Zachary karate club network**; (v) **the Greek political blog network**; (vi) **Les Misérables network**. The experiment is carried out for 10 randomly-chosen evaders, none of which have less than 9 connections (to ensures that no evader is entirely diconnected when running CTR). In each experiment, we have: $|H| = 3$ and $b = 5$, and the links in $H$ are chosen at random from the connections of the evader. The process is repeated 5 times for each evader. The impact on the network is measured in terms of (i) average degree; (ii) degree correlation; (iii) global clustering coefficient; (iv) local clustering coefficient; (v) the size of the giant connected component. Coloured areas represent the 95% confidence intervals.

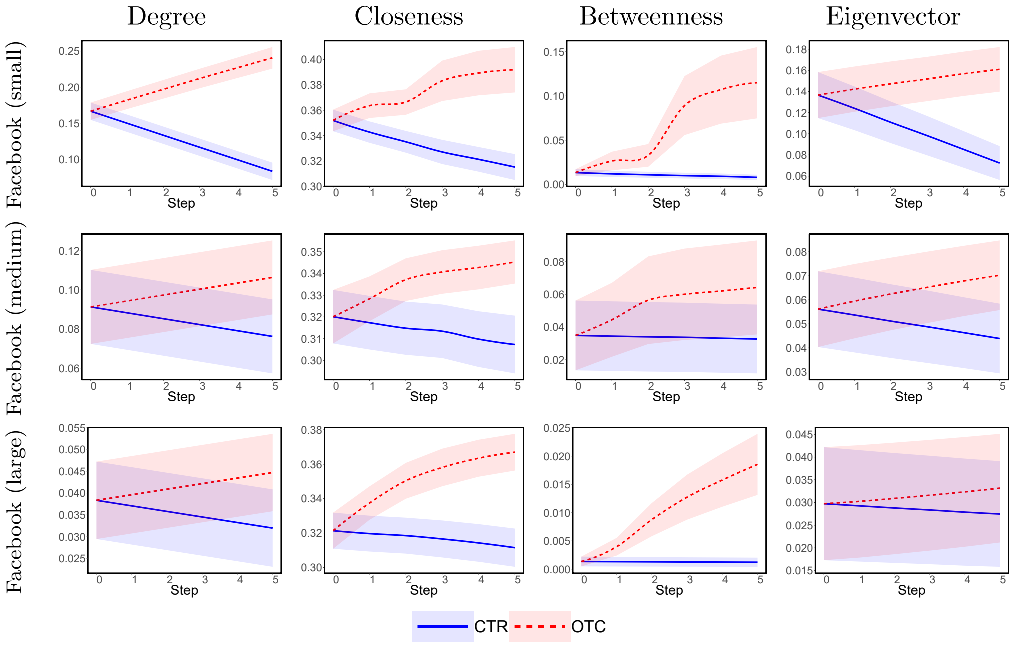


Figure S33: Quantifying the impact that our heuristics have on the evader's centrality in five networks: (i) **a small fragment of Facebook**; (ii) **a medium fragment of Facebook**; (iii) **a large fragment of Facebook**. The experiment is carried out for 10 randomly-chosen evaders, none of which have less than 9 connections (to ensures that no evader is entirely diconnected when running CTR). In each experiment, we have: $|H| = 3$ and $b = 5$, and the links in $H$ are chosen at random from the connections of the evader. The process is repeated 5 times for each evader. The impact on the network is measured in terms of (i) average degree; (ii) degree correlation; (iii) global clustering coefficient; (iv) local clustering coefficient; (v) the size of the giant connected component. Coloured areas represent the 95% confidence intervals.

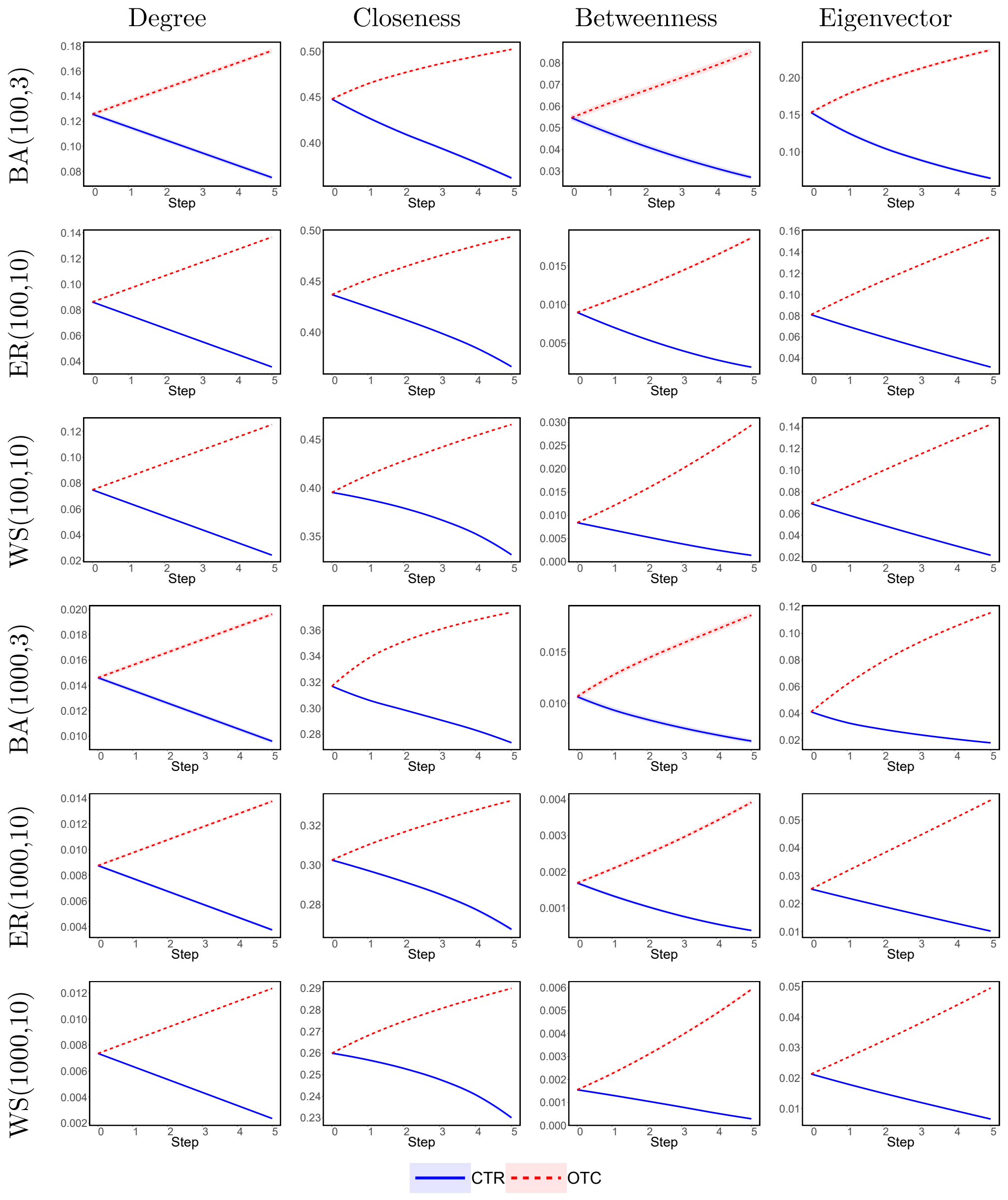


Figure S34: Quantifying the impact that our heuristics have on the evader's centrality in random networks generated using the following models: (i) **ScaleFree(100,3)**; (ii) **ScaleFree(1000,3)**; (iii) **RandomGraph(100,10)**; (iv) **RandomGraph(1000,10)**; (v) **SmallWorld(100,10,0.25)**; (vi) **SmallWorld(1000,10,0.25)**. The experiment is carried out for 10 randomly-chosen evaders, none of which have less than 9 connections (to ensures that no evader is entirely diconnected when running CTR). In each experiment, we have: $|H| = 3$ and $b = 5$, and the links in $H$ are chosen at random from the connections of the evader. The process is repeated 5 times for each evader. For each network type, the experiment is repeated over 50 randomly-generated networks. The impact on the network is measured in terms of (i) average degree; (ii) degree correlation; (iii) global clustering coefficient; (iv) local clustering coefficient; (v) the size of the giant connected component. Coloured areas represent the 95% confidence intervals.

# References


[1] L. A. Adamic and E. Adar. Friends and neighbors on the web. *Social networks*, 25(3):211–230, 2003.

[2] A.-L. Barabási and R. Albert. Emergence of scaling in random networks. *science*, 286(5439):509–512, 1999.

[3] K. Boyd, K. H. Eng, and C. D. Page. Area under the precision-recall curve: Point estimates and confidence intervals. In *ECMLPKDD*, pages 451–466. Springer, 2013.

[4] S. Brin and L. Page. The anatomy of a large-scale hypertextual web search engine. *Computer networks and ISDN systems*, 30(1-7):107–117, 1998.

[5] P. Chebotarev and E. Shamis. The matrix-forest theorem and measuring relations in small social groups. *arXiv preprint math/0602070*, 2006.

[6] T. H. Cormen, C. E. Leiserson, R. L. Rivest, and C. Stein. *Introduction to algorithms*, volume 6. MIT press Cambridge, 2001.

[7] P. Erdős and A. Rényi. On random graphs i. *Publ. Math. Debrecen*, 6:290–297, 1959.

[8] T. Fawcett. An introduction to roc analysis. *Pattern recognition letters*, 27(8):861–874, 2006.

[9] F. Fouss, A. Pirotte, J.-M. Renders, and M. Saerens. Random-walk computation of similarities between nodes of a graph with application to collaborative recommendation. *IEEE Transactions on knowledge and data engineering*, 19(3):355–369, 2007.

[10] L. Getoor and C. P. Diehl. Link mining: a survey. *ACM SIGKDD Explorations Newsletter*, 7(2):3–12, 2005.

[11] F. Göbel and A. Jagers. Random walks on graphs. *Stochastic processes and their applications*, 2(4):311–336, 1974.

[12] B. Hayes. Connecting the dots can the tools of graph theory and social-network studies unravel the next big plot? *American Scientist*, 94(5):400–404, 2006.

[13] P. Jaccard. *Etude comparative de la distribution florale dans une portion des Alpes et du Jura.* Impr. Corbaz, 1901.

[14] G. Jeh and J. Widom. Simrank: a measure of structural-context similarity. In *Proc. of the Eighth ACM SIGKDD*, pages 538–543. ACM, 2002.

[15] L. Katz. A new status index derived from sociometric analysis. *Psychometrika*, 18(1):39–43, 1953.

[16] B. Klimt and Y. Yang. The enron corpus: A new dataset for email classification research. In *European Conference on Machine Learning*, pages 217–226. Springer, 2004.

[17] D. E. Knuth. *The Stanford GraphBase: a platform for combinatorial computing*, volume 37. Addison-Wesley Reading, 1993.

[18] V. E. Krebs. Mapping networks of terrorist cells. *Connections*, 24(3):43–52, 2002.

[19] E. A. Leicht, P. Holme, and M. E. Newman. Vertex similarity in networks. *Physical Review E*, 73(2):026120, 2006.

[20] J. Leskovec and J. J. Mcauley. Learning to discover social circles in ego networks. In *Advances in neural information processing systems*, pages 539–547, 2012.

[21] L. Lü and T. Zhou. Link prediction in complex networks: A survey. *Physica A: Statistical Mechanics and its Applications*, 390(6):1150–1170, 2011.

[22] C. D. Manning and H. Schütze. *Foundations of statistical natural language processing*, volume 999. MIT Press, 1999.

[23] G. Miritello, R. Lara, M. Cebrian, and E. Moro. Limited communication capacity unveils strategies for human interaction. *Scientific reports*, 3:1950, 2013.

[24] M. E. Newman. Clustering and preferential attachment in growing networks. *Physical review E*, 64(2):025102, 2001.

[25] E. Ravasz, A. L. Somera, D. A. Mongru, Z. N. Oltvai, and A.-L. Barabási. Hierarchical organization of modularity in metabolic networks. *science*, 297(5586):1551–1555, 2002.

[26] G. Salton and M. J. McGill. *Introduction to modern information retrieval.* McGraw-Hill, Inc., 1986.

[27] J. Shawe-Taylor and N. Cristianini. *Kernel methods for pattern analysis.* Cambridge university press, 2004.

[28] T. Sørensen. {A method of establishing groups of equal amplitude in plant sociology based on similarity of species and its application to analyses of the vegetation on Danish commons}. *Biol. Skr.*, 5:1–34, 1948.

[29] B. Viswanath, A. Mislove, M. Cha, and K. P. Gummadi. On the evolution of user interaction in facebook. In *Proceedings of the 2nd ACM workshop on Online social networks*, pages 37–42. ACM, 2009.

[30] D. J. Watts and S. H. Strogatz. Collective dynamics of small-world networks. *nature*, 393(6684):440–442, 1998.

[31] W. W. Zachary. An information flow model for conflict and fission in small groups. *Journal of anthropological research*, pages 452–473, 1977.

[32] K. Zafiropoulos. Connectivity practices and activity of greek political blogs. *Future Internet*, 4(3):719–736, 2012.

[33] T. Zhou, L. Lü, and Y.-C. Zhang. Predicting missing links via local information. *The European Physical Journal B*, 71(4):623–630, 2009.
